# 4,5-Diaryl 3(*2H*)Furanones: Anti-Inflammatory Activity and Influence on Cancer Growth

**DOI:** 10.3390/molecules24091751

**Published:** 2019-05-06

**Authors:** Dmitrii Semenok, Jury Medvedev, Lefki-P. Giassafaki, Iason Lavdas, Ioannis S. Vizirianakis, Phaedra Eleftheriou, Antonis Gavalas, Anthi Petrou, Athina Geronikaki

**Affiliations:** 1 Skolkovo Institute of Science and Technology, Skolkovo Innovation Center, 3 Nobel Street, 143026 Moscow, Russia; dmitrii.semenok@skolkovotech.ru; 2 Moscow Institute of Physics and Technology, 9 Institutsky lane, 141700 Dolgoprudny, Russia; 3 Saint-Petersburg State University, Institute of Chemistry, Universitetskiy Prospekt, 26, 198504 Petergof, Russia; j2j3@yandex.ru; 4 Department of Pharmacology, School of Pharmacy, Aristotle University of Thessaloniki, 54124 Thessaloniki, Greece; lefkigiassafaki@gmail.com (L.-P.G.); iasonlavdas@pharm.auth.gr (I.L.); ivizir@pharm.auth.gr (I.S.V.); 5 Department of Medical Laboratory Studies, School of Health and Medical Care, Alexander Technological Educational Institute of Thessaloniki, 57400 Thessaloniki, Greece; eleftheriouphaedra@gmail.com; 6 Department of Pharmaceutical Chemistry, School of Pharmacy, Aristotle University of Thessaloniki, 54124 Thessaloniki, Greece; agavalas@pharm.auth.gr (A.G.); anthi.petrou.thessaloniki1@gmail.com (A.P.)

**Keywords:** 3(*2H*)furanones, phenanthro[9,10-b]furanones, cyclooxygenase, cytotoxicity, anticancer, MCF-7, HSC-3, gefitinib, 5-fluorouracil

## Abstract

Apart from their anti-inflammatory action, COX inhibitors have gathered the interest of many scientists due to their potential use for the treatment and prevention of cancer. It has been shown that cyclooxygenase inhibitors restrict cancer cell growth and are able to interact with known antitumor drugs, enhancing their in vitro and in vivo cytotoxicity. The permutation of hydrophilic and hydrophobic aryl groups in COX inhibitors leads to cardinal changes in the biological activity of the compounds. In the present study, thirteen heterocyclic coxib-like 4,5-diarylfuran-3(*2H*)-ones and their annelated derivatives—phenanthro[9,10-b]furan-3-ones—were synthesized and studied for anti-inflammatory and COX-1/2 inhibitory action and for their cytotoxic activity on the breast cancer (MCF-7) and squamous cell carcinoma (HSC-3) cell lines. The F-derivative of the –SOMe substituted furan-3(*2H*)-ones exhibited the best activity (COX-1 IC_50_ = 2.8 μM, anti-inflammatory activity (by carrageenan paw edema model) of 54% (dose 0.01 mmol/kg), and MCF-7 and HSC-3 cytotoxicity with IC_50_ values of 10 μM and 7.5 μM, respectively). A cytotoxic effect related to the COX-1 inhibitory action was observed and a synergistic effect with the anti-neoplastic drugs gefitinib and 5-fluorouracil was found. A phenanthrene derivative exhibited the best synergistic effect with gefitinib.

## 1. Introduction

Prostaglandins produced during inflammation are responsible for most of the undesirable effects of the inflammatory process and cyclooxygenase isoenzymes—COX-1 and COX-2—involved in their biosynthesis are the main targets of most anti-inflammatory drugs. As COX-2 is the isoenzyme induced during inflammation, COX-2-specific inhibitors have been developed during the last decades. Since 1999, when the first selective cyclooxygenase-2 (COX-2) inhibitors rofecoxib [1] and celecoxib [2] were launched, numerous research studies aiming to find effective and safe COX-2-associated anti-inflammatory drugs were performed. Despite the fact that in 2004 rofecoxib was withdrawn from the market due to an increased risk of cardiovascular events [3], an intensive study of properties and possible applications of coxibs continued for more than 20 years. 

Currently, COX inhibitors including even rofecoxib [4] are of renewed interest due to their potential use for the treatment and prevention of cancer. 

Cancer cells present cellular and genomic heterogeneity that contribute to mechanisms leading to drug resistance development and to therapy failure in clinical practice [5,6]. This heterogeneity is also modulated through interactions between malignant and normal cells that support the tumor microenvironment. Among the various hallmarks of cancer cells development, inflammation is a critical component of tumor survival, growth, and progression [7,8]. As a result, the development of selective anti-inflammatory drugs represents a promising target in cancer therapy. Inhibition of prostaglandin production by inactivation of the cyclooxygenase isoenzymes has been related in various ways with prevention of cancer cell growth, adhesion, migration and invasion [4,9,10,11,12,13,14,15,16]. As shown in the case of BrafV600E mouse melanoma [17], prostaglandin produced from the tumor cells may suppress immune response 

Among the two COX isoenzymes, COX-2 was mostly related with tumor development. The antiapoptotic role of COX-2 activity in chemoresistant cancer cells was noted in a study concerning drug resistance in patients with acute myeloid leukemia during standard treatment [18]. COX-2 is overexpressed in most cancer cells and its activity is decreased as a result of effective antitumor therapy [19,20]. In addition, COX-2 inhibition was found to reduce cancer cell development [16]. On the other hand, COX-1 is mainly expressed in certain tumor cells and cancer cell lines, such as the breast cancer MCF-7 cell line. Interestingly, transfected MCF-7 cells with more aggressive properties, overexpressed COX-2 isoenzyme [21]. Overexpression of COX-1 at the first phase of tumorigenesis, followed by overexpression of COX-2 at a later phase has been supported by certain researchers [19]. Although, most researches concerned mainly COX-2 inhibitors, COX-1 inhibition also may have application in anticancer therapy [19].

Most interestingly, COX inhibitors may act synergistically with known antitumor drugs, enhancing their in vitro and in vivo cytotoxicity [10,22,23,24]. It was found that celecoxib in combination with 5-fluorouracil (5-FU) increased the effectiveness of the latter against esophageal squamous cell carcinoma [25]. These synergistic effects motivated recent clinical trials of combination therapy of tumors, for the treatment of duodenal neoplasia (sulindac/erlotinib) [26] and breast cancer (etodolac) [27]. Moreover, combinations of aspirin or celecoxib with an anti-PD-1 monoclonal antibody resulted in a 1.5–2.1 fold decrease in melanoma growth rate in vivo [28]. 

**Figure 1:**
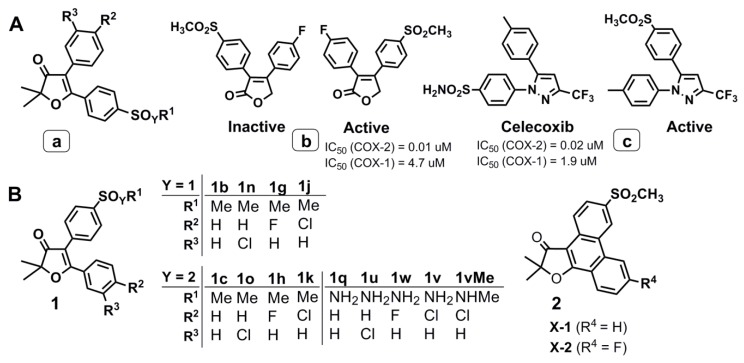
(**A**) Known 3(*2H*) furanones (a) [19,28], rofecoxib analogs (b), celecoxib and its regioisomer active analog (c) [29]. (**B**) Novel 3(*2H*)furanone (**1**) and phenanthro[9,10-b]furan-3-one (**2**) derivatives.

Previous studies by S. Shin and coworkers showed that regioisomeric 5-aryl-2,2-dialkyl-4-phenylfuran-3(*2H*)-one derivatives (Figure 1A) have IC_50_ values of COX-2 inhibitory action comparable to that of rofecoxib [29,30]. Taking all these into account we designed and synthesized a number of novel 4,5-diarylfuran-3(*2H*)-one and phenanthro[9,10-b]furan-3-one derivatives (Figure 1B) with the aim of obtaining novel COX inhibitors. The anti-inflammatory and antitumor effects, as well as synergistic effects of the compounds, were tested and the effects of different aryl moieties in the total yield and in the activities of the compounds are discussed. 

## 2. Results and Discussion

### 2.1. Design and Computer-Aided Prediction of COX-1/2 Inhibitory Activity

Since COX-2 is the main isoenzyme overexpressed in inflammation and mostly related to cancer development, COX-2 inhibition was among the desired properties of the designed compounds. The design and selection of the compounds was performed using a computer-aided approach.

Crucial structural differences between the shape of the active site of COX-1 and COX-2 are responsible for the effectiveness of COX-2 selective inhibitors. The replacement of isoleucines at position 523 and 434 of COX-1 isoenzyme by the smaller valine in COX-2 leads to the creation of a secondary pocket in the COX-2 active site [31]. The scaffold of cis-1,2-diaryl-alkene can be easily placed in the larger cavity of COX-2 isoenzyme but not in COX-1, so this scaffold should be suitable for selective inhibition of COX-2 [32,33]. Since, a large number of biological studies of different classes of human cyclooxygenase inhibitors have been carried out [34] and a considerable array of experimental data on COX-1/2 inhibition accumulated, we attempted to analyze the expected inhibitory activity of the designed compounds **1**–**2** by molecular docking analysis before the experimental evaluation of the compounds.

As the novel compounds were designed to have structural similarity to the coxib family, protein structures of the COX-1 and COX-2 enzymes in complex with celecoxib were selected for Docking Analysis. The *Ovis aries* COX-1 and 3KK6 [35] and *Mus musculus* COX-2 and 3LN1 [36] were obtained from the Protein Data Base (PDB). However, since both structures were not human, the 3D structures of the human isoforms were constructed using these structures as templates. The developed structures were used to predict the ability of thirteen known COX-1/2 inhibitors (E1–E14, Figure S1) [37] to form stable complexes with enzymes using docking analysis. The binding energy was predicted for each of the compounds E1–E14 and their correlation with the experimentally calculated IC_50_ value of inhibitory action was calculated. 

**Figure 2:**
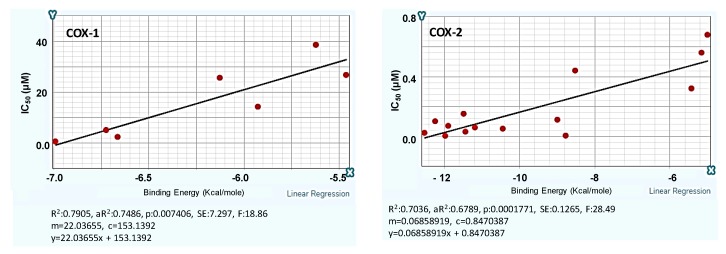
Correlation of the predicted binding energy in kcal/mole with the experimentally calculated IC_50_ value of inhibitory action of the reference compounds E1–E14. against COX-1 (**left**) and COX-2 (**right**).

Linear correlation was obtained with R^2^ = 0.791 for COX-1 and R^2^ = 0.704 for COX-2 (Figure 2). 

The same enzyme structures were then used for the calculation of the binding energy for the designed compounds (Figure 1). Based on the calculated binding energies and the correlations obtained from the reference test set, an estimation of the expected inhibitory action of the designed compounds was made. The results are shown in Table 1.

According to the results, all compounds were expected to be COX-2 selective inhibitors with high inhibitory action. As clearly shown in Table 2, compound **1o** was predicted to be the most potent COX-2 inhibitor with the best selectivity, with compound **1j** being the best ligand towards COX-1 isoform. Based on these estimations, it was decided to proceed with the synthesis of all the designed compounds.

### 2.2. Chemistry

#### Synthesis

**Scheme 1:**
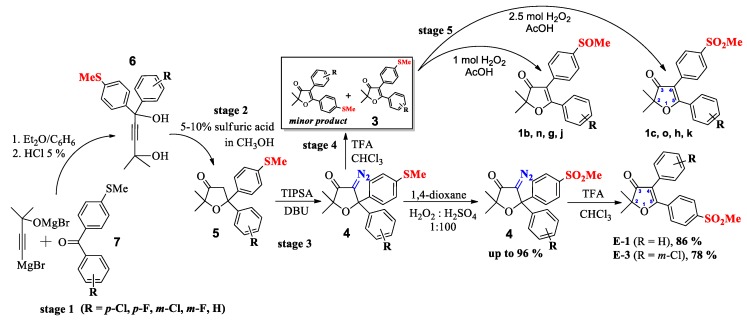
Synthesis of methylsulfones and methylsulfoxides of 4,5-diarylfuran-3(*2H*)-ones (**1**) as well as reference compounds **E-1** and **E-3**. Oxidative reactions require a catalyst—sulfuric acid.

Synthesis of 4,5-diarylfuran-3(*2H*)-ones and their annelated derivatives involved 5–7 stages [38,39]. The preliminary step of the synthesis was Friedel–Crafts reaction between thioanisole and benzoyl chloride in CH_2_Cl_2_, catalyzed by anhydrous aluminium chloride to furnish the 80–90% yield of ketone **7** (see Supporting Information, Scheme S1, and Table S1). The first step involved nucleophilic addition of the Grignard reagent derived from 2-methyl-but-3-yn-2-ol to ketone **7** to produce diol **6** in yield up to 98% (Table 2, Scheme 1).

At the second stage, diol **6** was transformed into corresponding monoketone **5** by intramolecular cyclization in 5–10% sulfuric acid/methanol solution and 2,2-dimethyl-5,5-diaryldihydrofuran-3(*2H*)-one **5** formed can be used without purification on the next stage. 

The third step (Scheme 1) was transformation of monoketone **5** into diazoketone **4** (racemic mixture) by diazo transfer reaction with DBU as base. Acid-catalyzed decomposition of diazoketones **4** [38,39] yielded a mixture of corresponding regioisomeric furan-3(*2H*)-ones **3** with excellent total yields of 95 to 99% (stage 4). The ratio of regioisomers strongly depends on substitution of benzene rings: predominantly migrates donor-substituted aryl [40]. Regioisomeric products of migration can be distinguished by position and intensity HNMR signals of aryl protons, e.g., by comparison of downfield doublets of *o*-protons in *p*-XPh (X = Cl, F, SO_n_Me), which can be compared directly for both regioisomers. HNMR spectra of different regioisomers were previously published [41,42,43]. Complete oxidation of thiomethyl group by hydrogen peroxide required a catalyst—sulfuric acid. Compounds E-1 and E-3 (Scheme 1) were prepared by oxidation of thiomethyl group in the minor products of decomposition of corresponding diazocompounds (**4**).

The yields of desired products are presented in Table 2 and Table 3 (stage-wise) and Table 4 (total).

Another branch of Scheme 1(stage 5) led to 5-(4′-(methylsulfonyl or sulfinyl)phenyl)-4-aryl-furan-3(*2H*)-ones obtained after full or partial oxidation of key intermediate 5-(4′-(methylthio)-phenyl)-4-arylfuran-3(*2H*)-ones purified by recrystallization. It should be noted that the mixture of regioisomeric furan-3(*2H*)-ones formed on the 1,2-aryl migration stage could not be easily separated by chromatography. 

**Scheme 2:**
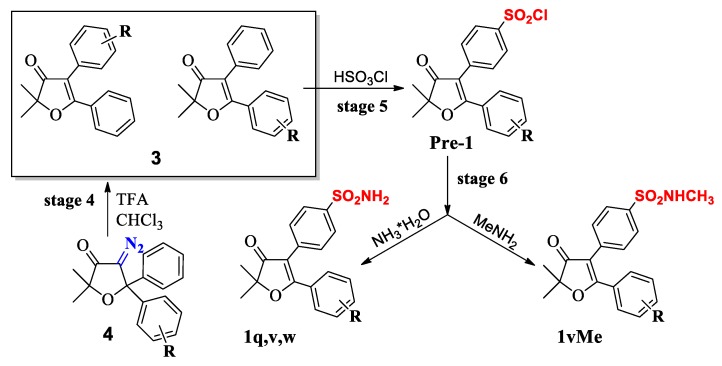
Synthesis of 4,5-diarylfuran-3(*2H*)-ones sulfonamides **1**.

Synthesis of sulfonamides (Scheme 2) differs from the synthesis of sulfones and sulfoxides starting from stage 5. The mixture of regioisomeric diazoketone **4** decomposition products (stage 4, Scheme 2) without separation was subjected to chlorosulfonation with 10-fold excess of chlorosulfonic acid for 24 to 48 h at r.t. Surprisingly, only one of the regioisomers was capable of chlorosulfonation in the *para*-position of the unsubstituted 4-Ph ring. It is interesting to note that the standard Friedel–Crafts substitution in this ring did not occur with any of typical catalysts. As a result of this highly selective reaction, a mixture of the desired sulfonyl chloride and one of the migration products was formed after hydrolysis of the excess chlorosulfonic acid with ice. However, their separation was still a difficult task. Therefore, the mixture without further purification was treated with NH_3_ solution in water/THF. 

As a result of the conversion of the SO_2_Cl group to SO_2_NH_2_, the polarity of the compound changed sharply and sulfonamide obtained was easily separated chromatographically from the regioisomeric diazoketone 4 decomposition product. Comparison of NMR spectra of the products and known structures of 1,2-migration products allows the unambiguously determination the structure of sulfonamides obtained.

**Scheme 3:**
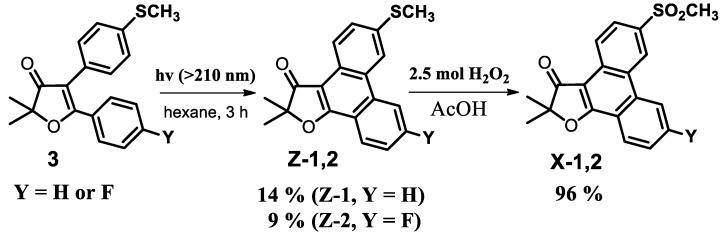
Synthesis of phenanthro[9,10-b]furanones (**2**) by photochemical cyclization of the stilbene moiety of 4,5-diaryl-3(*2H*)furanones **1**.

Synthesis of phenanthro[9,10-b]furanones (Scheme 3) was carried out using key intermediate **3**, which was subjected to irradiation with hard ultraviolet light (2–3 h) in dilute solution in hexane, accompanied by intramolecular oxidative cyclization of the stilbene fragment [44]. Despite low conversion (~15%) the yield of product was increased up to 25% by respective chromatographic separation of phenanthrofuranones and irradiating the depleted reaction mixture again (see Supporting Information).

The main difficulties in the synthesis arose for *p*-Cl, and, especially, for *m*-F-substituted compounds, for which only the ratio of products after 1,2-aryl migration was established. 

### 2.3. Biological Evaluation

#### 2.3.1. In Vitro Evaluation of COX-1/2 Inhibitory Action

The COX-1/2 inhibitory action, mainly of the most active and COX-2 selective inhibitors according to the prediction results, was evaluated in vitro using ovine COX-1 and human recombinant COX-2 isoenzymes included in the COX inhibition assay kit of Cayman as described at the experimental part. Specifically, compound **1o** with the higher predicted COX-2 activity as well as the other two most selective compounds, **1h** and **1k** (predicted selectivity: 100–125), of the –SO_2_CH_3_ group were tested. The less selective compound **1c** of the same group was also tested for comparison. Furthermore, the most selective, **1g,** and the less selective, **1j,** compounds of – SOCH_3_ group as well as the two most selective compounds of the 

SO_2_NH_2_ group were also tested. The results are shown in Table 5 and Table 6. 

Despite the coxib like structure and the prediction results which indicated a preference in COX-2 inhibition, most of the compounds exhibited mainly COX-1 inhibitory action with the exception of compound **1v** which showed a slightly lower IC_50_ value for COX-2 compared to COX-1 isoenzyme (20 μΜ vs. 28 μΜ). Compound **1v**, the *p*–Cl derivative of the –SO_2_NH_2_ substituted compounds was the most active COX-2 inhibitor (IC_50_ = 20 μΜ) followed by the *m*–Cl derivative **1o** (IC_50_ = 71 μΜ) of the –SO_2_ CH_3_ substituted compounds.

In contrast, compound **1g,** the *p*–F derivative of the –SOMe substituted group was the best COX-1 inhibitor with an IC_50_ value of 2.8 μΜ followed by two compounds of the –SO_2_ CH_3_ Me substituted group, **1o** and **1k** with IC_50_ values 22 μΜ and 29 μΜ respectively and two compounds of the –SO_2_NH_2_ substituted group **1v** and **1w** with IC_50_ values of 28 μΜ and 70 μΜ. Polarity of the substituents of both aryl moieties influences the activity as shown by the comparison between the IC_50_ values of the *p*–F derivatives **1g** and **1w** where the less polar –SO CH_3_ Me group appears to favor inhibitory activity compared to the –SO_2_NH_2_ group. On the other hand, introduction of the less electronegative Cl atom at the *p*-position of the phenyl ring in the –SO_2_NH_2_ derivatives (**1v**) resulted in enhanced inhibition of both isoenzymes compared to the strongly electronegative F-substituted **1w**. It was found that none of the substituents favor COX-2 selective inhibition. However, compound **1v** appears to be a better COX-2 inhibitor than is the reference drug naproxen. 

It should be mentioned that in general, the docking analysis failed to predict the inhibitory action of the compounds especially in case of COX-1 as well as their selectivity. This may mean that the human protein 3D structures used for the prediction are not appropriate. Docking analysis using other COX-1 protein structures such as the ovine 1EQH and 2AYL structures co-crystalized in complex with flurbiprofen and the ovine 4O1Z in complex with meloxicam did not show better correlation with the in vitro activity. COX-2 inhibition was better predicted in all cases. Docking analysis results using the human COX-2 structure 5IKT derived from its complex with tolfenamic acid are presented in Table 6. According to the 5IKT based docking analysis **x-1** was expected to be one of the best COX-2 isoenzyme inhibitors.

**Figure 3:**
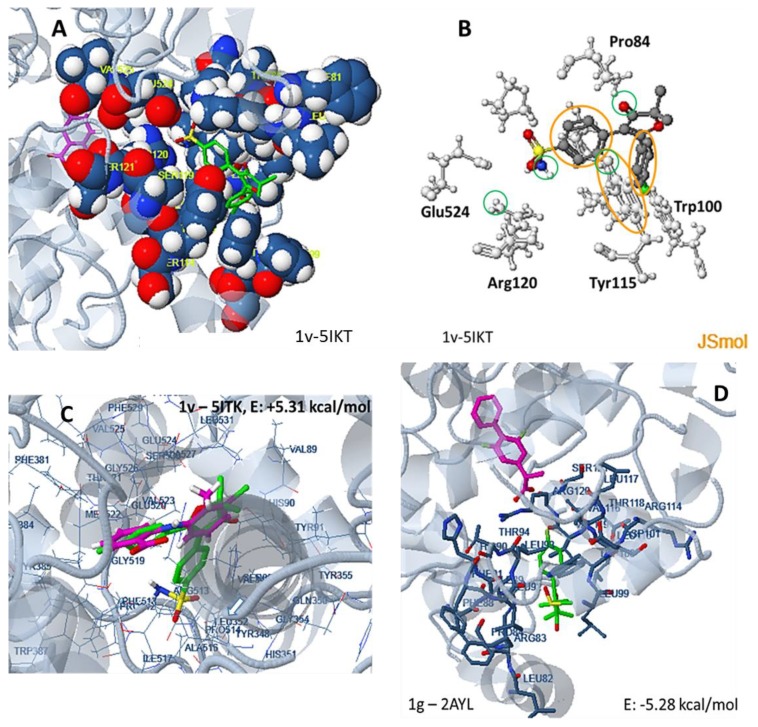
Docking of compound **1v** to the human COX-2 structure 5IKT, at a lower energy complex (**A**,**B**) with the compound docked at the entrance of the active site and at a high energy complex with the compound oriented in a deeper space of the active site (**C**). Docking of compound **1g** to the ovine COX-1 structure 2AYL (**D**). Studied compound in green, initial ligand in magenta. Yellow cycles: pi–pi interactions, green cycles: polar interactions. Polar interactions are also formed between Arg120 and the –SO_2_ NH_2_ group.

Interestingly, docking analysis indicate that all studied compounds may form stable complexes with the human COX-2 or the ovine COX-1 structures which were used. However, they are not placed deep inside the active site pocket as observed with most known inhibitors but are preferably oriented near the entrance of the active site (Figure 3). Much higher binding energies were calculated in case of deeper orientation.

As shown in Figure 3B, pi–pi interactions between Tyr115 of the COX-2 structure 5IKT and both aryl moieties of compound **1v** are formed while Tyr115 also participates in polar interactions with the O atom of the furanone ring. Similar interactions are observed in the case of all compounds stabilizing their orientation at the entrance of the active site cleft, including the more rigid phenanthrene derivatives. On the other hand, a negative effect leading to the formation of a high energy complex (−5.31 kcal/mol) is observed when the compound is oriented deep inside the active site, mainly because of the hydrophobic interactions between Leu352 (Figure 3C, arrow) and both aryl groups of the molecule.

Polar interactions with Arg 120 and hydrophobic interactions with Ile89, Leu112 and Val116 are involved in complex stabilization between compound **1g** and the COX-1 structure 2AYL (Figure 3D). 

The inability of the molecules to be placed at the inner part of the active cleft may be explained by the volume and rigidity of the molecules. However, the observed negative interactions may be enhanced by the fact that the protein structures are derived from complexes with smaller molecules. It is well known that enzymes are flexible molecules that conformationally adapt to the substrate or inhibitor, and that the volume of the active site cavity is restricted when small molecules have been used for co-crystallization. 

#### 2.3.2. In Vivo Anti-Inflammatory Activity

For the evaluation of anti-inflammatory activity of the tested compounds in vivo carrageen-induced mouse paw edema assay was used, with celecoxib (64.6%) and indomethacin (47%) as reference compounds. In general compounds in concentration of 0.1 mmol/g exhibited inhibition of inflammation varying between 38–57.7%. From the data of Table 7 it is obvious that anti-inflammatory activity of alkylsulfones and sulfoxides of furan-3(*2H*)-ones, including annelated derivatives are very similar.

Thus, the study of structure–activity relationship revealed that anti-inflammatory activity of the compounds tested is almost independent of the nature and position of substituents. It should be noted that COX-1/COX-2 inhibition does not correlate with anti-inflammatory activity, as has been observed in anti-inflammatory results of other researches. Furthermore, since other mechanisms are involved in the first steps of edema formation, linear correlation between in vivo and in vitro results cannot be expected to be found. 

#### 2.3.3. Anticancer Activity against MCF-7 Breast Cancer Cells

The anticancer treatments traditionally were based on the inhibition of DNA synthesis and function. Nowadays, many researchers turn their interest to selective inhibition of signaling pathways involved in proliferation, as another approach for anticancer therapy. Epidermal growth factor receptor (EGFR), which is overexpressed in major number of human tumors, plays a significant role in growth signaling. One representative of potent and selective EGER inhibitors is gefitinib, which is a competitive inhibitor of the ATP binding site of EGFR tyrosine kinase.

Gefitinib is an antineoplastic drug well known for its inhibitory effect in cell proliferation and induction of cell death in various cancer cell lines, including breast cancer. It inhibits the catalytic activity of numerous tyrosine kinases and may also induce cell cycle arrest and inhibit angiogenesis [45,46].

Three compounds were selected for the evaluation of their effect on cancer cell growth in the human epithelial breast adenocarcinoma cell line (MCF-7 cells) and in human tongue squamous cell carcinoma HSC-3 cell line. The *p*–F derivative of the SO CH_3_ group (**1g**) with the best COX inhibitory action (COX-1 IC_50_:2.8 μM), the *p*–F derivative of SO_2_CH_3_ group (**1h**) with low COX inhibitory action and one compound from the phenanthren group (**x-1**) with moderate predicted inhibitory action on both COX-1/2 isoenzymes and the best predicted selectivity over COX-2 were chosen. 

The cells were grown in the presence of each of the selected compounds as well as of the two well-known anti-neoplastic drugs gefitinib and 5-fluorouracil. Celecoxib, a selective COX-2 inhibitor was used as a control. The IC_50_ values of each compound after 48 h exposure of the cells were determined.

**Figure 4:**
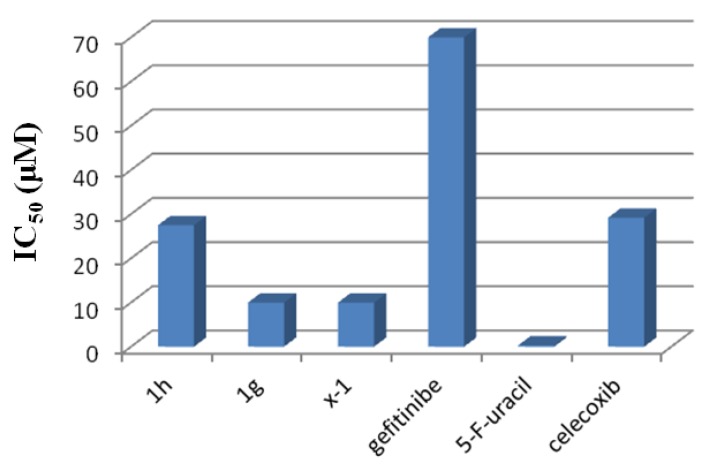
IC_50_ values of the new synthesized compounds as well as gefitinib, 5-fluorouracil, and celecoxib.

Interestingly, all three compounds inhibited the MCF-7 cell growth (Table 8, Figure S2) with an IC_50_ value of 24 µM for **1h** and 10 µM for **1g** and **x-1**, while the IC_50_ values of the control compounds were 70 µM for gefitinib, 0.03 µM for 5-fluorouracil, and 29.2 µM for celecoxib (Figure 4). Two of the studied compounds exhibited higher cytotoxicity at MCF-7 cell line than did gefitinib (IC_50_:70 μΜ), with the less active COX inhibitor, **1h,** to exhibit similar cytotoxicity under the culture conditions (IC_50_:24 μΜ). As far as the HSC-3 cell line is concerned, compound **1g** exhibited remarkable growth inhibition of this cell line (64%) at the concentration of 10 μΜ with an IC_50_ value of 7.5 μΜ. The two other compounds exhibited much lower cytotoxicity at the HSC-3 cells. The inhibition potency of celecoxib and 5-fluouracil is comparable with that presented in the literature [25]. The results obtained for synergistic activity are presented in Table 8, Figure 4. 

**Figure 5:**
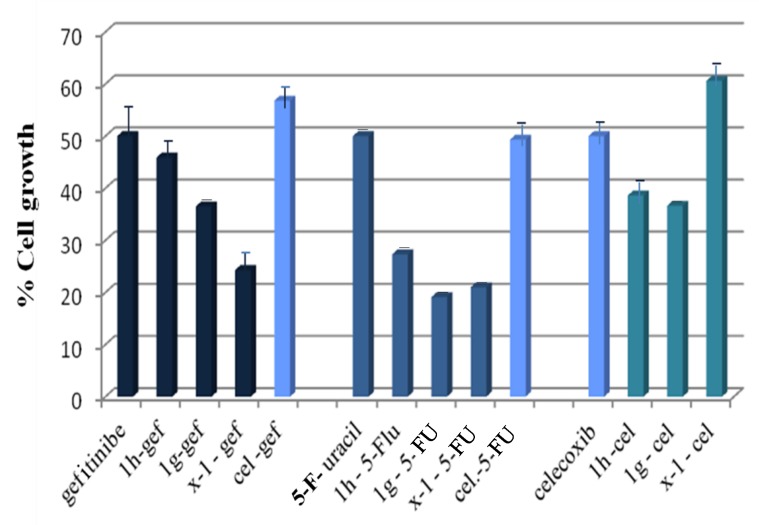
% MCF-7 cell growth rates after cell exposure to the combinations of each COX-2 inhibitor with gefitinib, 5-fluorouracil, or celecoxib in their IC_50_ or lower concentrations, for 48 h. Cell growth was assessed using a hemocytometer (Neubauer chamber) and is expressed as a percentage (%) relative to that for the untreated, control culture (CTL). The data represent mean values of four.

**Figure 6:**
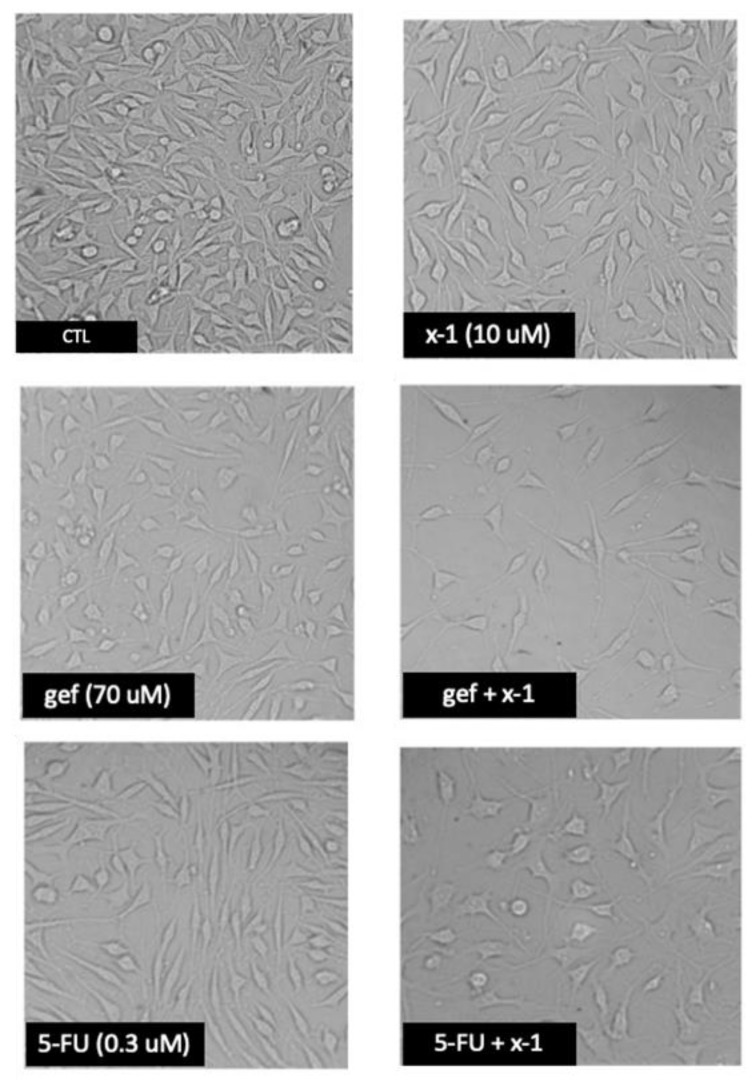
Phase-contrast microscopy images (32x) after MCF-7 cell treatment with the single antineoplastic drugs and synthesized compounds as well as with the combinations of drugs and compounds, for 48 h.

In addition, exposure of the MCF-7 cells to each of the compounds in combination with each one of the two anti-neoplastic drugs was performed in order to study the synergistic effect of the compounds. The results are shown in Table 9 and Figure 5 and Figure 6. 

The two more active compounds—**1g** and **x-1**—further decreased cell growth when added in combination with gefitinib. Calculation of the Combination Index (CI) revealed a synergistic effect with CI = 0.78 and CI = 0.26 for compounds **1g** and **x-1**, respectively. The low activity compound **1h** exhibited antagonistic effect with CI = 1.95. Compound **1g** also showed a remarkable synergistic effect when added in combination with 5-FU (CI = 0.058). The low activity compound **1h** showed a lower synergistic effect (CI = 0.533), while compound **x-1** exhibited antagonistic effect. On the other hand, the known COX-2 selective inhibitor, celecoxib showed antagonistic effect with both anti-neoplastic drugs gefitinib or 5-fluorouracil with CI = 9.8 and CI = 1.57, respectively. This has also been observed elsewhere. According to previously published results, the synergistic effect of celecoxib depends on the cell culture as well as on the concentration of the anticancer drug used [25]. Decrease instead of enhancement of the anticancer effect has been mentioned in an experiment concerning cis-platin [47].

Celecoxib was also used in combination with each one of the new synthesized compounds (**1h, 1g**, and **x-1**). A synergistic effect with the most active COX-1 inhibitor **1g** was observed (CI = 0.75), while with the two other compounds **1h** and **x-1** celecoxib showed antagonistic effect with CI = 1.32 and CI = 1.90, respectively. 

In conclusion, the most active and COX-1 selective inhibitor, **1g**, exhibited synergistic effect with both anti-neoplastic drugs probably due to overexpression of COX-1 in MCF-7 cells [48]. The synergistic effect of compound **1g** and celecoxib may be indicative for the involvement of both cyclooxygenase isoenzymes in cancer cell development and the value of combined COX1/2 inhibition for best inhibitory effect. Combined treatment with specific COX-1 and COX-2 inhibitors has been found to have better results in cell culture experiments, including the MCF-7 cell line, by other researchers as well [49].

The most active compounds—**1g** and **x-1**—belonged to the -SOMe derivatives of the 4,5-diaryl-furan-3(*2H*)-ones and to the phenanthrene derivatives, respectively. Usually sulfoxides are used to study cytotoxicity as ligands in complexes of rhodium and platinum [50]. The antitumor activity of sulfoxides has been studied earlier [51] against Ehrlich ascites brain cells where the sulfoxides and sulfones exhibited an approximately equal level of activity, and the IC_50_ value was comparable to that of 8-azaguanine. Phenanthrofuranones are a quite rare class of molecules in studies of anti-inflammatory and anticancer activity although some phenanthrofurane quinone derivatives have been isolated from the roots of *S. miltiorrhiza* [52] and from *Pleione bulbocodioides*—a medicinal plant with anti-inflammatory properties that is used in traditional Chinese medicine [53].

## 3. Materials and Methods

All the chemicals used were of analytical grade and commercially available. The kit for COX Activity Assay was purchased from Cayman (Ann Arbor, MI, USA).

### 3.1. Computer Simulation Methods

#### 3.1.1. Preparation of Protein Structures and Docking Analysis Based on *Ovis aries* COX-1, 3KK6 and *Mus musculus* COX-2, 3LN1

Homology modeling and preprocessing were performed in ICM Pro Software. 3D Molecular docking was carried out using MOE v.2014.09.01 (Chemical Computing Group, www.chemcomp.com, Köln, Germany). 

RSA data were obtained from the PDB for the structures of *Ovis aries* COX-1 (3KK6 [35]) and *Mus musculus* COX-2 (3LN1 [36]) in complex with Celebrex and were used for homology modeling. As a result, the 3D structures of the corresponding human isoforms were constructed using these structures as templates (*h*COX-1, FASTA code: sp_P23219_PGH1_HUMAN [54]) and *h*COX-2, FASTA code: sp_P35354_PGH2_HUMAN [55]). To simplify modeling, water molecules were excluded from the preprocessing and docking procedures. In the first step, preoptimized 3D conformations (organized second structures) of the target proteins were constructed using the selected templates, then the optimal position of amino acid residues was found by an annealing procedure using different temperature factors thereby achieving the stable state with an insignificant stress influence (Figure S3). As clearly shown in Figure S3, a relatively low stress was achieved for *h*COX-1 and *h*COX-2, except for a minor set of amino acids that are beyond the predefined binding site. The main focus during annealing was placed on amino acids that organized the binding site and neighboring area (30Å) as well as hydrogen atoms therein. As a result, minimized 3D conformations of the target proteins were obtained and subsequently used for modeling. It should be noted that the binding sites of celecoxib in the template proteins and human isoforms are very similar. 

**Figure 7:**
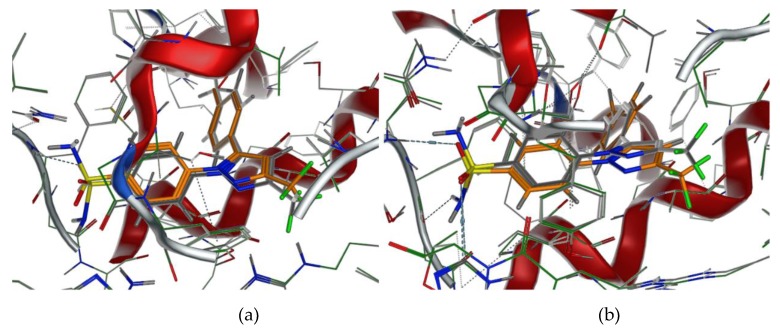
Internal validation of the developed model. The most adequate conformation generated for celecoxib (gray) as compared to the template location (RSA data, orange): *h*COX-2: Free binding energy: −14.27 kcal/mol (**a**); *h*COX-1: Free binding energy: −13.39 kcal/mol (**b**); RMSD = 0.13 and 0.21, respectively.

The constructed models were evaluated using a set of known selective COX-1/2 inhibitors (Figure S1). Celecoxib was also docked into the binding site (Figure 7) to obtain a reference score value (baseline point). During the evaluation, 50 different “active” conformations were generated per ligand. Docking was performed in dynamic mode with the flexible binding site. As shown in Figure 7, the docking results correlated well with the RSA data.

**Figure 8:**
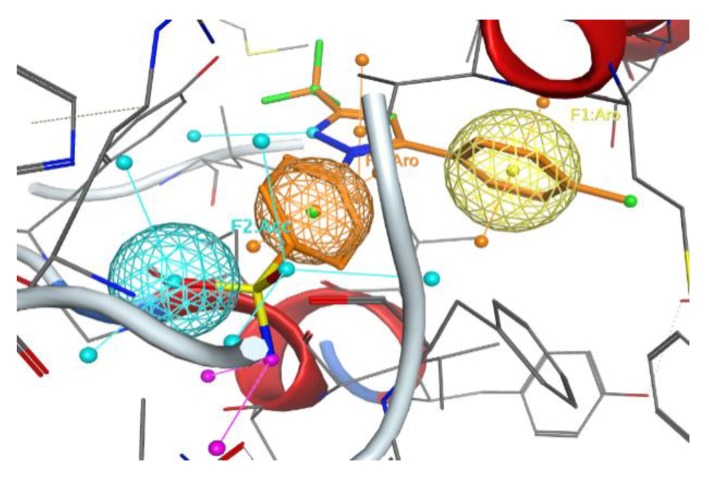
The common 3-centered pharmacophore model for COX-1/2 inhibitors.

The developed model was used to predict the activity of fourteen compounds E1–E14(Figure S1) with known inhibitory action which were used as reference compounds (test set, Figure S3) Docking was carried out in dynamic fashion with the integrated 3-centered pharmacophore model (Figure 8) to restrict the pool of possible conformations. This pharmacophore hypothesis was generated based on the binding mode which was revealed for the template molecule (celecoxib) retaining all the crucial binding points. 

#### 3.1.2. Docking Analysis Based on Ovine COX-1 Structure 2AYL and Human COX-2 Structure 5IKT

Since human COX-2 recombinant enzyme and ovine COX-1 isoenzyme were used for the in vitro evaluation of inhibitory action, a second approach of docking analysis was performed using the human COX-2 structure 5IKT, in complex with tolfenamic acid and several ovine COX-1 structuresamong which was the structure 4O1Z in complex with meloxicam. 

In all cases the target box was set around the ligand. For the human COX-2 structure, 5IKT, the target center was at x = 165.42, y = 185.73, z = 192.38, and target box dimensions were set at 30 × 30 × 30 (X × Y × Z). 

Docking analysis was carried out using Molecular Docking Server [56] and AutoDock tools [57]. 

**Figure 9:**
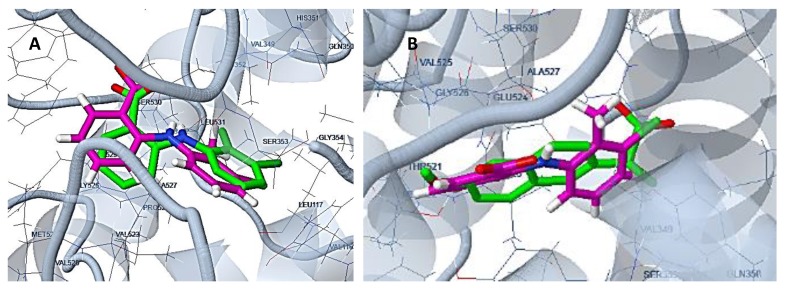
Docking of tolfenamic acid (**A**) and naproxen (**B**) to the structure 5IKT of human COX-2, Energy: −7.04 kcal/mole (docked molecule in green, initial molecule in magenta).

**Figure 10:**
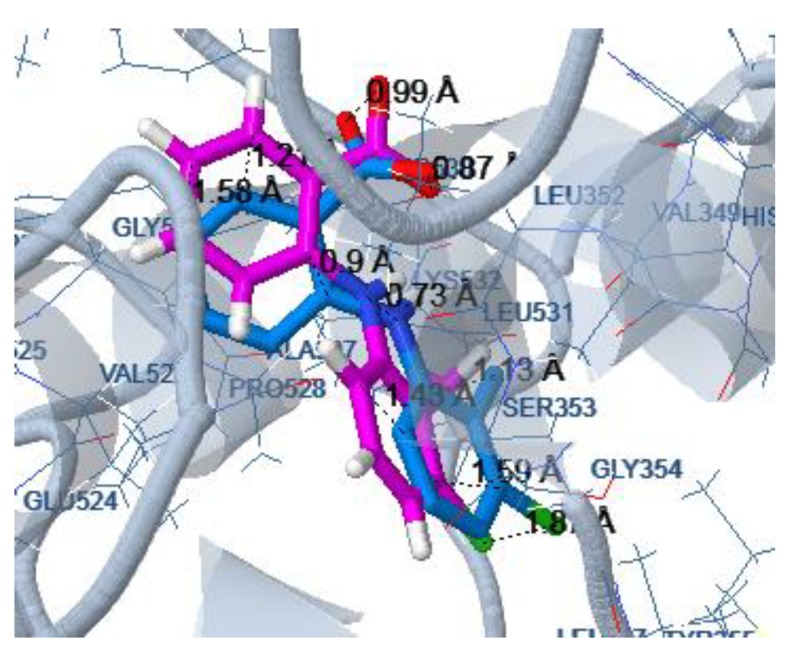
Docking of tolfenamic acid to the structure 5IKT of human COX-2 (docked molecule in blue, initial molecule in magenta), the distances between the same atoms of the two structures do not exceed 1.6 Å.

For the calculation of the van der Waals and the electrostatic terms AutoDock parameter set and distance-dependent dielectric functions were used. The Lamarckian genetic algorithm (LGA) and the Solis & Wets local search method [58] were used to perform docking simulation. More details are given in our previous paper [59]. Docking of the initial ligand was performed for verification of the Docking efficacy (Figure 9A and Figure 10). Docking of known inhibitors was also performed for comparison reasons (Figure 9B).

### 3.2. Chemistry

All solvent used for reactions were dried and purified before by known methods. TLC on precoated silica gel SIL G/UV_254_ plates (Macherey-Nagel & Co. Düren, Germany)) were used for the reaction monitoring. All apparatus used for the compound’s characterization were mentioned in our previous paper [41] 

#### 3.2.1. General Method for Preparation of Compounds **1b**, **1g**, **1j**, and **1n**

To a solution of 2,2-dimethyl-4-[4′-(methylthio)phenyl]-5-phenylfuran-3(*2H*)-one (300 mg, 0.96 mmol) in acetic acid (10 mL, 50–55 °C) a mixture of 35% hydrogen peroxide (100 mg, 0.96 mmol), 98% H_2_SO_4_ (0.05 mL) and acetic acid (3 mL) was added to the reaction dropwise with vigorous stirring for 1 h. The reaction mixture was stirred for an additional 3 h at 50–55 °C (control by TLC). After completion of the reaction, the mixture was poured into water, extracted with ethyl acetate (4 × 20 mL), organic phase was washed by NaHCO_3_/water (3 × 5 mL) solution, dried over Na_2_SO_4_ and K_2_CO_3_, and, after removing the solvent, the residue was dried in vacuum for 24 h. For isolation and purification of compounds the mixtures of n-hexane/CH_2_Cl_2_/ethyl acetate (from 10:2:1 to 2:1:1) were used.

*2,2-Dimethyl-4-[4′-(methylsulfinyl)phenyl]-5-phenylfuran-3(2H)-one* (**1b** racemic mixture) Yield: 270 mg 86% colorless oily substance.^1^H NMR (400 MHz, 20 mg in 0.8 mL of CDCl_3_, reference: CHCl_3_ = 7.260 ppm), δ, ppm: 1.58 (s, 6H, 2CH_3_), 2.74 (s, 3H, SOCH_3_), 7.38 (t, 2H, ^3^*J*_HH_ = 7.8 Hz, Ph), 7.49–7.51 (m, 3H, Ar), 7.63 (t, 4H, *^3^J_HH_* = 7.3 Hz, Ar). ^13^CNMR (100 MHz, 20 mg in 0.8 mL of CDCl_3_, reference: CHCl_3_ = 77.00 ppm), δ, ppm: 23.3 (CH_3_), 23.4 (CH_3_), 43.9 (SOCH_3_), 87.6 (CH_3_)_2_), 112.4 (C_4_), 123.9, 127.9 (2C), 128.5 (2C), 128.7(2C), 129.5, 130.3(2C), 132.2, 144.6 (all C_ArAr’_), 179.2 (C_5_), 204.9 (C=O). IR (in CCl_4_), cm^−1^: 955 w., 1051 m., 1090 w., 1383 s., 1616 m., 1701 s. (C=O), 2931 w., 2982 w. HRMS, *m*/*z*, calculated for C_19_H_18_NaO_3_S 349.0869, found: 349.0876 [M + Na]^+^.

*5-(4′-Fluorophenyl) 2,2-dimethyl-4-[4-(methylsulfinyl)phenyl]-furan-3(2H)-one* (**1g** racemic mixture) Yield: 27%. Slightly yellow oily substance. ^1^H NMR (400 MHz, 25 mg in 0.8 mL of CDCl_3_, reference: CHCl_3_ = 7.26 ppm), δ, ppm: 1.54 (s, 6H, 2CH_3_), 2.72 (s, 3H, SOCH_3_), 7.04 (t, 2H, ^3^*J*_HH_ = 8.7 Hz, Ar), 7.45–7.47 (m, 2H, Ar), 7.60–7.65 (dd, 4H, ^3^*J*_HH_ = 8.7 Hz, ^4^*J*_HF_ = 5.2 Hz). ^13^C NMR (100 MHz, 25 mg in 0.7 mL of acetone-d_6_, reference: CHCl_3_ = 29.84 ppm), δ, ppm: 23.4 (2CH_3_), 43.9 (SOCH_3_), 87.9 (H_3_)_2_), 116.7 (d, 2C, ^2^*J*_CF_ = 22.3 Hz), 124.8 (2C), 127.1 (d, 1C, ^4^*J*_CF_ = 3.2 Hz), 129.0, 131.0 (2C), 131.8 (d, 2C, ^3^*J*_CF_ = 9.1 Hz), 134.0, 145.9, 165.5 (d, 1C, ^1^*J*_CF_ = 252.0 Hz), 178.3 (C_5_), 204.5 (C=O). ^19^F NMR (376 MHz, CDCl_3_) δ, ppm: −105.4. IR (in CCl_4_), cm^−1^: 842 m, 902 w, 1049 m (S=O), 1161 m, 1242 s., 1381 s, 1515 m, 1616 s, 1703 s (C=O), 2980 w. (C-H). LCMS, *m*/*z*, calculated for C_19_H_18_FO_3_S^+^: 345.0956, found 345.1015 [M + H]^+^.

*5-(4′-Chlorophenyl)-2,2-dimethyl-4-[4-methylsulfinyl)phenyl]-furan-3(2H)-one* (**1j** racemic mixture) Yiled: 8%_._ Slightly yellow oily substance. ^1^H NMR (400 MHz, 25 mg in 0.8 mL of CDCl_3_, reference: CHCl_3_ = 7.26 ppm), δ, ppm: 1.54 (s, 6H, 2CH_3_), 2.74 (s,3H, SOCH_3_), 7.32(d, 2H, ^3^*J*_HH_ = 7.4 Hz, PhCl), 7.45 (d, 2H, ^3^*J*_HH_ = 7.5 Hz, PhCl), 7.58 (d, 2H, ^3^*J*_HH_ = 7.4 Hz, PhSOMe), 7.73 (d, 2H, ^3^*J*_HH_ = 7.5 Hz, PhSOMe). ^13^C NMR (100 MHz, CDCl_3_, reference77.00 ppm), δ, ppm: 23.2 (2CH_3_), 43.6 (SOCH_3_), 88.0 (CH_3_)_2_), 112.7 (C_4_), 125.2 (2C), 127.3 (2C), 128.4, 128.7 (2C), 129.0 (2C), 133.2, 134.5, 144.2, 177.5 (C_5_), 204.6 (C=O). LCMS, *m*/*z* calculated for C_19_H_18_ClO_3_S^+^: 361.0660, found 361.0740 [M + H]^+^. 

*5-(3′-Chlorophenyl)-2,2-dimethyl-4-[4′-(methylsulfinyl)phenyl]-furan-3(2H)-one* (**1n** racemic mixture) Yield: 33%. Slightly yellow oily substance. ^1^H NMR (400 MHz, 25 mg in 0.8 mL of CDCl_3_, reference: CHCl_3_ = 7.26 ppm), δ, ppm: 1.53 (s, 6H, 2CH_3_), 2.79 (s, 3H, SOCH_3_), 7.23–7.27 (m, 1H, PhCl), 7.37–7.50 (m, 4H, Ar), 7.60–7.70 (m, 3H, Ar). ^13^C NMR (100 MHz, 20 mg in 0.7 mL of acetone-d_6_, reference: 29.84 ppm), δ, ppm: 23.3 (2CH_3_), 44.2 (SOCH_3_), 88.2 (CH_3_)_2_), 114.1 (C_4_), 124.6 (2C), 127.8, 128.7, 131.0 (2C), 131.3, 132.6, 132.8, 133.5, 135.0, 147.1, 177.6 (C_5_), 204.7 (C=O). IR (in CCl_4_), cm^-1^: 1051 m. (S=O), 1145 m, 1238 w, 1384 s, 1618 m, 1706 s, (C=O), 2983 w (C-H). LCMS, *m*/*z*, calculated for C_19_H_18_ClO_3_S^+^: 361.0660, found 361.0740 [M + H]^+^. 

#### 3.2.2. General Method for Preparation of Compounds **1c**,**1h**, **1k**, and **1o**

To a solution of 2,2-dimethyl-4-[4′-(methylthio)phenyl]-5-phenylfuran-3(*2H*)-one (300 mg, 0.96 mmol) in acetic acid (10 mL, 50–55 °C) a mixture of 35% hydrogen peroxide (210 mg, 1.96 mmol), 98% H_2_SO_4_ (0.05 mL), and acetic acid (3 mL) was added to the reaction dropwise with vigorous stirring for 1 h. The reaction mixture was stirred for 30 minutes at 75–85 °C (control by TLC). After completion of the reaction, the mixture was poured into water, extracted with ethyl acetate (4 × 20 mL), organic phase was washed by NaHCO_3_/water (3 × 5 mL solution, dried over Na_2_SO_4_ and K_2_CO_3_, and, after removing the solvent, the residue was dried under vacuum for 24 h. For isolation and purification of compounds the mixtures of n-hexane/ ethyl acetate (from 20:1 to 1:1) were used. 

*2,2-Dimethyl-4-[4′-(methylsulfonyl)phenyl]-5-phenylfuran-3(2H)-one* (**1c**) Yield: 94%. ^1^H NMR (400 MHz, 25 mg in 0.8 mL of CDCl_3_, reference: CHCl_3_ = 7.260 ppm), δ, ppm: 1.58 (s, 6H, 2CH_3_), 3.04 (s, 3H, SO_2_CH_3_), 7.38 (t, 2H, ^3^*J*_HH_ = 7.7 Hz, Ph), 7.50–7.54 (m, 3H, Ph), 7.61 (d, 2H, ^3^*J*_HH_ = 8.5 Hz, PhSO_2_Me), 7.91 (d, 2H, ^3^J_HH_ = 8.4 Hz, PhSO_2_Me). ^13^C NMR (100 MHz, 25 mg in 0.8 mL of CDCl_3_, reference: CHCl_3_ = 77.00 ppm), δ, ppm: 23.3 (2CH_3_), 43.5 (SO_2_CH_3_), 87.9 (CH_3_)_2_), 111.6 (C_4_), 127.5, 128.4, 128.6, 129.2, 130.1, 132.4, 136.1, 139.0 (all C_ArAr’_), 179.8 (C_5_), 204.4 (C=O). IR (in CCl_4_), cm^−1^: 955 m, 1051 m., 1157 s, 1327 m., 1383 m., 1614 m., 1701 s. (C=O), 2932 w., 2982 w. HRMS, *m*/*z*, calculated for C_19_H_18_NaO_4_S^+^: 365.0818, found 365.0823 [M + Na]^+^.

*5-(3′-Chlorophenyl)-2,2-dimethyl-4-[4′-(methylsulfonyl)phenyl]-furan-3(2H)-one (**1o**)* Yield: 33%. Colorless crystals, m.p. 219–221 °C. ^1^H NMR (400 MHz, 20 mg in 0.8 mL of CDCl_3_, reference: CHCl_3_ = 7.26 ppm), δ, ppm: 1.56 (s, 6H, 2CH_3_), 3.04 (s, 3H, SO_2_CH_3_), 7.29 (d, 1H, ^3^*J*_HH_ = 7.8 Hz, PhCl), 7.38 (d, 1H, ^3^*J*_HH_ = 7.9 Hz, PhCl), 7.47 (d, 1H, ^3^*J*_HH_ = 8.0 Hz, PhCl), 7.51 (d, 2H, ^3^*J*_HH_ = 8.4 Hz, PhSO_2_Me), 7.67 (s,1H, PhCl), 7.91 (d, 2H, ^3^*J*_HH_ = 8.4 Hz, PhSO_2_Me). ^13^C NMR (100 MHz, 25 mg in 0.8 mL of CDCl_3_, reference: CHCl_3_ = 77.00 ppm), δ, ppm: 23.2 (2CH_3_), 44.4 (SO_2_CH_3_), 88.1 (CH_3_)_2_), 112.4, 126.7, 127.6 (2C), 128.0, 130.0, 130.1 (2C), 131.0, 132.3, 135.0, 135.6, 139.3, 177.8 (C_5_), 204.2 (C=O). IR (in CCl_4_), cm^−1^: 957 m., 1157 s. (SO_2_), 1330 s. (SO_2_), 1384 m., 1616 m., 1705 s. (C=O), 2931 w., 2982 w. (C-H). LCMS, *m*/*z*, calculated for C_19_H_18_ClO_4_S^+^: 377.0609, found 377.0667 [M + H]^+^. 

*5-(4′-Chlorophenyl)-2,2-dimethyl-4-[4′-(methylsulfonyl)phenyl]-furan-3(2H)-one (***1k**) Yield: 9%. Colorless crystals, m.p. 223–225 °C. ^1^H NMR (400 MHz, 20 mg in 0.8 mL of CDCl_3_, reference: CHCl_3_ = 7.26 ppm), δ, ppm: 1.57 (s, 6H, 2CH_3_), 3.06 (s, 3H, SO_2_CH_3_), 7.37 (d, 2H, ^3^*J*_HH_ = 8.8 Hz, PhCl), 7.51-7.56 (m, 4H, Ar), 7.53 (dd, 4H, ^3^*J*_HH_ = 8.8 Hz, ^4^*J*_HH_ = 5.4 Hz), 7.92 (d, 2H, *J*= 8.8 Hz, PhSO_2_Me). ^13^C NMR (100 MHz, 25 mg in 0.8 mL CDCl_3_, reference: CHCl_3_ = 77.00 ppm), δ, ppm: 23.3 (2CH_3_), 44.5 (SO_2_CH_3_), 88.1 (CH_3_)_2_), 112.0, 127.7 (2C), 129.2 (2C), 129.7 (2C), 130.2 (2C), 130.6, 135.9, 138.8, 139.3, 178.3 (C_5_), 204.2 (C=O). IR (in CCl_4_), cm^−1^: 956 m., 1095 m., 1158 s. (SO_2_), 1329 s. (SO_2_), 1384 s., 1616 m., 1703 s. (C=O), 2931 w., 2982 w.(C-H). LCMS, *m*/*z* calculated for C_19_H_18_ClO_4_S^+^: 377.0609, found 377.0679 [M + H]^+^.

*5-(4′-Fluorophenyl)-2,2-dimethyl-4-[4′-(methylsulfonyl)phenyl]-furan-3(2H)-one* (**1h**) Yield: 23%. Colorless crystals, m.p. 191–192 °C. ^1^H NMR (400 MHz, 25 mg in 0.8 mL of CDCl_3_, reference: CHCl_3_ = 7.26 ppm), δ, ppm: 1.57 (s, 6H, 2CH_3_), 3.06 (s, 3H, SO_2_CH_3_), 7.09 (t, 2H, *^3^J_HH_* = 8.5 Hz, PhF), 7.52 (d, 2H, *^3^J_HH_* = 8.0 Hz, PhSO_2_Me), 7.63 (dd, 2H, *^3^J_HH_* = 8.8 Hz, ^4^*J*_HF_ = 5.4 Hz, PhF), 7.92 (d, 2H, *^3^J_HH_* = 8.0 Hz, PhSO_2_Me). ^13^C NMR (100 MHz, 25 mg in 0.8 mL of CDCl_3_, reference: CHCl_3_ = 77.00 ppm), δ, ppm: 23.3 (2CH_3_), 44.5 (SO_2_CH_3_), 88.0 (CH_3_)_2_), 116.2 d (2C, ^2^*J*_CF_ = 22.0 Hz), 126.3 (1C), 127.8 (2C), 130.2 (2C), 130.9 d (2C, ^3^*J*_CF_ = 9.0 Hz), 136.1, 139.3, 165.1 d (1C, ^1^*J*_CF_ = 255.4 Hz), 178.4 (C_5_), 204.2 (C=O). ^19^F NMR (376 MHz, CDCl_3_) δ, ppm: -104.8. IR (in CCl_4_), cm^−1^: 955 m., 1157 s. (SO_2_) 1243 m., 1327 s. (SO_2_), 1383 m., 1613 m., 1704 s. (C=O), 2933 w. (C-H). LCMS, *m*/*z*, calculated for C_19_H_18_FO_4_S^+^: 361.0905, found 361.0953 [M + H]^+^.

#### 3.2.3. General Method for Preparation of Compounds **1q**, **1v**, and **1w**

To an aqueous solution of 25% NH_3_ (5mL) 2,2-dimethyl-4-[4′-(chlorosulfonyl)phenyl]-5-(3′-chlorophenyl)furan-3(2H)-one (200 mg) in THF (5 mL) was added and mixture was stirred at room temperature for 24 h. Then, water, ammonia and tetrahydrofuran were removed in vacuo on a rotary evaporator, the solid residue was dissolved in ethanol, then silica gel (5 g) was added, and ethanol was removed in vacuo. The silica gel with the product was washed on a Schott filter 3 times with dichloromethane (5 mL each), after which the compound was washed off the filter by ethanol after removing the alcohol and vacuum drying (0.1 torr, 24 h, 50 °C). For isolation and purification of compounds the mixtures of n-hexane/ CH_2_Cl_2_/ethyl acetate (from 10:2:1 to pure ethyl acetate) were applied.

*5-(3′-Chlorophenyl)-2,2-dimethyl-4-[4′-(aminosulfonyl)phenyl]-furan-3(2H)-one* (**1u**) Yield: 150 mg, 79%. Colorless crystals, m.p. 198–200 °C. ^1^H NMR (300 MHz, 15 mg in 0.6 mL of (CD_3_)_2_CO, reference: 2.05 ppm), δ, ppm: 1.54 (s, 6H, 2CH_3_), 6.63 (s, 2H, NH_2_), 7.45–7.62 (m, 3H, PhCl), 7.49 (d, 2H, *^3^J_HH_* = 8.4 Hz, PhSO_2_Me), 7.68–7.71 (m, 1H, PhCl), 7.89 (d, 2H, *^3^J_HH_* = 8.4 Hz, PhSO_2_Me). ^13^CNMR (75 MHz, 15 mg in 0.6 mL of (CD_3_)_2_CO, reference: 206.26 ppm), δ, ppm: 204.54, 177.90, 144.07, 135.09, 134.77, 132.77, 132.70, 131.37, 130.61, 128.67, 127.86, 127.07, 113.86, 88.33, 23.30. HRMS, *m*/*z*, calculated for C_18_H_17_ClNO_4_S^+^: 378.0562, found: 378.0563 [M + H]^+^.

*5-(4′-Phenyl)-2,2-dimethyl-4-[4′-(aminosulfonyl)phenyl]-furan-3(2H)-one* (**1q**) Yield: 23%.Colorless crystals, m.p. 158–160 °C. ^1^H NMR (400 MHz, 5 mg in 0.7 mL of acetone-d_6_, reference 2.05 ppm), δ, ppm: 1.53 (s, 6H, 2CH_3_), 6.60 (s, 2H, -NH_2_), 7.44-7.49 (m, 4H, Ph), 7.58 (t,1H, ^3^*J*_HH_ = 7.4 Hz, Ph), 7.65 (d, 2H, *^3^J_HH_* = 7.2 Hz, PhSO_2_Me), 7.87 (d, 2H, ^3^*J*_HH_ = 8.5 Hz, PhSO_2_Me). ^13^CNMR (100 MHz, 15 mg in 0.7 mL of acetone-d_6_, reference: 29.84 ppm), δ, ppm: 23.4 (2C), 88.0 (C_2_), 113.0 (C_4_), 127.0 (2C), 129.2 (2C), 129.6 (2C), 130.6 (2C), 130.7, 133.0, 135.3, 143.7, 179.7 (C_5_), 204.5 (C=O). IR (in KBr), cm^−1^: 3346 br. (-SO_2_NH_2_), 3234 br. (-SO_2_NH_2_), 2988 w. (CH_3_, Ar), 2938 w. (CH_3_, Ar), 1662 v.s., 1607 s., 1568 s., 1485 m., 1386 s., 1342 s., 1238 m., 1170 s., 1058 m., 904 m., 746 m., 658 m., 597 m., 553 m. HRMS, *m*/*z*, calculated for C_18_H_18_NO_4_S^+^: 344.0952, found: 344.1006 [M + H]^+^. 

*5-(4′-Chlorophenyl)-2,2-dimethyl-4-[4′-(aminosulfonyl)phenyl]-furan-3(2H)-one* (**1v**) Yield 29%. Colorless crystals, m.p. 224–225 °C. ^1^H NMR (400 MHz, 5 mg in 0.7 mL of acetone-d_6_, reference: 2.05 ppm), δ, ppm: 1.53 (s, 6H, 2CH_3_), 4.82 (s, 2H, -NH_2_), 7.44-7.49 (m, 4H, Ar), 7.64 (d, 2H, ^3^*J*_HH_ = 7.2 Hz, Ar), 7.86 (d,2H, ^3^*J*_HH_ = 8.5 Hz, PhSO_2_Me). ^13^C NMR (75 MHz, 10 mg in 0.6 mL ofCDCl_3_, reference: 77.00 ppm), δ, ppm: 204.51, 178.16, 140.80, 138.76, 134.76, 129.98 (2C), 129.76, 129.14 (2C), 127.68 (2C), 126.85 (2C), 112.16, 88.03, 23.31. HRMS, *m*/*z*, calculated for C_18_H_17_ClNO_4_S^+^: 378.0562, found: 378.0578 [M + H]^+^. 

*5-(4′-Fluorophenyl)-2,2-dimethyl-4-[4′-(aminosulfonyl)phenyl]-furan-3(2H)-one* (**1w**) Yield: 36%. Colorless crystals, m.p. 167–169 °C. ^1^H NMR (300 MHz, 10 mg in 0.6 mL of CDCl_3_, reference: 7.26 ppm), δ, ppm: 1.55 (s, 6H, 2CH_3_), 5.42 (s, 2H, NH_2_), 7.06 (t, 2H, *^3^J_HH_* = 11.4 Hz, PhF), 7.41 (d, 2H, ^3^*J*_HH_ = 8.9 Hz, PhSO_2_NH_2_), 7.60–7.65 (m, 2H, PhF)), 7.86 (d, 2H, *J* = 11.0 Hz, PhSO_2_NH_2_). ^13^CNMR (75 MHz, 10 mg in 0.6 mL of CDCl_3_, reference: 77.00 ppm), δ, ppm: 204.72, 178.32, 164.96 d (*J* = 255.2 Hz), 141.07, 134.64, 130.94, 130.83, 129.93, 128.46, 128.32, 126.72, 125.43(d, 2C, ^3^J_HF_ = 3.3 Hz), 116.04 (d, 2C, ^2^*J*_HF_ = 22.0 Hz), 111.80, 87.93, 23.24. HRMS, *m*/*z*, calculated for C_18_H_17_FNO_4_S^+^:362.0857, found: 362.0861 [M + H]^+^.

#### 3.2.4. 5-(4′-Chlorophenyl)-2,2-dimethyl-4-[4′-(methylaminosulfonyl)phenyl]-furan-3(2*H*)-one (**1vMe**)

A mixture (450 mg) of 2,2-dimethyl-4-[4′-(chlorosulfonyl)phenyl]-5-(4′-chlorophenyl)furan-3 (*2H*)-one and 2,2-dimethyl-4-[4′-chlorophenyl]-5-(phenyl)furan-3(*2H*)-one was dissolved in triethylamine (10 mL), presaturated by CH_3_NH_2_, and obtained by heating a 60% solution of methylamine in water and passing gas through a layer of CaCl_2_.The resulting solution was stirred at 45 ° C for 24 h. Then the volatile products were removed on a rotary evaporator. The solid residue was applied to silica gel (3 g) and washed 3 times with a mixture of hexane-methylene chloride (1:1, 10 mL) to remove nonpolar impurities. After that, desired product **1vMe** was washed off from silica gel by ethanol. Finally, 160 mg (67%) of the product 1vMe was obtained after removing the solvent. For the purification of compound the mixtures of n-hexane/ CH_2_Cl_2_/ethanol (from 10:1:0.1 to 1:1:1) were used.

Colorless crystals, m.p. 204–205 °C. ^1^H NMR (300 MHz, 15 mg in 0.6 mL of acetone-d_6_, reference: 2.05 ppm), δ, ppm: 1.52 (s, 6H, 2CH_3_), 2.57 (s, 3H, CH3), 4.82 (s, 1H, NHCH_3_), 7.48-7.52 (m, 4H, Ph) 7.66 (d, 2H, ^3^*J*_HH_ = 8.7 Hz, Ar), 7.83 (d, 2H, ^3^*J*_HH_ = 8.0 Hz, PhSO_2_NHCH_3_). ESMS, calculated for C_19_H_19_ClNO_4_S^+^: *m*/*z* = 392.0718, found: 392.0745.

#### 3.2.5. General Method for the Synthesis of Phenanthro[9,10-b]furan-3′(2′H)-ones

A solution of 2,2-dimethyl-4-[4′-(methylthio)phenyl]-5-phenylfuran-3(*2H*)-one (500 mg, 1.61 mmol) in dichloromethane (5 mL) placed in a quartz photochemical reactor with water cooling and a volume of 50 mL and irradiated with a mercury UV lamp of 150 W during 4 h in the presence of oxygen. After completion of the reaction, the solvent was distilled off on a rotary evaporator, the mixture was applied onto a preparative chromatography plate and divided into fractions (eluent: hexane–ethyl acetate–dichloromethane). The yield of 2′,2′-dimethyl-6-(methylthio)phenanthro[9,10-b]furan-3′(2′H)-one was 70 mg (14%), colorless crystals, violet fluorescence (254 nm). The degree of conversion of the starting 3(*2H*)furanone was 90%.

##### 2′,2′-dimethyl-3-(methylsulfonyl)-phenanthro[9,10-b]furan-3′(2′H)-one (**X-1**)

2′,2′-Dimethyl-6-(methylsulfonyl)-phenanthro[9,10-b]furan-3′(*2′H*)-one may be prepared by oxidation of corresponding sulfide as described above. Yield: 6%. Colorless crystals, m.p. 222–224 °C. ^1^H NMR (300 MHz, 5 mg in 0.8 mL of CDCl_3_, reference: CHCl_3_ = 7.26 ppm), δ, ppm: 1.68 (s, 6H, 2CH_3_), 3.06 (s, 3H, SO_2_CH_3_), 7.72 (td, 1H, ^3^*J*_HH_ = 7.4, 1.6 Hz), 7.80 (td, 1H, ^3^*J*_HH_ = 7.5, *^4^J*_HH_ = 1.5 Hz), 8.10 (dd, 1H, ^3^*J*_HH_ = 7.5 Hz, ^4^J_HH_ = 1.5 Hz), 8.32 (dd, 1H, ^3^*J*_HH_ = 7.4, ^3^*J*_HH_ = 5.1 Hz), 8.50 (d, 1H, *J* = 8.0 Hz), 8.79 (dd,1H, *^3^J*_HH_ = 7.5 Hz, ^4^*J*_HH_ =1.5 Hz), 9.30 (d, 1H, ^4^*J*_HH_ = 1.6 Hz). LCMS, *m*/*z*, calculated for C_19_H_18_O_4_S^+^: 342.0843, found: 342.0826 [M + H]^+^.

##### Synthesis of Intermediate 2′,2′-dimethyl-6-(methylthio)-phenanthro[9,10-b]furan-3′(*2′H*)-one

Slightly yellow crystals, m.p. 178–180 °C. ^1^H NMR (400 MHz, 25 mg in 0.8 mL CDCl_3_, reference: CHCl_3_ = 7.260 ppm), δ, ppm: 1.62 (s, 6H, 2CH_3_), 2.64 (s, 3H, SCH_3_), 7.60 (dd, 1H, ^3^*J*_HH_ = 8.5 Hz, ^4^*J*_HH_ = 1.8 Hz), 7.70 (t, 1H, ^3^*J*_HH_ = 7.3 Hz), 7.90–7.84 (m, 1H), 8.33 (dd, 1H, ^3^*J*_HH_ = 8.0 Hz, ^4^*J*_HH_ = 1.0 Hz), 8.45 (d,1H, ^4^*J*_HH_ = 1.5 Hz), 8.63 (d,1H, ^3^*J*_HH_ = 8.4 Hz), 8.76 (d,1H, ^3^*J*_HH_ = 8.4 Hz). LCMS, *m*/*z*, calculated for: C_19_H_16_NaO_2_S^+^: 331.0764, found: 331.0769 [M + Na]^+^.

##### 3-Fluoro-2,2-dimethyl-6-(methylsulfonyl)phenanthro[9,10-b]furan-3(2′H)-one (**X-2**)

3-Fluoro-2′, 2′-dimethyl-6-(methylsulfonyl)-phenanthro[9,10-b]furan-3′(*2′H*)-one (**X-2**) may be prepared by oxidation of corresponding sulfide as described above.

Here we present synthesis of intermediate 3-fluoro-2′, 2′-dimethyl-6-(methylthio)-phenanthro-[9,10-b]furan-3′(*2′H*)-one.

The first step: synthesis of 3-fluoro-2′,2′-dimethyl-6-(methylthio)-phenanthro[9,10-b]furan-3′(2′H)-one.

5-(4′-fluorophenyl)-2,2-dimethyl-4-[4′-(methylthio)phenyl]-)furan-3(*2H*)-one (**3**, **X**-2) (305 mg) dissolved in boiling *n*-hexane (30 mL) was placed in a quartz reactor and irradiated by a low-pressure UV mercury lamp (240 W, λ > 240 nm) during 2 h. After chromatography 27 mg (9%) of the product was obtained. The conversion rate was 34% and 200 mg of starting 3(*2H*)furanone was isolated. 

Slightly yellow oily substance. ^1^H NMR (300 MHz, 5 mg in 0.8 mL of CDCl_3_, reference: CHCl_3_ = 7.26 ppm), δ, ppm: 1.65 (s, 6H, 2CH_3_), 2.64 (s, 3H, SCH_3_), 7.48 (td, 1H, ^3^J_HH_ = 7.9 Hz, ^4^*J*_HH_ = 1.4 Hz), 7.66 (dd, 1H, ^3^*J*_HH_ = 7.4 Hz, ^4^*J*_HH_ = 1.5 Hz), 8.08 (dd, 1H, ^3^*J*_HH_ = 7.5 Hz, ^4^*J*_HF_ = 5.0 Hz), 8.14 (d, 1H, ^3^*J*_HH_ = 7.5 Hz), 8.38 (dd, 1H, ^3^*J*_HH_ = 8.0 Hz, ^4^*J*_HH_ = 1.5 Hz), 8.56 (d, 1H, ^4^*J*_HH_ = 1.6 Hz). LCMS, *m*/*z*, calculated for C_19_H_16_FO_2_S^+^: 327.0850, found: 327.0792 [M + H]^+^.

The second step: Unreacted 5-(4′-fluorophenyl)-2,2-dimethyl-4-[4′-(methylthio)phenyl]-furan-3-(2*H*)-one (**2, X-2**)

(200 mg) dissolved in 25 mL of *n*-hexane and the irradiation procedure was repeated. A unreacted starting 3(*2H*)furanone (143 mg) was isolated and 27 mg (13.5%) of desired phenanthro[9,10-b]furanone was obtained. Total yield: 54 mg (17.7%). 

##### 3-Fluoro-2′,2′-dimethyl-6-(methylsulfonyl)-phenanthro[9,10-b]furan-3′(2′H)-one (**X-2**)

Colorless crystals, m.p. 242–244 °C. ^1^H NMR (300 MHz, 5 mg in 0.8 mL of CDCl_3_, reference: CHCl_3_ = 7.26 ppm), δ, ppm: 1.65 (s, 6H, 2CH_3_), 3.16 (s, 3H, SO_2_CH_3_), 7.50-7.55 (m, 1H), 8.17 (dd, 1H, ^3^*J*_HH_ = 8.6 Hz, ^4^*J*_HH_ = 1.7 Hz), 8.37 (dd, 1H, ^3^*J_H_*_H_ = 10.6 Hz, ^4^*J*_HH_ = 2.3 Hz), 8.43 (dd, 1H, ^3^*J*_HH_ = 8.9 Hz, ^4^*J*_HF_ = 5.8 Hz), 9.02 d (1H, ^4^*J*_HH_ = 1.6 Hz), 9.04 (d, 1H, ^3^*J*_HH_ = 6.3 Hz). LCMS, *m*/*z*, calculated for C_19_H_16_FO_4_S^+^: 359.0748, found: 359.0763 [M + H^+^]. 

### 3.3. In Vitro Evaluation of COX Inhibitory Action

The COX-1 and COX-2 activities of the compounds were measured using ovine COX-1 and human recombinant COX-2 enzymes included in the “COX Inhibitor Screening Assay” kit provided by Cayman (Cayman Chemical Co., Ann Arbor, MI, USA) as reported previously [60,61] 

### 3.4. Inhibition of the Carrageenin-Induced Edema

The anti-inflammatory activity of compounds was performed using the carrageenan mice paw oedema assay. Animal of both sex (AKR), with exception of pregnant females were housed under standard conditions, receiving only water during the experimental period. The compounds (0.01 mmol/kg body weight) were administered intraperitoneally simultaneously with the carrageenin injection. The detailed description of the whole experiment reported previously [61]. All the biological experiments were carried out in full compliance with the European Convention for the Protection of Vertebrate Animals used for Experimental or Other Scientific Purposes (ETS NO 123, Strasbourg, 03/18/1986): Strasbourg (France). European Treaty Series, № 123, March 18, 1986. 11 P. The project identification code and date of approval is 350880/3468 of 15-12-2014, University of Thessaloniki.

### 3.5. Cell Cultures

The antitumor activity of the compounds was tested using two different cancer cell lines, breast cancer cell line, MCF-7, which mainly expresses the COX-1 isoenzyme [43] and the tongue squamous cell carcinoma cell line, HSC3, which is known to express COX-2 isoenzyme, although COX-1 is also produced [16,20].

Malignant MCF-7 cells (human epithelial breast adenocarcinoma cell line) and HSC-3 (human oral squamous cell carcinoma cell line) cells were grown in culture (37 °C; humidified atmosphere containing 5% (*v*/*v*) CO_2_) in Dulbecco’s Modified Eagle’s medium supplemented with 10% (*v*/*v*) fetal bovin serum (FBS), 10,000 units/mL of penicillin, 10,000 μg/mL of streptomycin, and 25 μg/mL of amphotericin B. In order to allow the continuous logarithmic phase of cell growth in culture, cells were split accordingly before reaching 80% confluency (approximately every 48–72 h) with trypsin-EDTA (0.25% *w*/*v*). According to ATCC (American Tissue Culture Collection), the MCF-7 cell express the WNT7B oncogene, the estrogen receptor, and the insulin-like growth factor binding proteins (IGFBP) BP-2, BP-4, and BP-5; addition of 0.01 mg/mL human recombinant insulin in the cell culture medium is proposed. EGF Receptors are expressed when the other growth factors’ pathways are inhibited or after long term culture in the presence of Epidermal Growth Factor [62,63,64]. 

### 3.6. Growth Inhibition Assay

To estimate growth inhibition rate, we tested increasing concentrations (0.01–100 μM) of the compounds exposure to MCF-7 cells after appropriate serial dilutions of every compound stock solution. Each cell culture was treated appropriately, ensuring that DMSO concentration was kept below ≤ 0.2% (*v*/*v*) after every dilution. Cells were seeded in 24-well plates, at an initial density of 5 × 10^4^ cells/mL. Cell cultures were left overnight at 37 °C to ensure appropriate cell attachment, before the compounds’ addition at the specified concentrations. Cells were allowed to grow for additional 48 h before being harvested by trypsinization and subsequently counted (cell density; number of cells/mL) using a hemocytometer (Neubauer counting chamber) and optical microscopy. Cell growth was expressed as a percentage relative to that for the untreated control culture (only cells and medium, CTL). For the combination experiments, a Combination Index was measured using the equation CI = C_A_,x/Cx,_A_ + C_B_,x/Cx,_B_ where C_A_,x and C_B_,x stand for the concentrations of compounds A and B respectively in the combination mixture which results in x% inhibition of cell growth, while Cx,_A_ and Cx,_B_ are the concentrations of compounds A and B, respectively, which alone cause x% inhibition of cell growth. A CI of less than, equal to, and more than 1 indicates synergy, additivity, and antagonism, respectively [65].

## 4. Conclusions

Thirteen new 4,5-diarylfuran- 3(*2H*) ones were synthesized based on molecular docking for COX inhibitory action. Their anti-inflammatory and COX-1/COX-2 inhibitory activities were evaluated in vitro. Cytotoxicity of compounds on MCS-7 and HSC-3 cancer cell lines were performed.

It was found that three compounds inhibited the MCF-7 cell growth with an IC_50_ value of 24.0 µM for **1h** and 10.0 µM for **1g** and **X-1**, while the IC_50_ values of the control compounds for gefitinib, 5-fluorouracil and celecoxib were 70.0 µM, 0.3 µM and 29.2 µM, respectively. Compound **1g** exhibited remarkable growth inhibition of the HSC-3 cells with an IC_50_ value of 7.5 μΜ. 

The most active COX-1 selective inhibitor, **1g**, exhibited synergistic effect with both anti-neoplastic drugs with the best effect when used in combination with 5-fluorouracil (CI = 0.058). Lower synergistic effect with 5-fluorouracil was shown by the less active compound **1h** (CI = 0.533). A synergistic effect with gefitinib was found for compounds **1g** (CI = 0.780) and **X-1** (CI = 0.260), while the low activity compound **1h** exhibited antagonistic effect. 

A synergistic effect of the most active COX-1 inhibitor **1g** with the known COX-2 selective inhibitor, celecoxib, was also observed (CI = 0.75), which may be indicative for the involvement of both cyclooxygenase isoenzymes in cancer cell development and the value of combined COX1/2 inhibition for best inhibitory effect.

## Figures and Tables

**Table 1:** Predicted IC_50_ values of the designed compounds.

			COX-1		COX-2		
Structure	Code	R	Binding Energy (Kcal/mole)	IC_50_ (μΜ)	Binding Energy (Kcal/mole)	IC_50_ (μΜ)	S *
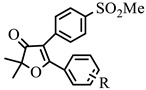	**1c**	H	−5.78	25.8	−6.67	0.39	66.2
	**1o**	*m*–Cl	−6.18	17.0	−10.21	0.15	113.3
	**1h**	*p*–F	−5.27	37.0	−7.17	0.36	102.8
	**1k**	*p*–Cl	−4.96	43.8	−7.32	0.35	125.1
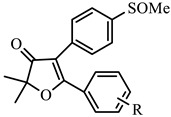	**1b**	H	−5.68	27.0	−6.38	0.41	65.9
	**1n**	*m*–Cl	−5.97	21.6	−7.60	0.33	65.5
	**1g**	*p*–F	−5.94	22.2	−7.61	0.33	67.3
	**1j**	*p*–Cl	−6.44	11.2	−6.93	0.37	30.3
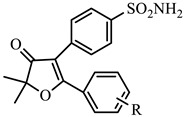	**1q**	H	−6.48	10.3	−9.13	0.22	46.8
	**1v**	*p*–Cl	−6.19	16.7	−9.99	0.16	1–4.4
	**1w**	*p*–F	−5.83	24.7	−9.61	0.19	130.0
**Celecoxib**				14(exp)		0.04(exp)	
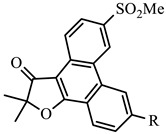	**x-1**	H	−4.92	44.7	−7.28	0.28	159.6
	**x-2**	F	−5.23	37.9	−8.86	0.24	157.9

* selectivity.

**Table 2:** Yields (%) of reactions on different steps of synthesis of sulfoxides and sulfones (Scheme 1).

R	1,4-diol (6)	Dihydro furan-3-one (5)	4-diazodi-hydrofuran-3-one (4)	4,5-diaryl-3(*2H*) furanones (2)	Sulfoxides 1b,n,g,j	Sulfones 1c,o,h,k
*m*-Cl	57 (65) ^a^	90	83-87	83% (94; 5.2:1) ^c^	92%	93%
*m*-F	25 (33) ^a^	31	52 (71) ^b^	66% (75; 7.3:1) ^c^	-	-
*p*-Cl	23 (28) ^a^	91	70	65% (86; 3.2:1) ^c^	89%	98%
*p*-F	52 (64) ^a^	84	86	77% (99; 3.5:1) ^c^	94%	80%
H	86–93	93	71	75% (99; 3.2:1) ^c^	84%	96%

^a^ The yield in brackets is given taking into account incomplete conversion of benzophenone. ^b^ The yield in brackets is given taking into account incomplete conversion of furanone. ^c^ In brackets the yield of both products (stage 4) and their ratio are given.

**Table 3:** Yields (%) of reactions on different steps of sulfonyl amide synthesis (Scheme 2).

R	1,4-diol (6)	dihydrofuran-3-one (5)	4-diazodihyd-rofuran-3-one (4)	4,5-diaryl-3(*2H*) furanones (3)	Sulfonyl Chloride	Sulfonyl amide (1q,1u, 1w,1v)
*m*-Cl	95	98	85	67 (95;2.45:1) ^a^	45 ^b^	79
*p*-Cl	96	98	87	55 (99; 1.25:1) ^a^	50 ^b^	71
*p*-F	98	96	86	47 (99; 1:1.12) ^a^	62 ^b^	72
H	90	95	69	99 (one product)	50	74

^a^ In brackets, the yield of both 1,2-migration products and their ratio are given. ^b^ As a percentage of the total weight of the mixture **3**.

**Table 4:** Total yield (%) of desired 4,5-diarylfuran-3(*2H*)-ones **1**–**2** starting from benzophenones.

R	Sulfones (SO_2_Me) (1c,1o,1h,1k)	Sulfoxides (SOMe) (1b,1n,1g,1j)	Sulfonamides (SO_2_NH_2_) (1q,1u,1w,1v)	Phenanthrenes
*m*-Cl	33	33	27	-
*p*-Cl	9	8	29	-
*p*-F	23	27	36	3.1 **(X-2)**
H	43	37	23	6.0 **(X-1)**

**Table 5:** Inhibition % of COX-1/2 isoenzymes *.

Compound	% Inhibition (COX-1)	% Inhibition (COX-2)
**1c** (H)	0	0
**1h** (*p*–F)	32	0
**1k** (*p*–Cl)	80	6
**1j** (*p*–Cl)	0	0
**1g** (*p*–F)	73	0

* Compound concentration 50 μΜ. Arachidonic acid concentration: 0.1 μM.

**Table 6:** Predicted Energy and IC_50_ values and in vitro activity of synthesized compounds against COX-1/2^1^.

	Experimental IC_50_, μΜ		Docking Assisted Predicted Values					
Comp.			*^2^* *Ovis aries* COX-1, 3KK6		*^2^* *Mus musculus* COX-2, 3LN1		*^3^* Ovine COX-1 4O1Z	*^3^* Human COX-2 5IKT
	COX-1	COX-2	Ε Kcal/mol	IC_50_ (μΜ)	Ε Kcal/mol	IC_50_ (μΜ)	COX-1	COX-2
**1k** (*p*–Cl)	29	> 50	−4.96	43.8	−7.32	0.345	−5.69	+5.91
**1o** (*m*–Cl)	22	71	−6.18	17.0	−10.21	0.147	−6.27	−5.49
**1g (*p*–F)**	**2.8**	> 50	−5.94	22.2	−7.61	0.325	−6.33	−3.50
**1v (*p*–Cl)**	**28**	**20**	−6.19	16.7	−9.99	0.162	−7.01	−4.36
**1w** (*p*–F)	70	>50	−5.83	24.7	−9.61	0.188	−4.85	−2.92
**x-1**							−6.09	−5.87
Naproxen	40	50						−5.66

^1^ Methylsulfides (−SCH_3_) were not tested due to their low solubility in polar solvents like water/dmso mixtures. ^2^ Docking analysis using 3D structures of human isoforms, constructed based on the templates of 3KK6 *Ovis aries* COX-1 and 3LN1 and *Mus musculus* COX-2, in complex with Celecoxib. ^3^ Docking analysis using the human COX-2 structure 5IKT, in complex with tolfenamic acid and ovine COX-1 structure 4O1Z in complex with meloxicam.

**Table 7:**
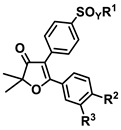
In vivo anti-inflammatory activity of alkylsulfones and sulfoxides of furan-3(*2H*)-ones, including annelated derivatives.

Class	Compound	Dose, µmol/g	CPE, % *
**SO_2_CH_3_**	**1c** (H)	0.1	50.2
	**1o** (*m*–Cl)	0.1	50.7
	**1h** (*p*–F)	0.1	48.8
	**1k** (*p*–Cl)	0.1	50.6
**SOCH_3_**	**1b** (H)	0.1	47
	**1n** (*m*–Cl)	0.1	51
	**1g** (*p*–F)	0.1	49
	**1j** (*p*–Cl)	0.1	54
**SO_2_NH_2_**	**1q** (H)	0.1	38
	**1v** (*p*–Cl)	0.1	44.3
	**1w** (*p*–F)	0.1	43.7
**Phen**	**x-1** (H)	0.1	45
	**x-2** (*p*–F)	0.1	40
**Reference**	**E-1** (H)	0.1	57.7
	**E-3** (*m*–Cl)	0.1	52.1

* Values are the mean of three determinations, and deviation from the mean is <10% of the mean value.

**Table 8:** IC_50_ values of the new synthesized compounds as well as gefitinib, 5-fluorouracil and celecoxib.

Compound	MCF-7 IC_50_ Values (µM) *	HSC-3 % Cell ** Growth	HSC-3 IC_50_ Values (µM) *
**1-h**	24	100 ± 10	90
**1-g**	10	36 ± 4	7.5
**x-1**	10	91 ± 10	60
Gefitinib	70		
5-Fluorouracil	0.03		
Celecoxib	29.186		

* Data are the mean average of 3 independent experiments. ** Compound concentration: 10μΜ.

**Table 9:** % MCF-7 cell growth rates after cell exposure to the combinations of each novel compound **1h, 1g**, and **x-1** with gefitinib, 5-fluorouracil or celecoxib in their IC_50_ or lower concentrations, for 48 h.

Compound * Concentrations (μM)	% Cell Growth	CI	Combination Effect
27.5 μM **1h** + 70 μM gef	45.9	>1	antagonism
10 μM **1g** + 70 μM gef	36.5	0.780 ± 0.200 < 1	synergism
10 μM **x-1** + 70 μM gef	24.3	0.260 ± 0.033 < 1	synergism
29 μM cel + 70 μM gef	56.8	>1	antagonism
21.3 μM **1h** + 0.3 μM 5-fu	27.3	0.533 ± 0.015 <1	synergism
2.9 μM **1g** + 0.3 μM 5-fu	19.1	0.058 ± 0.010 <1	synergism
3.8 μM **x-1** + 0.3 μM 5-fu	21.0	>1	antagonism
29 μM cel + 00.3 μM 5-fu	49.3	>1	antagonism
21.3 μM **1h** + 29 μM cel	38.6	>1	antagonism
2.9 μM **1g** + 29 μM cel	36.6	0.750 ± 0.025 < 1	synergism
3.8 μM **x-1** + 29 μM cel	60.6	>1	antagonism

* Abbreviations: gef: gefitinib; 5-FU: 5-fluorouracil; cel: celecoxib.

## Supplementary Materials

The following are available online, Figure S1. Structures of known COX-1/2 inhibitors (E1–E14). Figure S2. Assessment of cell growth of MCF-7 cells in culture after their treatment with increasing concentrations of the tested compounds for 48 h. Figure S3. Potential energy (NormEnergy, kcal/mol) shared by amino acids in *h*COX-2 (**a**) and *h*COX-1 (**b**) after annealing; (**c**) amino acids in *h*COX-1 with high energy values (*gray spheres*); the binding site (*yellow sphere*). Scheme S1. Synthesis of starting diaryl ketones by oxidation of substituted toluenes or benzaldehydes. For *m*-F-substituted compounds Schiemann reaction is the most rational. Table S1. Yields of the synthesis of starting diaryl ketones and their precursors according to Scheme S1.
