# Supplementary material for: 4,5-Diaryl 3(2H)Furanones: Anti-Inflammatory Activity and Influence on Cancer Growth
Source: Molecules. 2019 May 6;24(9):1751. doi: 10.3390/molecules24091751 (PMC6539231; doi:10.3390/molecules24091751)

SUPPORTING INFORMATION

4,5-Diaryl 3(*2H*)Furanones: Anti-Inflammatory Activity and Influence on Cancer Growth

D. Semenok [1,2], J. Medvedev [3], L-P. Giassafaki [4], J Lavdas [4], I. S. Vizirianakis [4], Phaedra Eleftheriou [5] Antonis Gavalas [6], Anthi Petrou [6] and Athina Geronikaki [6,*]

1 Skolkovo Institute of Science and Technology, Skolkovo Innovation Center 143026, 3 Nobel Street, Moscow, Russia; e-mail@e-mail.com
2 Moscow Institute of Physics and Technology, 141700, 9 Institutsky lane, Dolgoprudny, Russia; e-mail@e-mail.com
3 Saint-Petersburg State University, Institute of Chemistry, Universitetskiy Prospekt, 26, Petergof, Russia, 198504; e-mail@e-mail.com
4 Department of Pharmacology, School of Pharmacy, Aristotle University of Thessaloniki, 54124, Greece; e-mail@e-mail.com
5 Department of Medical Laboratory Studies, School of Health and Medical Care, Alexander Technological Educational Isntitute of Thessaloniki, Greece; e-mail@e-mail.com
6 [6]Department of Pharmaceutical Chemistry, School of Pharmacy, Aristotle University of Thessaloniki, 54124, Greece; e-mail@e-mail.com

Content

1. **Structures of known COX-1/2 inhibitors (E1-E14) used for prediction of inhibitory action (Fig. S1) S2**
2. **Synthesis of auxiliary compounds…(Scheme S1, Table S1) S2-S4**
3. **General procedure for the preparation of compounds 3 S4-S5**
4. **Assessment of cell growth of MCF-7 cells in culture after their treatment with increasing concentrations of the tested compounds for 48 hours.(Fig. S2) S5-S6**
5. **Computer modeling of pharmacological activity (Fig. S3) S7-S8**
6. **Spectra**

1.Structures of known COX-1/2 inhibitors (E1-E14) used for prediction of inhibitory action

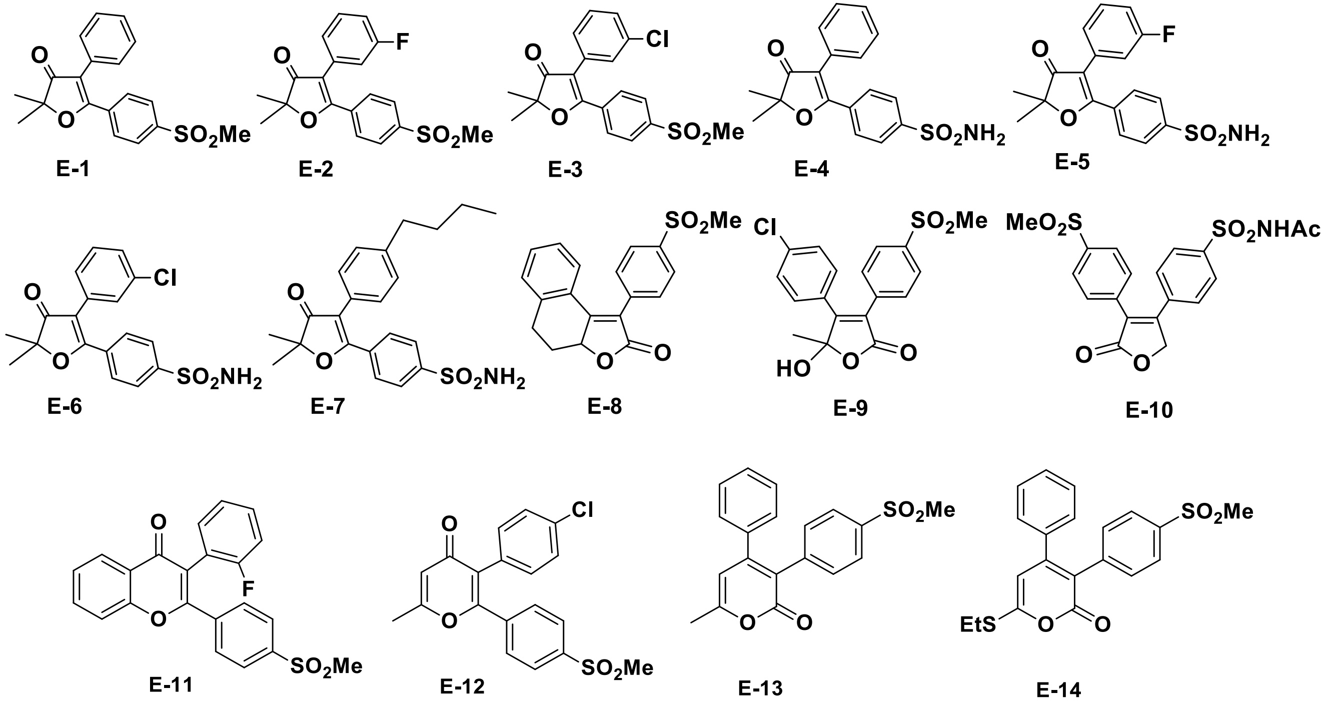

Figure S1. Structures of known COX-1/2 inhibitors (E1-E14)

2. Synthesis of auxiliary compounds

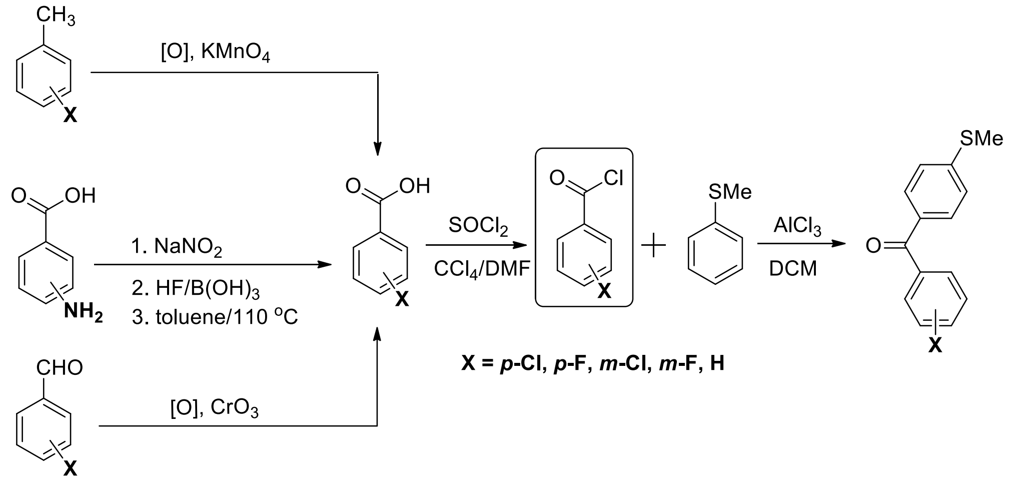

Scheme S1. Synthesis of starting diaryl ketones by oxidation of substituted toluenes or benzaldehydes. For *m*-F substituted compounds Schiemann reaction is the most rational (Table S1)

Table S1. Yields of the synthesis of starting diaryl ketones and their precursors according to Scheme S1.

X	Oxidation of aldehyde	Oxidation of toluene	Schiemann reaction	Acylation by Friedel-Crafts*	Overall yield
m-Cl	-	40 %	-	66 %	26 %
m-F	-	-	35 %	38 %	13 %
p-Cl	66 %	-	-	70 %	46 %
p-F	42 %	-	-	75 %	32 %

* Synthesis of benzoic acid chlorides was carried out without its isolation.

Example: synthesis of 4′-(methylthio)phenyl(4′-fluorophenyl)methanone

To p-fluorobenzene aldehyde (10.7 g) dissolved in acetic acid (60 ml)at 70 °C a solution of CrO_3 (5.5 g) in 70 % acetic acid (60 ml) was added dropwise during 1.5 hours. . After completion of the reaction cold HCl (35 %,100 mL) was added to the mixture. Precipitated *p*-fluorobenzoic acid was filtered, washed twice by 10% HCl, and recrystallized from MeOH/CH_2Cl_2, and the mother liquor was passed through a column of silica gel. Yield after vacuum drying: 4.96 g (42 %). *p*-fluorobenzoic acid, ^{1}H NMR (400 MHz, $CDCl_3$), δ, ppm: 7.154 t (2H, $^3J_{HH}$ = 8.6 Hz), 8.135 dd (2H, $^3J_{HH}$ = 8.7 Hz, $^4J_{HF}$ = 5.4 Hz).

In the next stage to *p*-fluorobenzoic acid (4.96 g) in CCL4 (25ml) fresh $SOCl_2$ (4ml) and DMFA (0.05 ml) in CCl_4 (25 ml)were added. The mixture was refluxed during 5 hours until the end of release of gaseous products. The solvent and excess of $SOCl_2$ were removed in vacuum. Obtained oil (5.6 g ,97 % of theoretical yield) , without additional purification, was dissolved in cold DCM (20 mL). This solution was added dropwise to cold suspension of $AlCl_3$ (5 g) and thioanisole (4.38 g ,35 mmol) in of DCM (30 mL). The mixture was refluxed during 4 hours until the end of emission of HCl. Obtained product was carefully hydrolyzed by 5 % HCl, then extracted by CH_2Cl_2, (3x30 mL) and dried over Na_2SO_4. After removing of the solvent the residue was recrystallized from *n*-hexane to purify the product from traces of thioanisole. Yield: 6.48 g (75 %), colorless crystals, mp. 107-108 ^{o}C.
^{1}H NMR (400 MHz, $CDCl_3$), δ, ppm: 2.535 s (3H, SCH_3), 7.15 t (2H, $^{3}J_{HH}$ = 8.6 Hz), 7.29 d (2H, $^{3}J_{HH}$ = 8.4 Hz), 7.71 d (2H, $^{3}J_{HH}$ = 8.5 Hz), 7.81 dd (2H, $^{3}J_{HH}$ = 8.7 Hz, $^{4}J_{HF}$ = 5.5 Hz). ^{13}C NMR (100 MHz, $CDCl_3$), δ, ppm: 14.8 (SCH_3), 115.4 d (2C), 124.9 (2C), 130.4 (2C), 132.4 д (2C), 133.4, 133.8, 145.4, 137.7, 145.2, 165.2 д ($^{1}J_{CF}$ = 254 Hz), 194.3 (C=O). ^{19}F NMR (376 MHz, $CDCl_3$), δ, ppm: -106.3. HRMS, m/z, calculated: 247.0588, found: 247.0598 $[MH]^+$.

4'-(methylthio)phenyl(4'-chlorophenyl)methanone: yield 4.3 g (70 %), colorless crystals, mp. 132-133 ^{o}C.
^{1}H NMR (400 MHz, $CDCl_3$), δ, ppm: 2.524 s (3H, SCH_3), 7.28 d (2H, $^{3}J_{HH}$ = 8.5 Hz), 7.44 d (2H, $^{3}J_{HH}$ = 8.5 Hz), 7.70 d (2H, $^{3}J_{HH}$ = 8.7 Hz), 7.71 d (2H, $^{3}J_{HH}$ = 8.8 Hz). ^{13}C NMR (100 MHz, $CDCl_3$), δ, ppm: 14.7 (SCH_3), 124.8 (2C), 128.5 (2C), 130.4 (2C), 131.2 (2C), 133.1, 136.1, 138.5, 145.6, 194.4 (C=O).

4'-(methylthio)phenyl(3'-fluorophenyl)methanone: yield 0.71 g (39 %), colorless crystals, mp. 67-68 ^{o}C.
^{1}H NMR (400 MHz, $CDCl_3$), δ, ppm: 2.54 s (3H, SCH_3), 7.30 d (2H, $^{3}J_{HH}$ = 8.4 Hz), 7.46-7.48 m (2H), 7.53 d (1H, $^{3}J_{HH}$ = 7.6 Hz), 7.74 d (2H, $^{3}J_{HH}$ = 8.5 Hz). ^{13}C NMR (100 MHz, $CDCl_3$), δ, ppm: 14.8 (SCH_3), 116.5 d (2C), 119.1 d, 124.9 (2C), 125.5 d (1C), 129.9 d (1C), 130.5 (2C), 133.0, 140.0 d, 145.9, 162.4 d ($^{1}J_{CF}$ = 247 Hz), 194.3 (C=O). ^{19}F NMR (376 MHz, $CDCl_3$), δ, ppm: -112.0.

4'-(methylthio)phenyl(3'-chlorophenyl)methanone: yield 5.15 g (66 %), colorless crystals, mp. 73-74 ^{o}C.
^{1}H NMR (400 MHz, $CDCl_3$), δ, ppm: 2.54 s (3H, SCH_3), 7.30 d (2H, $^{3}J_{HH}$ = 8.5 Hz), 7.41 t (1H, $^{3}J_{HH}$ = 7.8 Hz), 7.54 d (1H, J_{HH} = 8.0 Hz), 7.63 d (1H, $^{3}J_{HH}$ = 7.7 Hz), 7.73 d (3H, $^{3}J_{HH}$ = 8.5 Hz). ^{13}C NMR (100 MHz, $CDCl_3$), δ, ppm: 14.8 (SCH_3), 124.9 (2C), 127.8, 129.6, 129.7, 130.5 (2C), 132.1, 132.9, 134.5, 139.5, 146.0, 194.2 (C=O).

3. General procedure for the preparation of compounds 3 and analytical data.

3.General procedure for the synthesis of 2.2-dimethyl-4,5-diphenylfuran-3(2*H*)-one

To a solution of diazoketone **4** (1 mmol)in $CHCl_3$ (10mL) a trifluoroacetic acid (TFA, 0.1 mL) in $CHCl_3$ (5 mL) was added. The mixture was refluxed during 2 hours until complete decomposition of the initial diazoketone (controlled by TLC). After the reaction completion the mixture was washed by water/$NaHCO_3$, pure water (2x10 mL) and dried over K_2CO_3 (24 h). After removal of the solvent, about 0.9 mmol (~ 98%) of a mixture of two regioisomeric 2,2-dialkyl-4,5-diaryl-dihydrofuran-3(2*H*)-ones **3** (approximate ratio - 3:1) was obtained as a light yellow oil. The regioisomers were separated by repeated twice chromatography on plates (or on column, eluent: n-hexane/CH_2Cl_2) and around 0.66 mmol (~ 72 %) of desired 3(2*H*)furanone was obtained**,** slightly yellow crystals. The compounds **3** were described previously [38,

3Medvedev, J.; A New Powerful Approach to Multi-Substituted 3(2H)-Furanones via Brønsted Acid-Catalyzed Reactions of 4-Diazodihydrofuran-3-ones. *Synthesis*. **2016**, *48*(24), 4525-4532].

3.1 Mixture of 2,2-dimethyl-4-phenyl-5-(3'-chlorophenyl)furan-3(*2H*)-one and 2,2-dimethyl-5-phenyl-4-(3'-chlorophenyl)furan-3(*2H*)-one

^{1}H NMR of mixture (300 MHz, 15 mg in 0.5 mL $CDCl_3$, reference: $CHCl_3$ = 7.26 ppm), δ, ppm: 1.56 s (6H, 2CH_3), 7.23-7.50 m (8-9H), 7.64-7.72 m (1.5H). ^{13}C NMR of mixture (75 MHz, 20 mg in 0.7 mL $CDCl_3$, reference: $CHCl_3$ = 77.00 ppm), δ, ppm: 205.25, 204.77, 178.68, 176.21, 134.55, 134.32, 132.06, 131.86, 131.61, 129.76, 129.59, 129.37, 128.67, 128.50, 128.35, 128.01, 127.80, 127.59, 126.67, 124.06, 114.28, 112.27, 87.29, 87.15, 23.27.

2,2-dimethyl-5-phenyl-4-(4'-chlorophenyl)furan-3(*2H*)-one:
^{1}H NMR (300 MHz, 15 mg in 0.6 mL $CDCl_3$, reference: $CHCl_3$ = 7.26 ppm), δ, ppm: 1.57 s (6H, 2CH_3), 7.25-7.51 m (7H), 7.65 d (2H, J = 10.1 Hz). ^{13}C NMR (75 MHz, 15 mg in 0.6 ml $CDCl_3$, reference: $CHCl_3$ = 77.00 ppm), δ, ppm: 205.08, 178.50, 133.29, 131.99, 130.72, 129.63, 128.78, 128.49, 128.42, 128.33, 112.33, 87.24, 23.29.

4. Assessment of cell growth of MCF-7 cells in culture after their treatment with increasing concentrations of the tested compounds

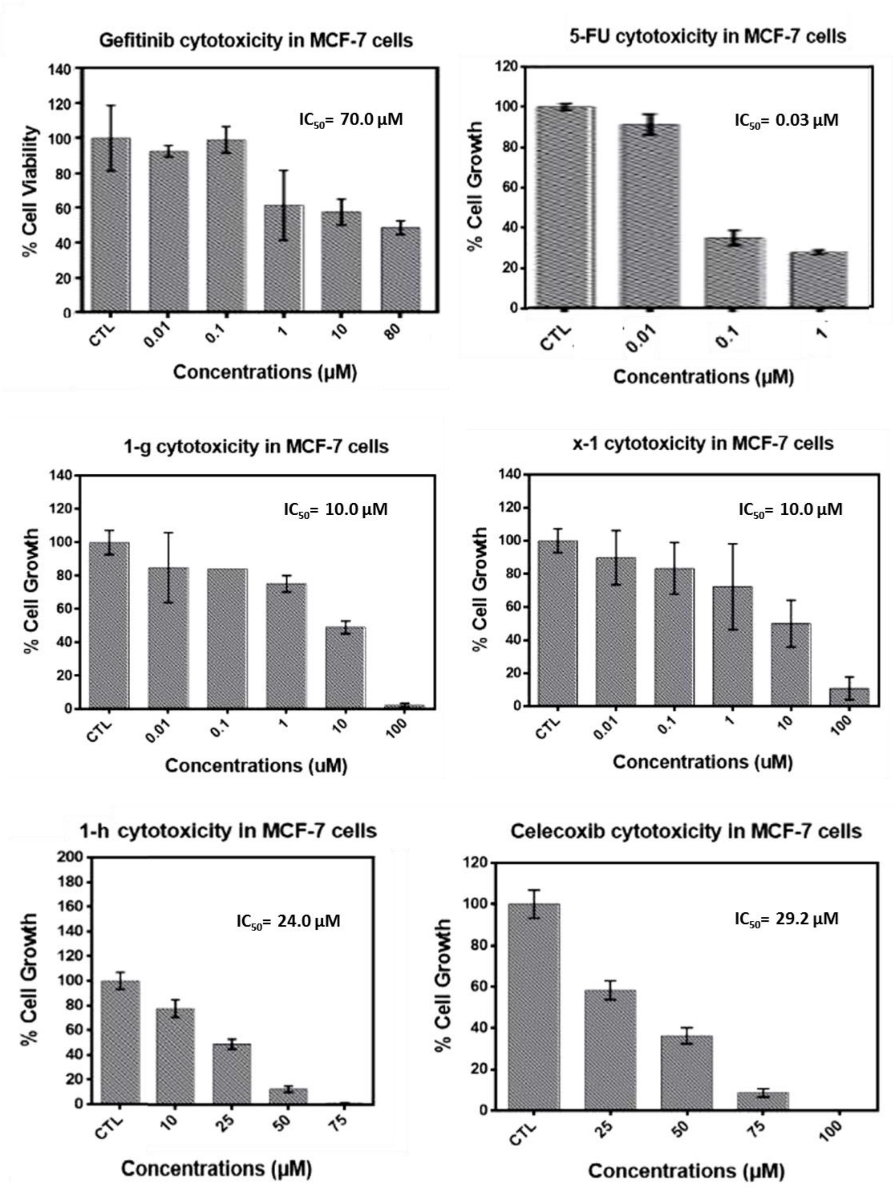

Figure S2. Assessment of cell growth of MCF-7 cells in culture after their treatment with increasing concentrations of the tested compounds for 48 hours. Cell growth was assessed using a hemocytometer (Neubauer chamber) and is expressed as a percentage (%) relative to that for the untreated, control culture CTL). The data shown above indicate a representative experiment where 4 independent measurements for each concentration were used to calculate the average (± SD, Standard Deviation).

4. Compute modeling of pharmacological activity

List of the common terms and abbreviations used in the field of virtual screening.

Docking – computational approach to perform 3D analysis of supramolecular interactions between a ligand and biological target;

- **Pre-processing** – energy minimization procedure and optimization of a biomolecular structure as well as ligand to obtain reliable 3D coordinates using the classical methods of molecular mechanics and dynamics;
- **Annealing** – reconstruction of 3D structure of selected protein with a potential energy close to the predicted minimum;
- **Stress** – conformational clashes of amino acid residues observed within the constructed protein structure;
- **Dock Scoring** – energetic function (kcal/mol) routinely used for the assessment of ligand affinity towards a target;
- **RMSD** - Root-mean-square deviation of atomic position.

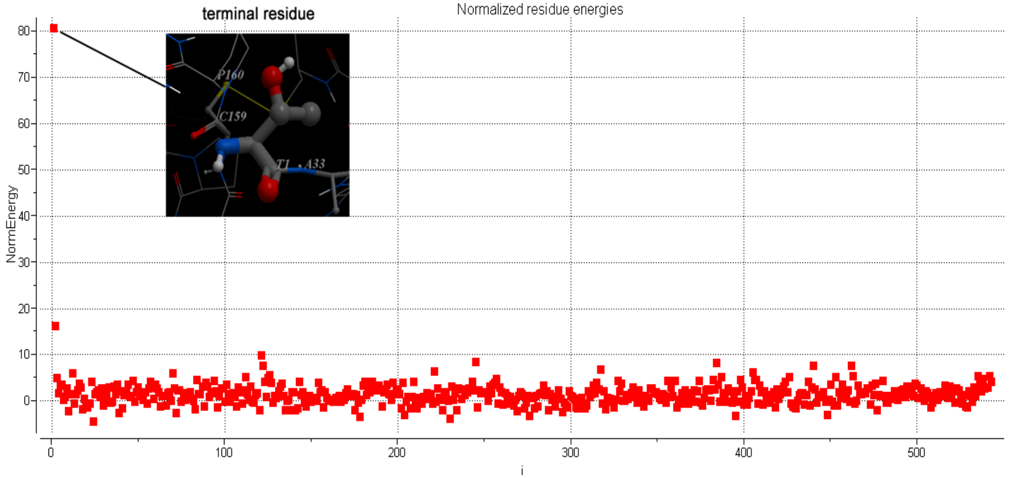

(a)

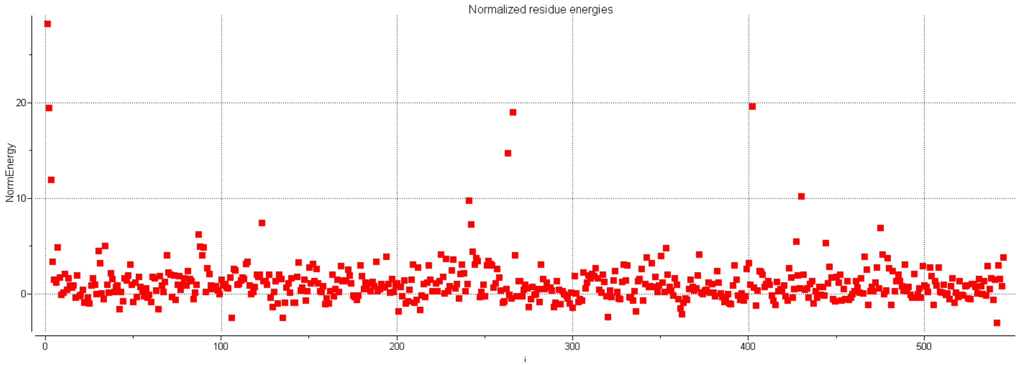

(**b**)

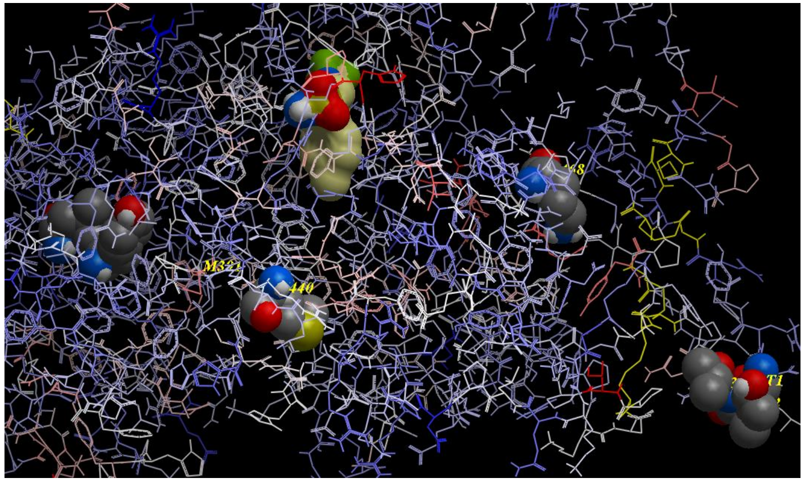

(**c**)

Figure S3 (a, b, c). Potential energy (NormEnergy, kcal/mol) shared by amino acids in *h*COX-2 (**a**) and *h*COX-1 (**b**) after annealing; **c**) amino acids in *h*COX-1 with high energy values (*grey spheres*); the binding site (*yellow sphere*).

5. Spectra

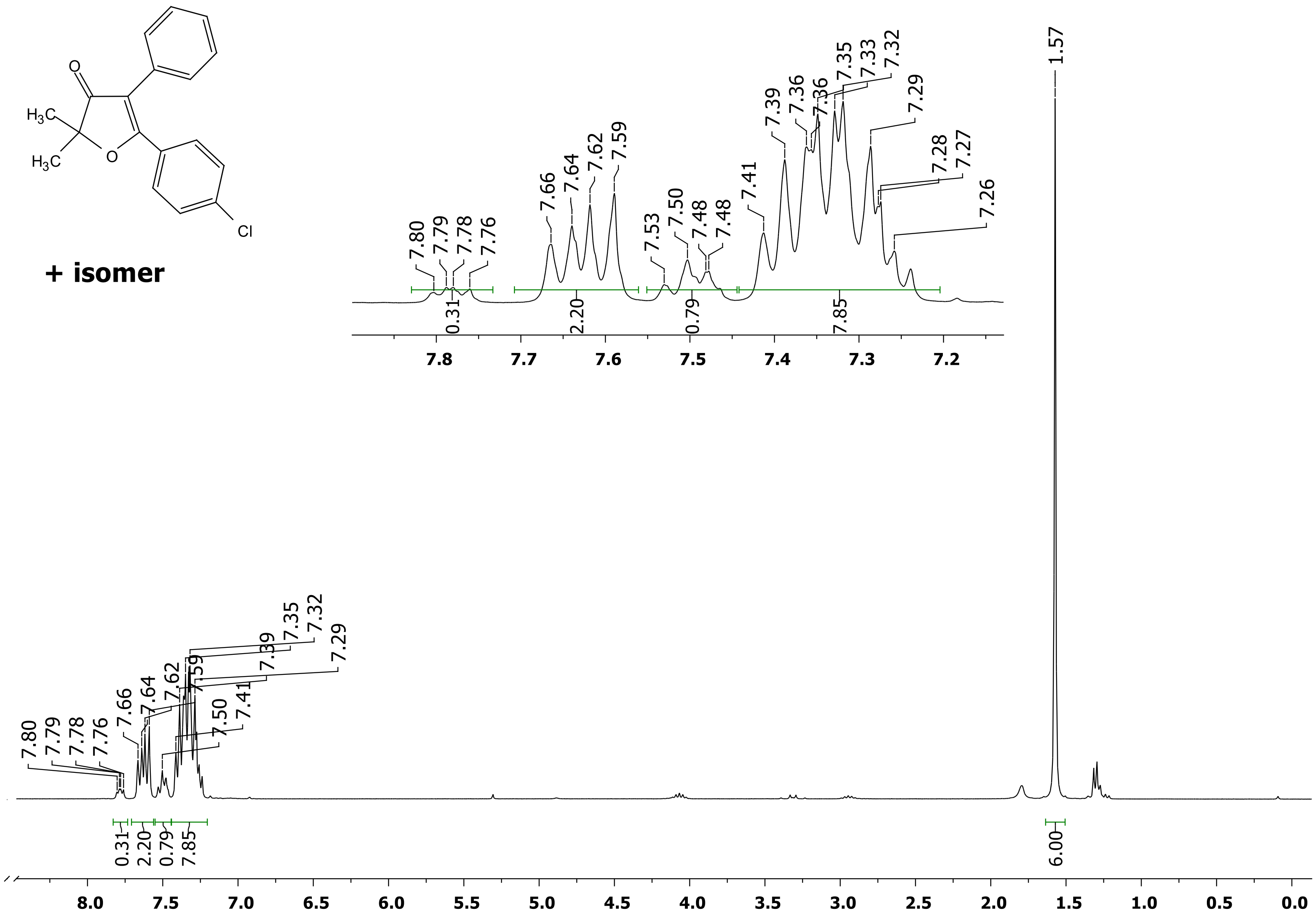
O
H_3C
H_3C
O
Cl
+ isomer
7.80
7.79
7.78
7.76
7.66
7.64
7.62
7.59
7.53
7.50
7.48
7.48
7.41
7.39
7.36
7.36
7.35
7.33
7.32
7.29
7.28
7.27
7.26
0.31
2.20
0.79
7.85
7.8
7.7
7.6
7.5
7.4
7.3
7.2
1.57
6.00
8.0
7.5
7.0
6.5
6.0
5.5
5.0
4.5
4.0
3.5
3.0
2.5
2.0
1.5
1.0
0.5
0.0

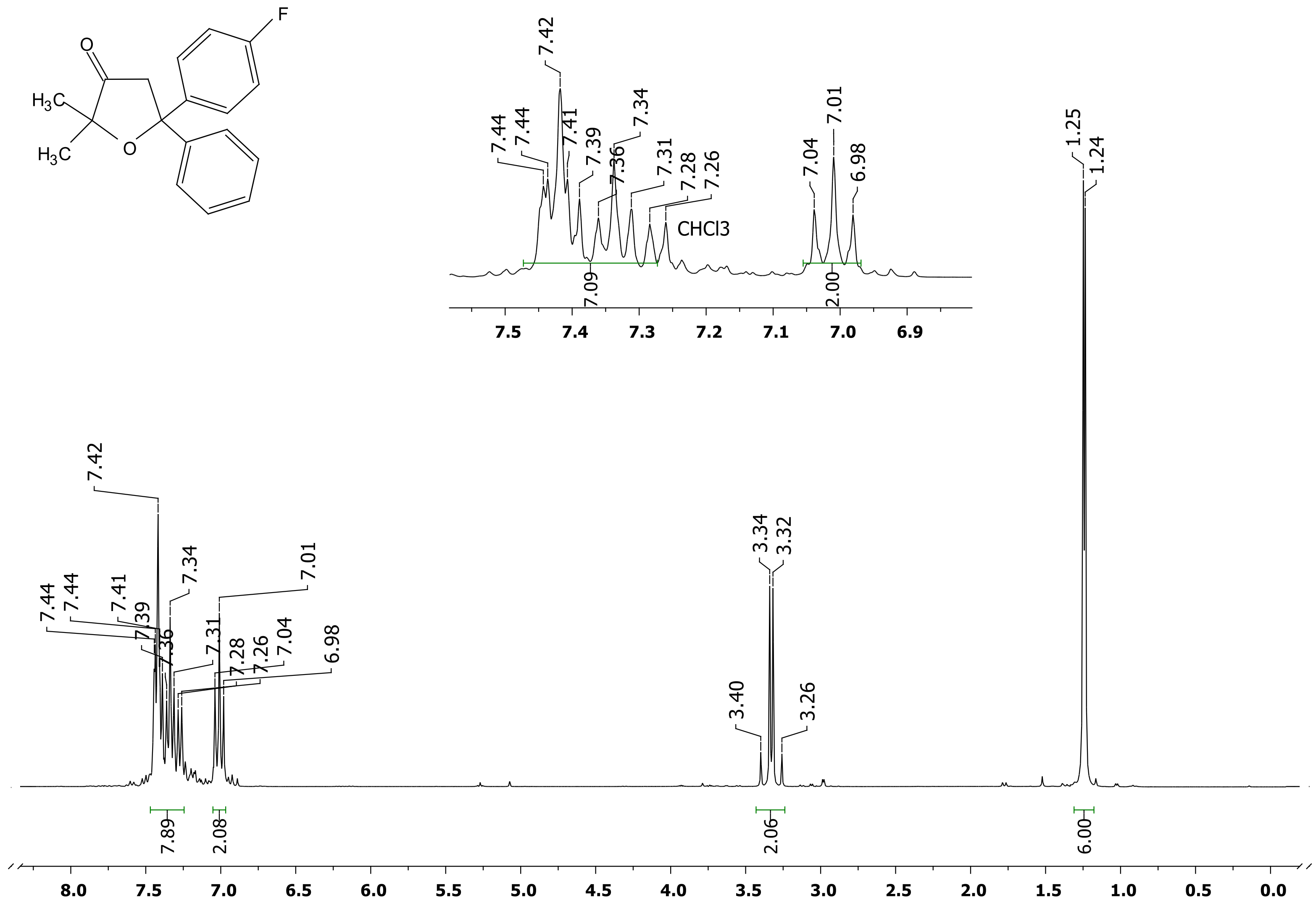
F
O
H3C
H3C
O
7.44
7.44
7.42
7.41
7.39
7.36
7.34
7.31
7.28
7.26
CHCl3
7.04
7.01
6.98
7.09
2.00
7.5
7.4
7.3
7.2
7.1
7.0
6.9
3.40
3.34
3.32
3.26
1.25
1.24
7.89
2.08
2.06
6.00
8.0
7.5
7.0
6.5
6.0
5.5
5.0
4.5
4.0
3.5
3.0
2.5
2.0
1.5
1.0
0.5
0.0

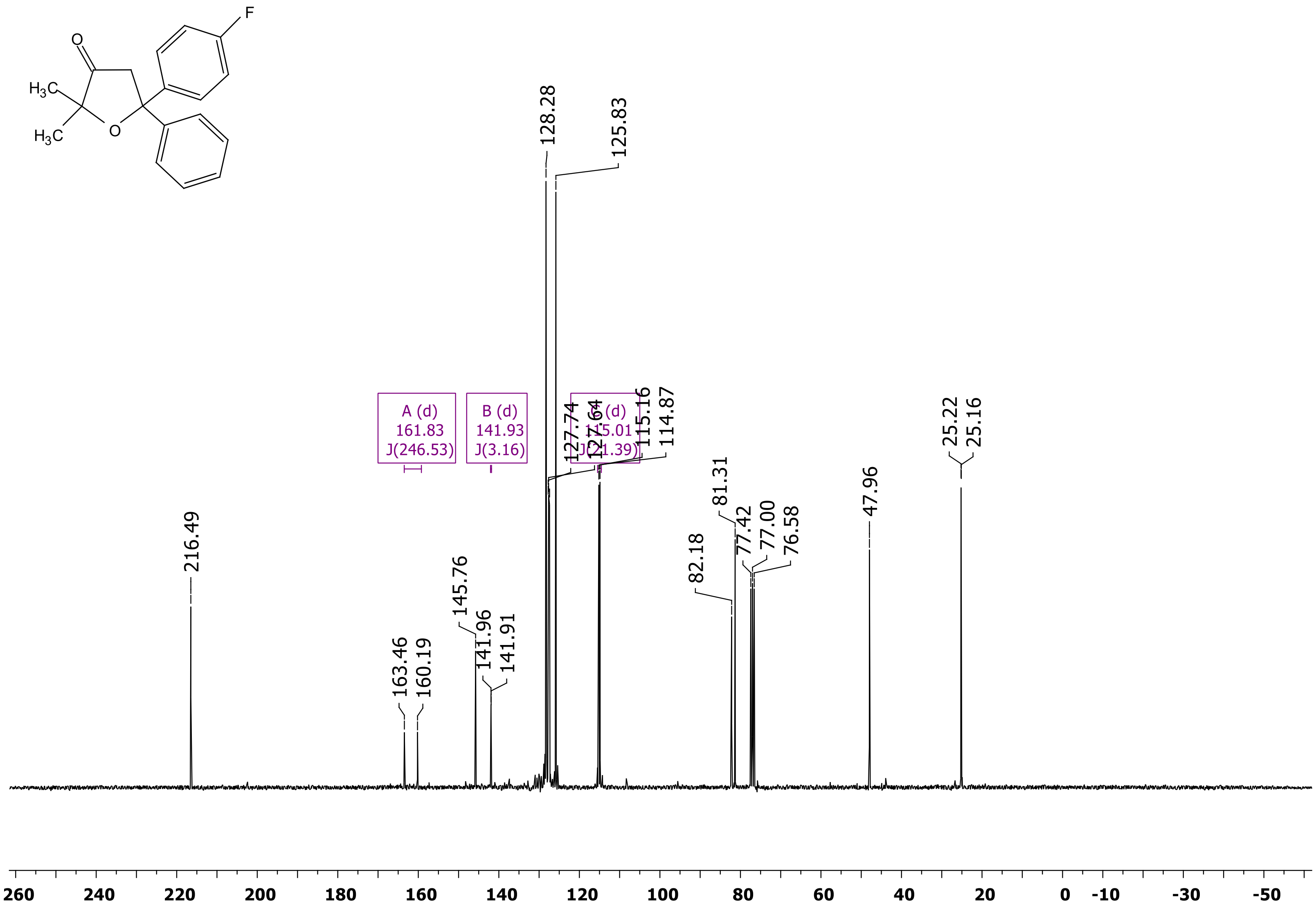
F
O
H3C
H3C
O
128.28
125.83
A (d)
161.83
J(246.53)
B (d)
141.93
J(3.16)
115.01
127.74
127.64
115.16
114.87
25.22
25.16
47.96
81.31
77.42
77.00
76.58
82.18
216.49
145.76
141.96
141.91
163.46
160.19
260
240
220
200
180
160
140
120
100
80
60
40
20
0
-10
-30
-50

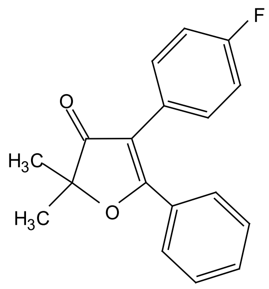
F
O
H3C
H3C
O

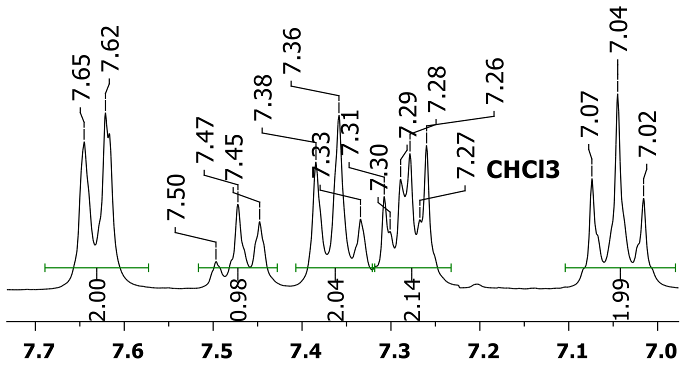
7.65
7.62
7.50
7.47
7.45
7.38
7.36
7.33
7.31
7.30
7.29
7.28
7.27
7.26
CHCl3
7.07
7.04
7.02
2.00
0.98
2.04
2.14
1.99
7.7
7.6
7.5
7.4
7.3
7.2
7.1
7.0

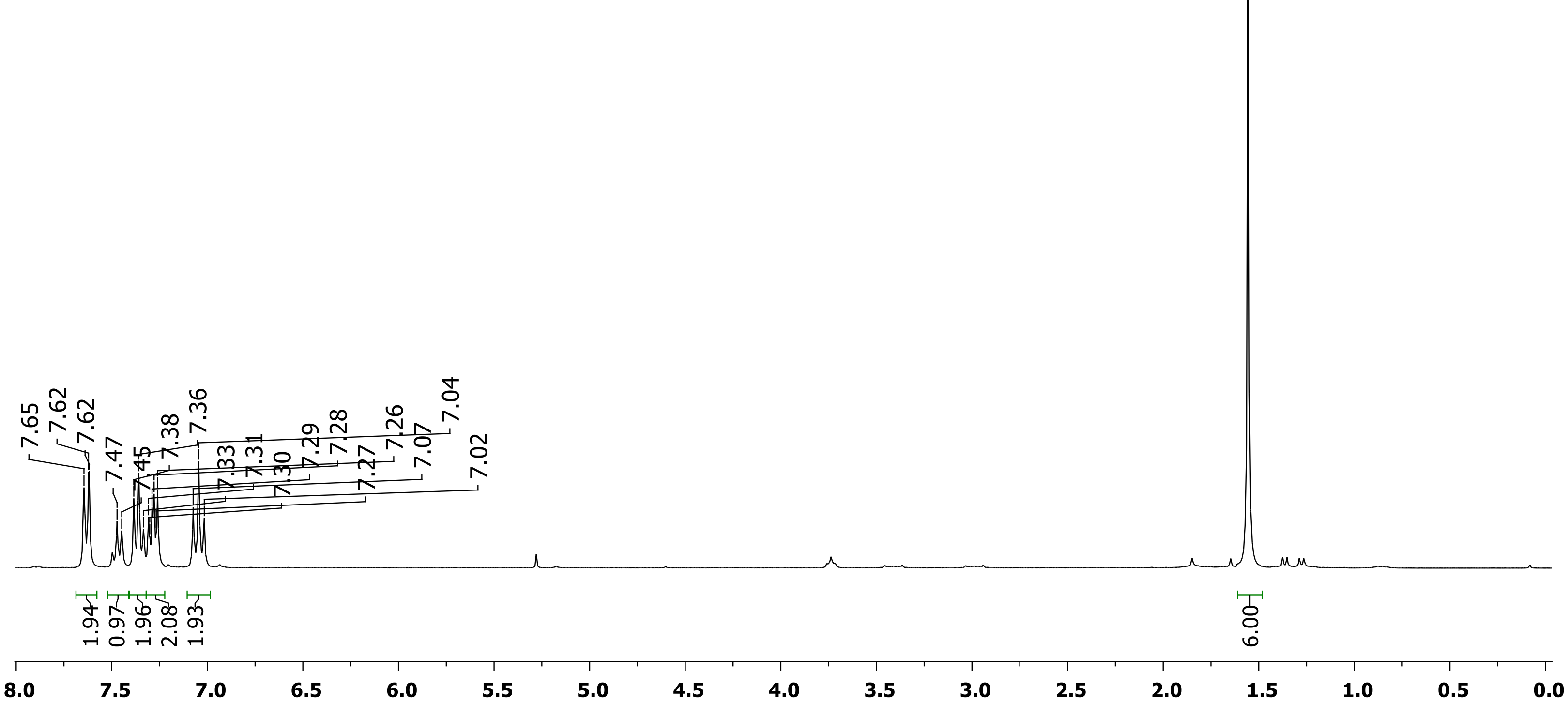
1.56
1.94
0.97
1.96
2.08
1.93
6.00
8.0
7.5
7.0
6.5
6.0
5.5
5.0
4.5
4.0
3.5
3.0
2.5
2.0
1.5
1.0
0.5
0.0

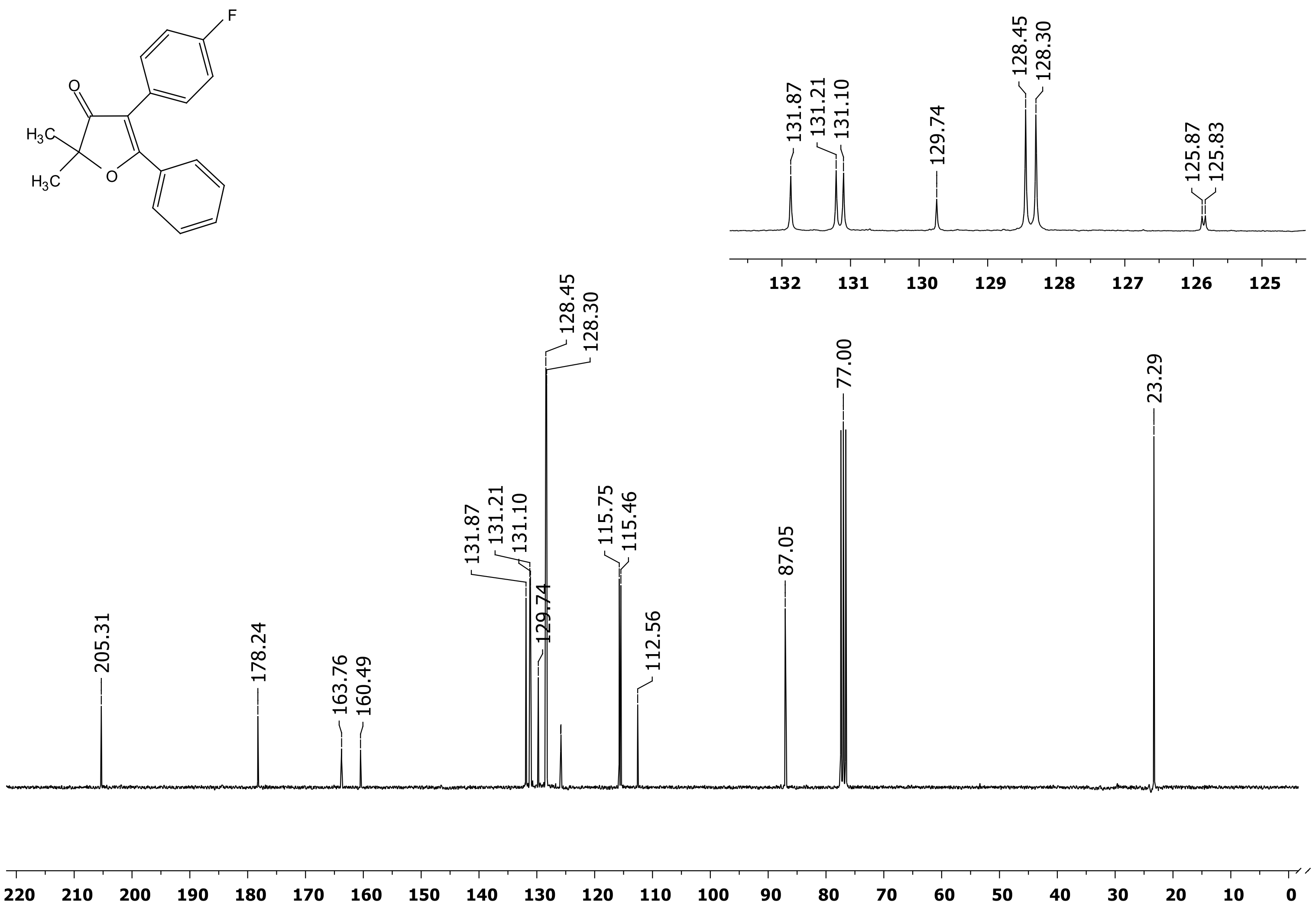
F
O
H3C
H3C
O
131.87
131.21
131.10
129.74
128.45
128.30
125.87
125.83
132 131 130 129 128 127 126 125
205.31
178.24
163.76
160.49
131.87
131.21
131.10
129.74
128.45
128.30
115.75
115.46
112.56
87.05
77.00
23.29
220 210 200 190 180 170 160 150 140 130 120 110 100 90 80 70 60 50 40 30 20 10 0

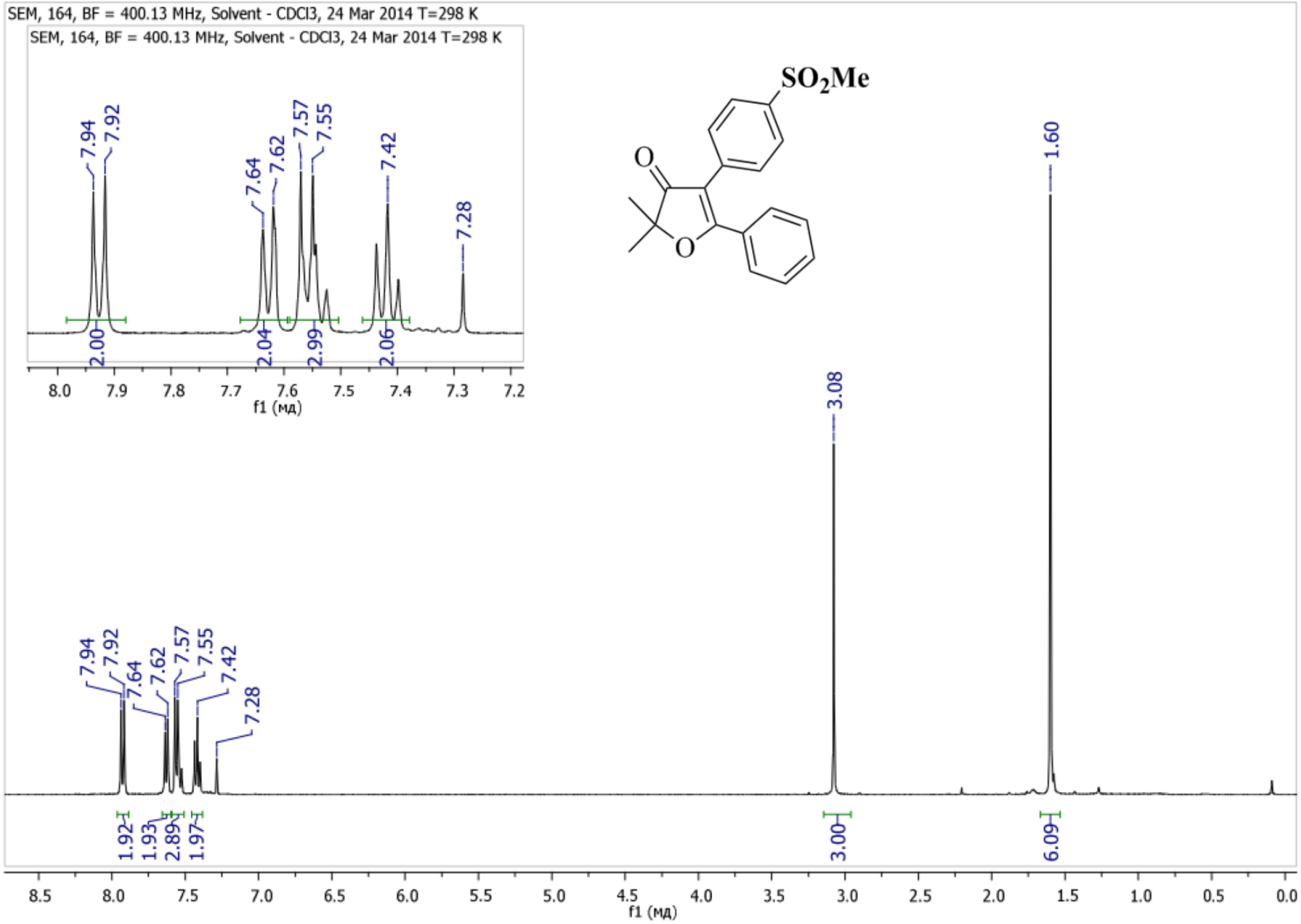

SEM, 164, BF = 400.13 MHz, Solvent - CDCl3, 24 Mar 2014 T=298 K
SEM, 164, BF = 400.13 MHz, Solvent - CDCl3, 24 Mar 2014 T=298 K
SO2Me
O
O
7.94
7.92
7.64
7.62
7.57
7.55
7.42
7.28
2.00
2.04
2.99
2.06
f1 (мд)
1.60
3.08
1.92
1.93
2.89
1.97
3.00
6.09
f1 (мд)

6.

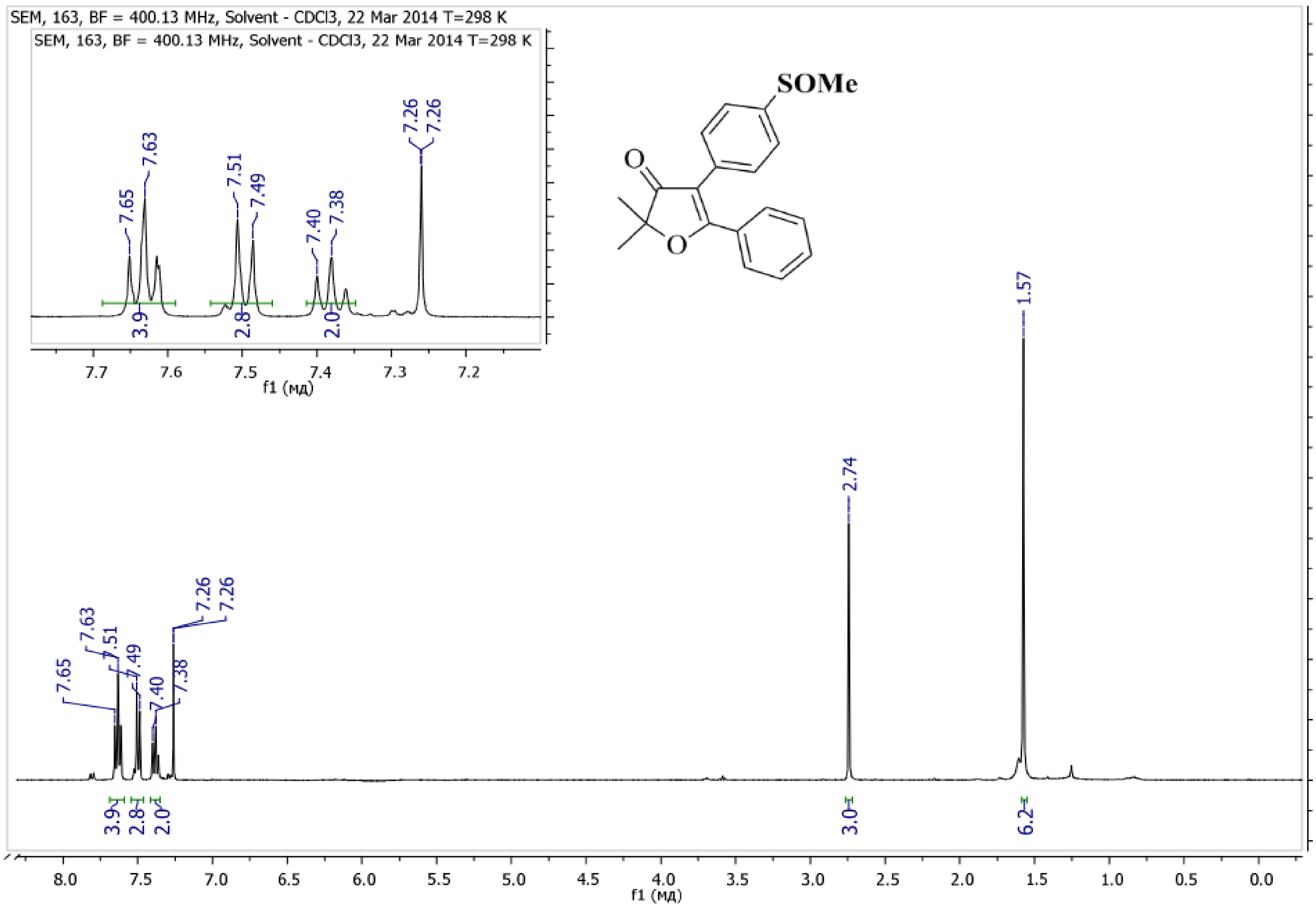
SEM, 163, BF = 400.13 MHz, Solvent - CDCl3, 22 Mar 2014 T=298 K
SEM, 163, BF = 400.13 MHz, Solvent - CDCl3, 22 Mar 2014 T=298 K
SOMe
O
O
7.65
7.63
7.51
7.49
7.40
7.38
7.26
7.26
2.74
1.57
3.9
2.8
2.0
3.0
6.2
f1 (мд)

7.

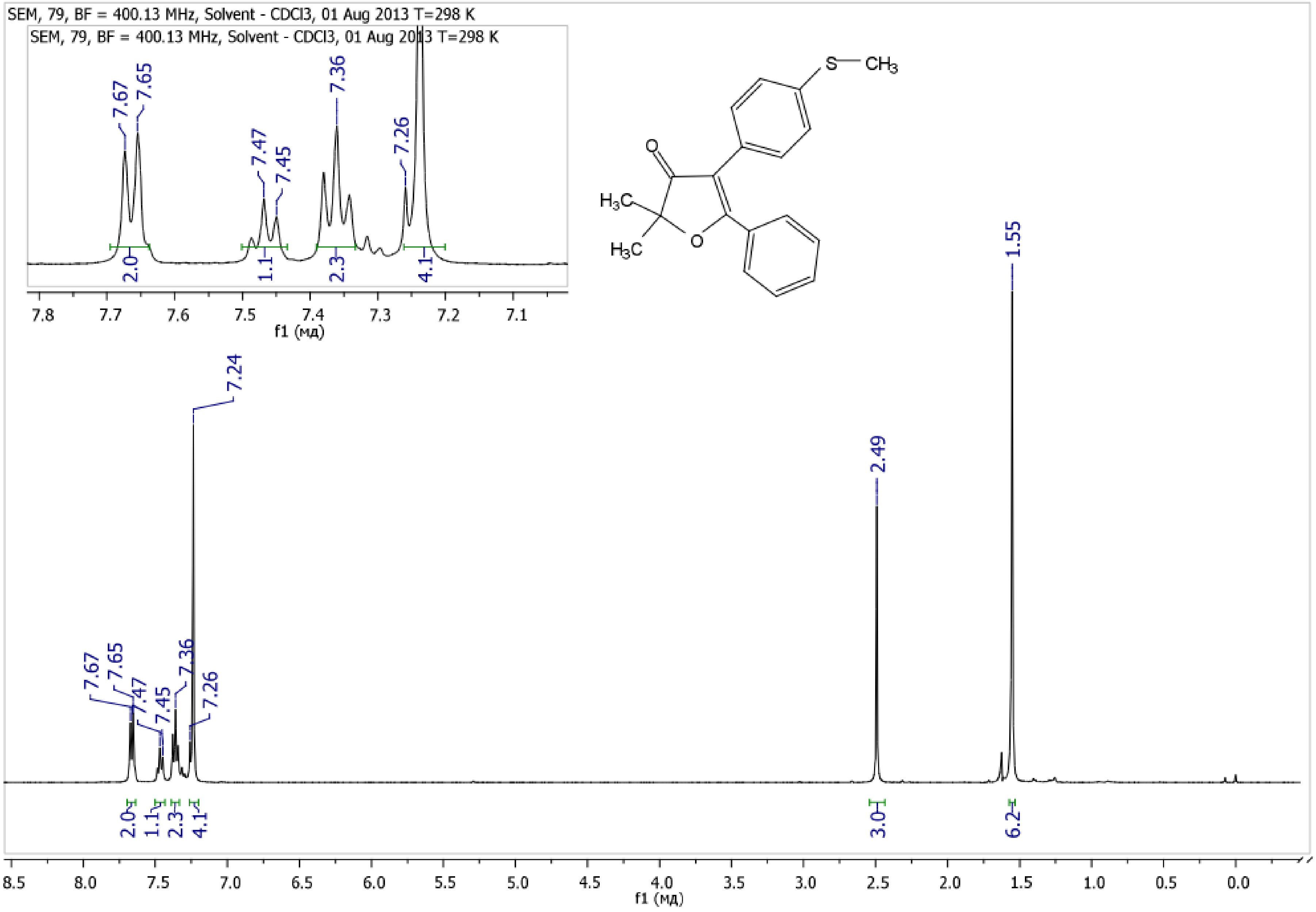
SEM, 79, BF = 400.13 MHz, Solvent - CDCl3, 01 Aug 2013 T=298 K
7.67
7.65
7.47
7.45
7.36
7.26
7.24
2.49
1.55
2.0
1.1
2.3
4.1
3.0
6.2
f1 (мд)
S
CH3
O
H3C
H3C
O

8.

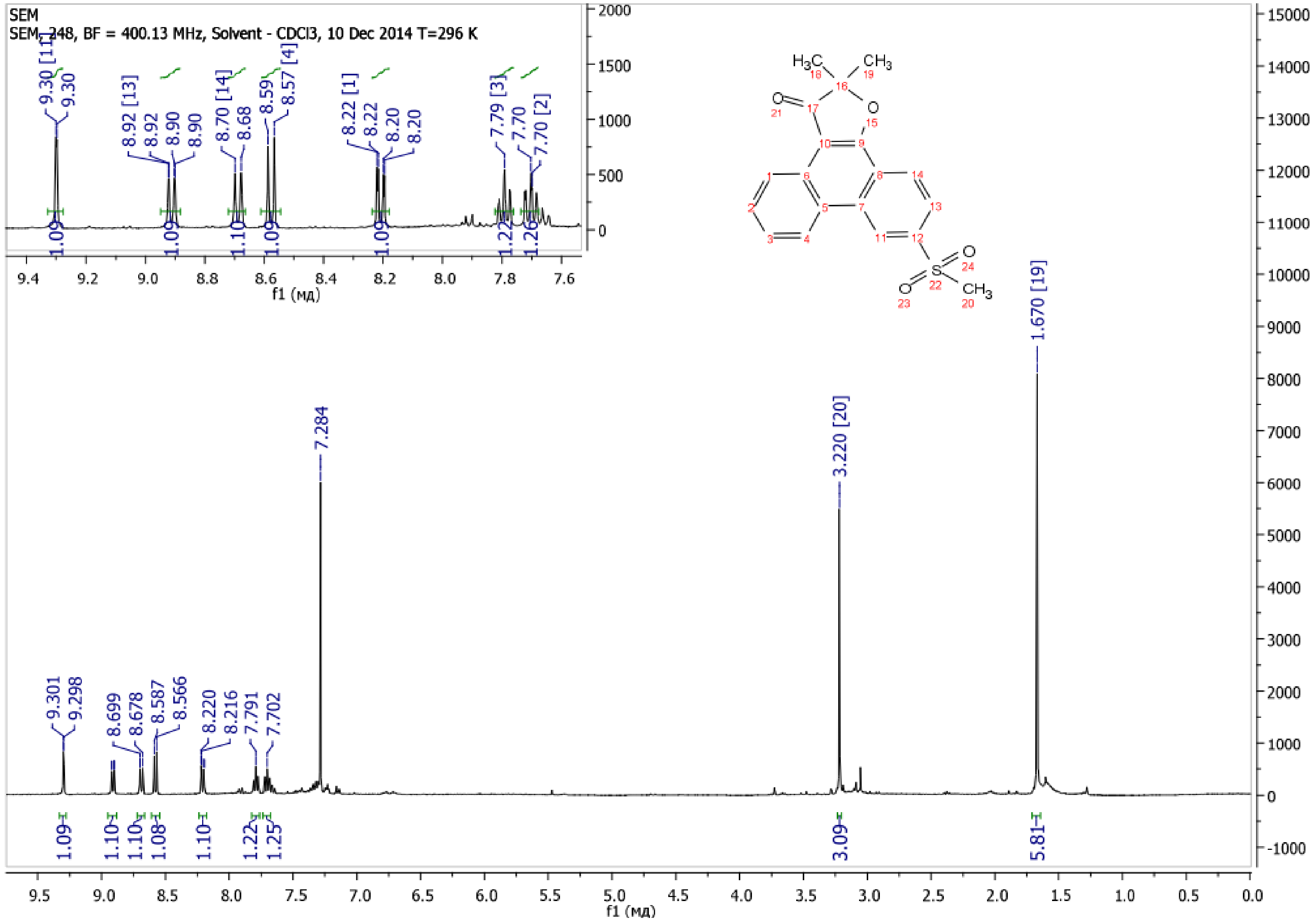
SEM
SEM_248, BF = 400.13 MHz, Solvent - CDCl3, 10 Dec 2014 T=296 K
f1 (мд)
f1 (мд)

9.

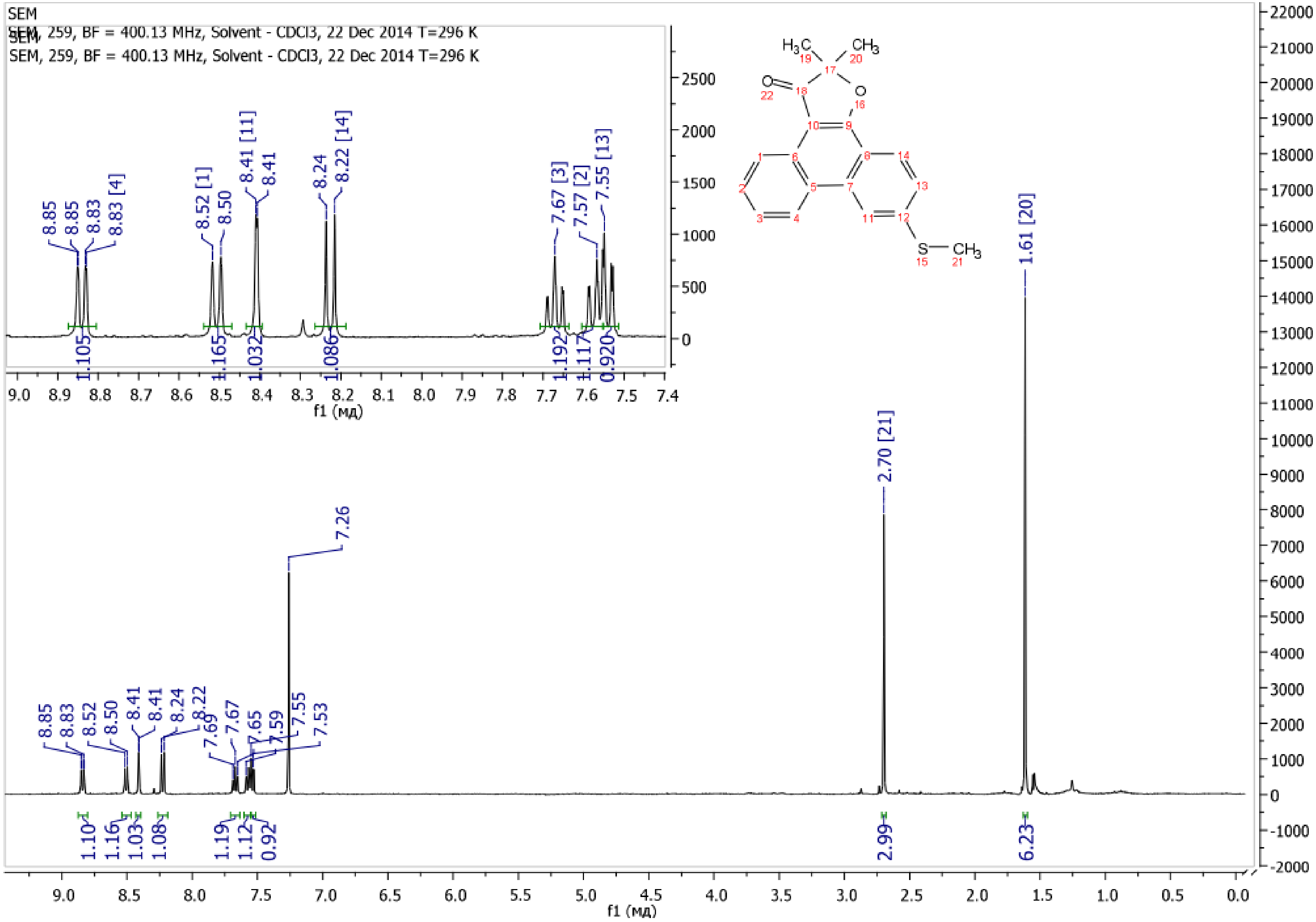

10.

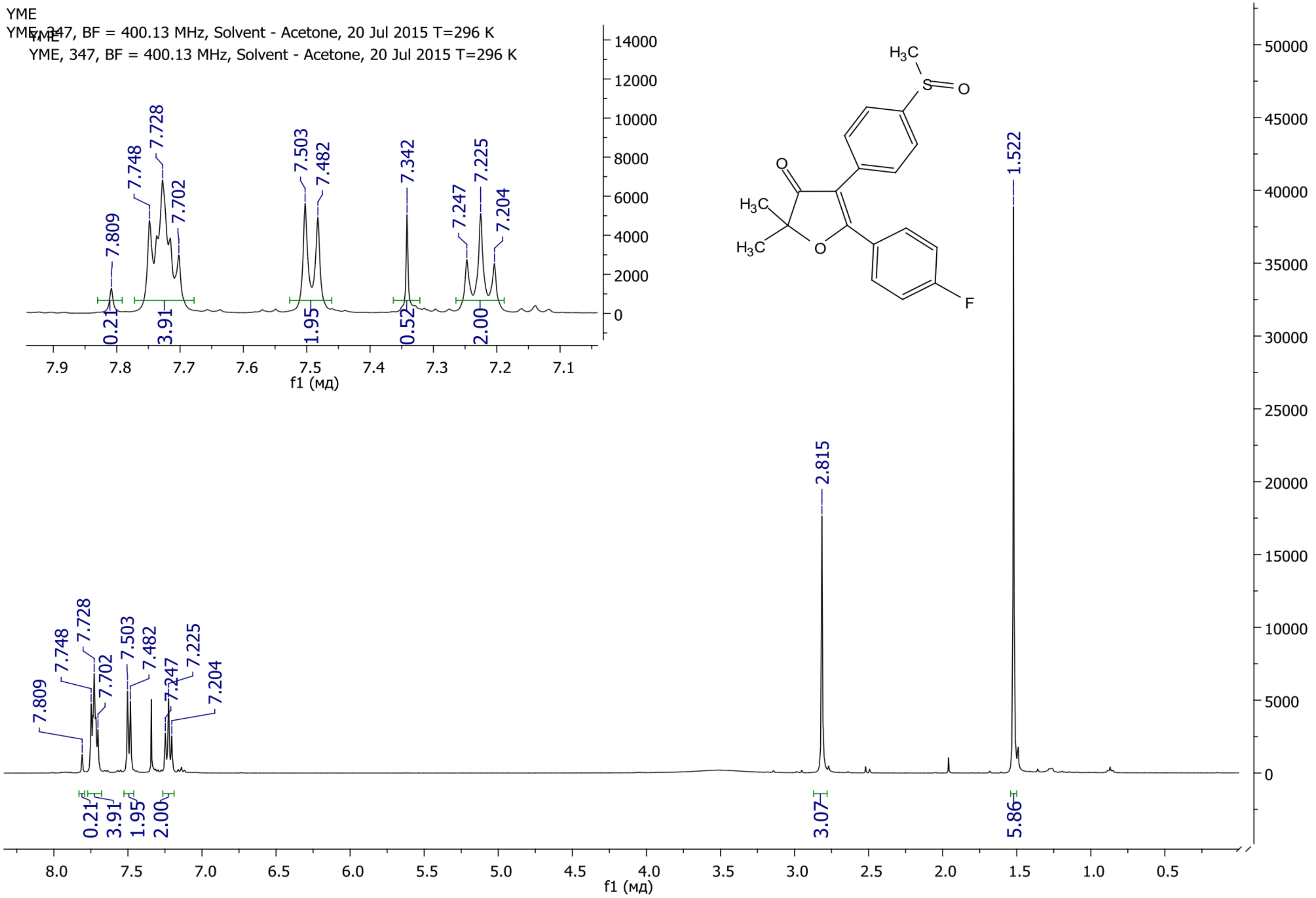
YME
YME, 347, BF = 400.13 MHz, Solvent - Acetone, 20 Jul 2015 T=296 K
YME, 347, BF = 400.13 MHz, Solvent - Acetone, 20 Jul 2015 T=296 K
7.809
7.748
7.728
7.702
7.503
7.482
7.342
7.247
7.225
7.204
2.815
1.522
0.21
3.91
1.95
0.52
2.00
3.07
5.86
f1 (мд)
H3C
S
O
O
H3C
H3C
O
F

11.

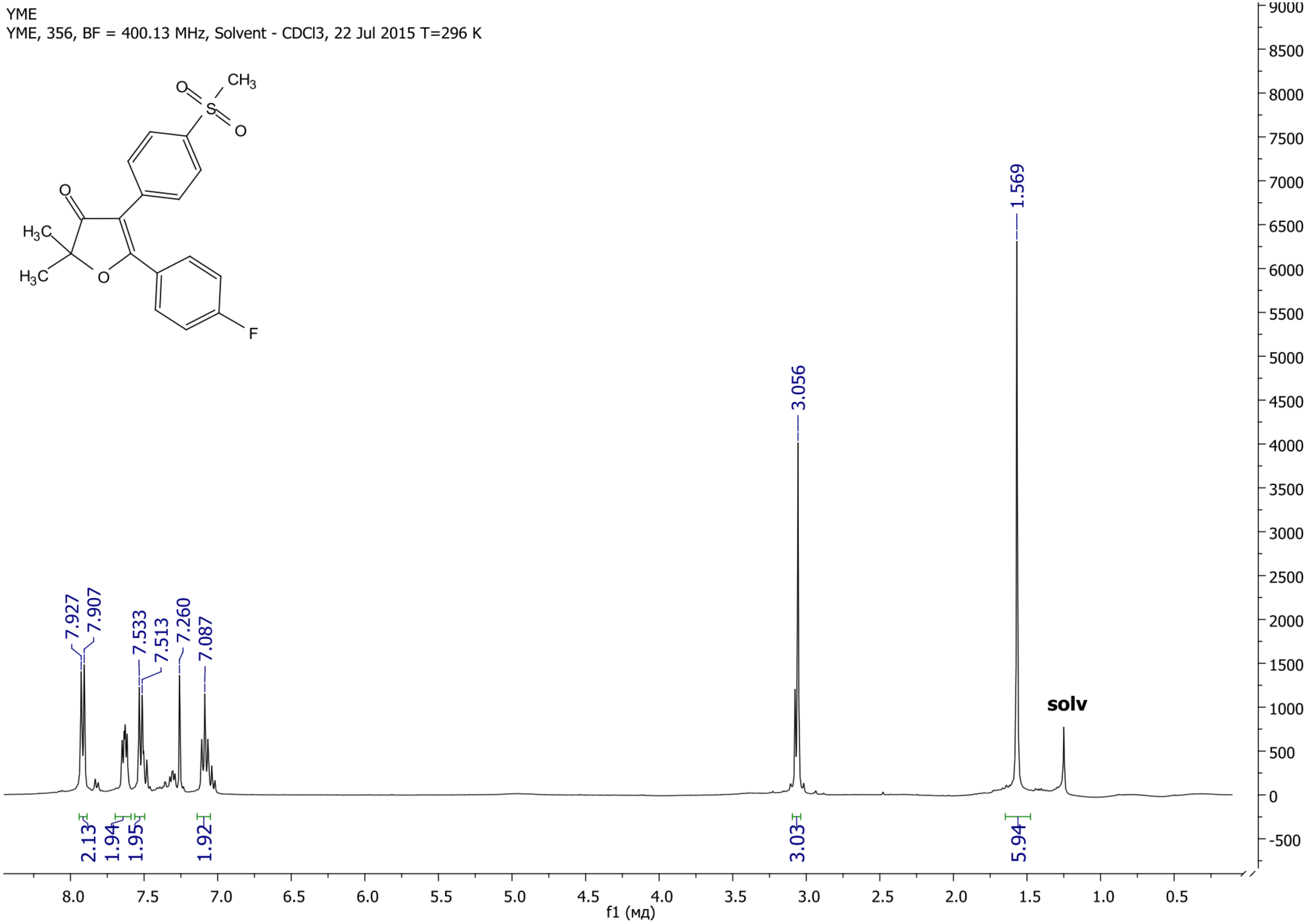
YME
YME, 356, BF = 400.13 MHz, Solvent - CDCl3, 22 Jul 2015 T=296 K
7.927
7.907
7.533
7.513
7.260
7.087
3.056
1.569
solv
2.13
1.94
1.95
1.92
3.03
5.94
f1 (мд)

12.

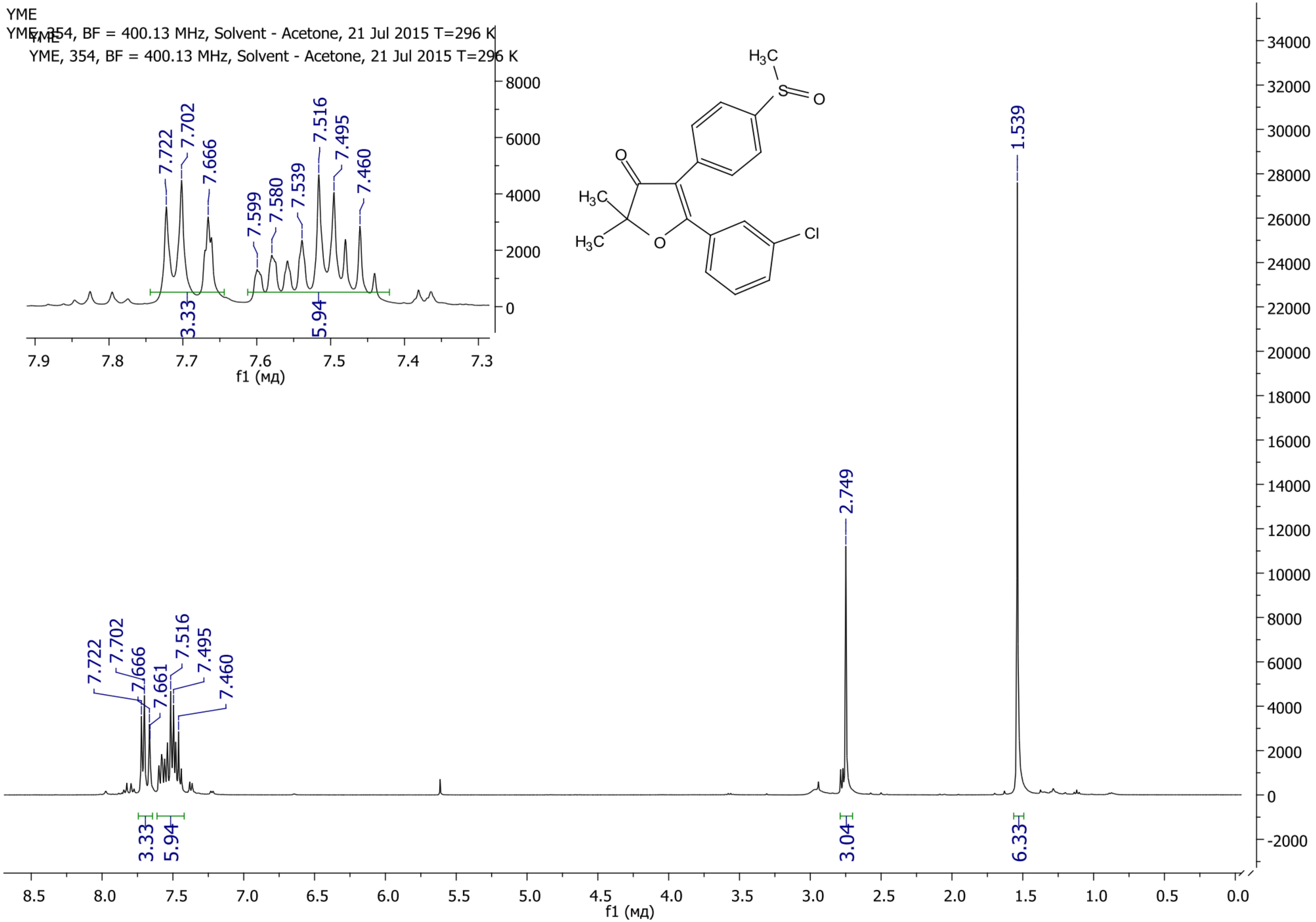

YME
YME, 354, BF = 400.13 MHz, Solvent - Acetone, 21 Jul 2015 T=296 K
YME, 354, BF = 400.13 MHz, Solvent - Acetone, 21 Jul 2015 T=296 K
7.722
7.702
7.666
7.599
7.580
7.539
7.516
7.495
7.460
3.33
5.94
f1 (мд)
H3C
S
O
O
H3C
H3C
O
Cl
1.539
2.749
7.661
3.04
6.33
f1 (мд)

13.

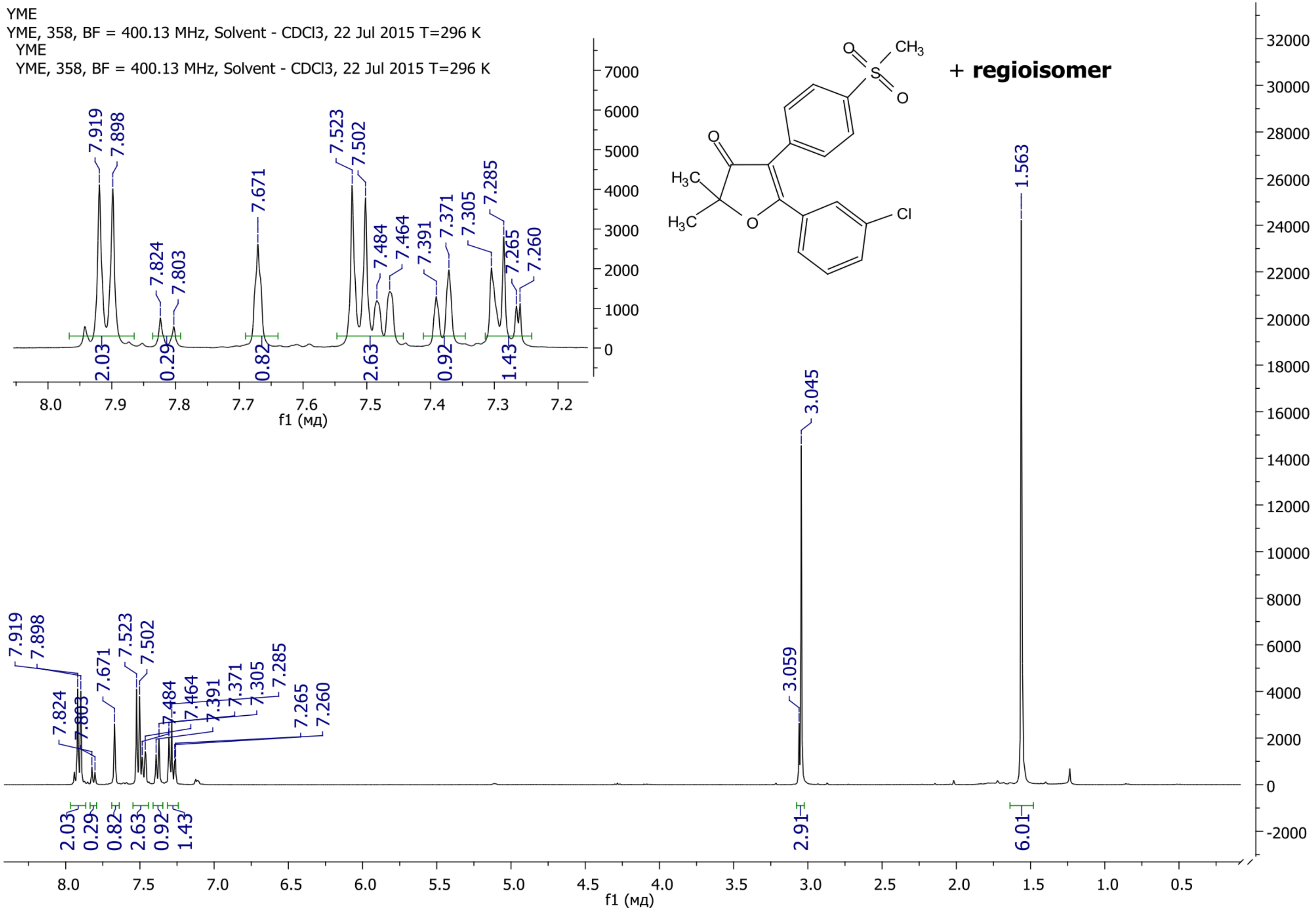
YME
YME, 358, BF = 400.13 MHz, Solvent - CDCl3, 22 Jul 2015 T=296 K
YME
YME, 358, BF = 400.13 MHz, Solvent - CDCl3, 22 Jul 2015 T=296 K
+ regioisomer
O
CH3
S
O
O
H3C
H3C
O
Cl
7.919
7.898
7.824
7.803
7.671
7.523
7.502
7.484
7.464
7.391
7.371
7.305
7.285
7.265
7.260
2.03
0.29
0.82
2.63
0.92
1.43
3.059
3.045
2.91
1.563
6.01
f1 (мд)

14.

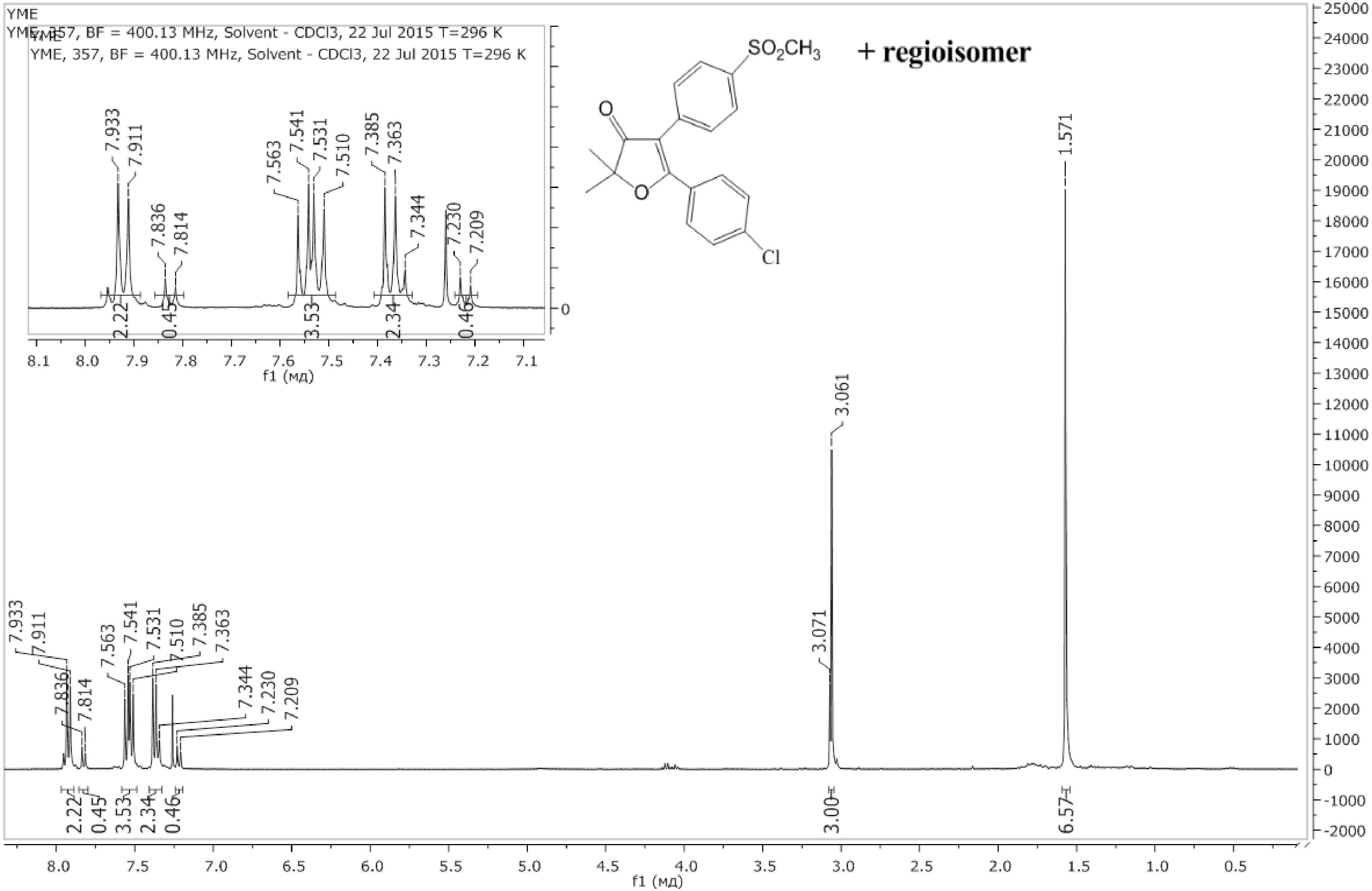
YME
YME, 357, BF = 400.13 MHz, Solvent - CDCl3, 22 Jul 2015 T=296 K
SO2CH3
+ regioisomer
O
Cl
7.933
7.911
7.836
7.814
7.563
7.541
7.531
7.510
7.385
7.363
7.344
7.230
7.209
3.071
3.061
1.571
2.22
0.45
3.53
2.34
0.46
3.00
6.57
f1 (мд)

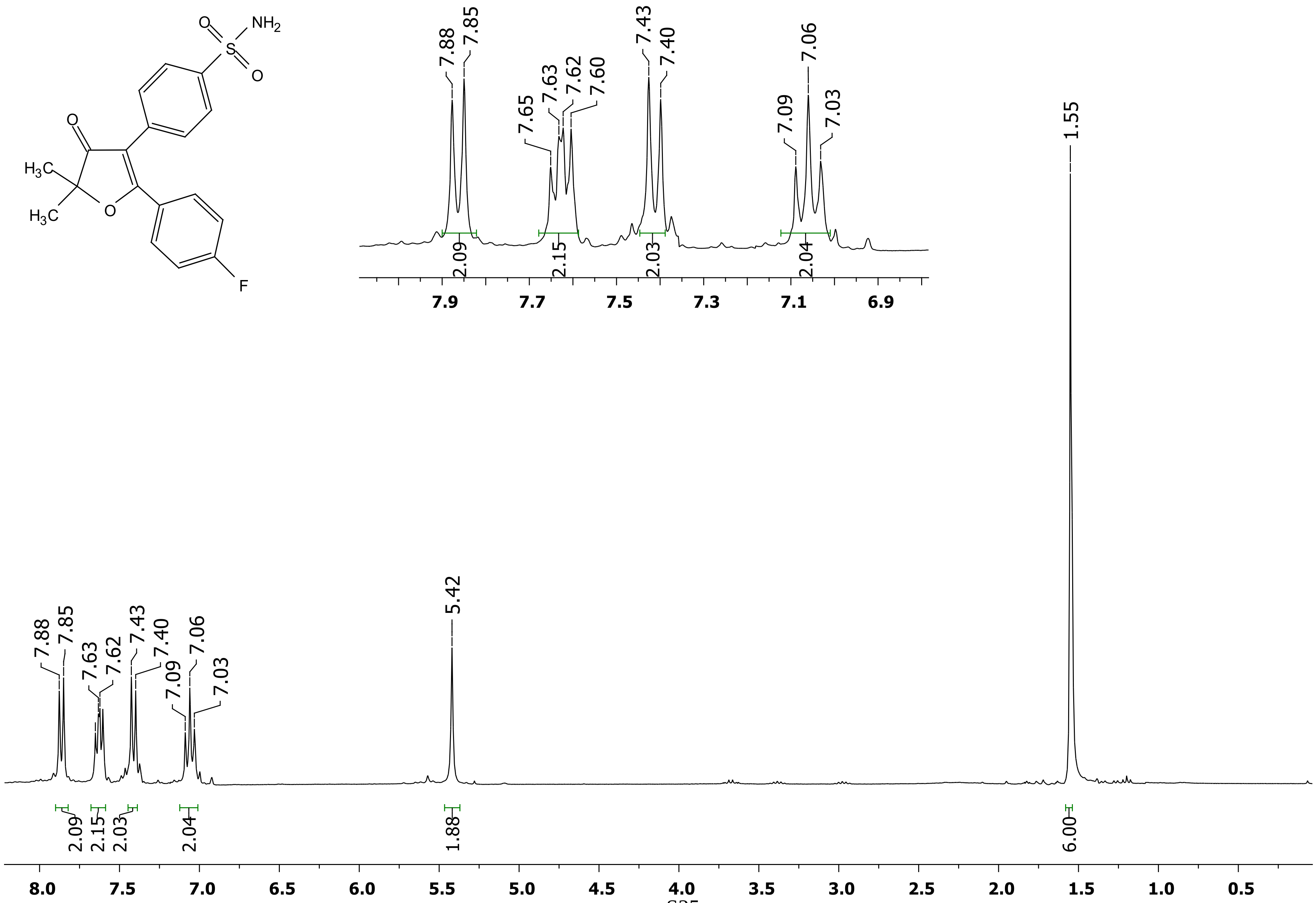

NH2
O
S
O
O
H3C
H3C
O
F
7.88
7.85
7.65
7.63
7.62
7.60
7.43
7.40
7.09
7.06
7.03
2.09
2.15
2.03
2.04
7.9
7.7
7.5
7.3
7.1
6.9
5.42
1.55
1.88
6.00
8.0
7.5
7.0
6.5
6.0
5.5
5.0
4.5
4.0
3.5
3.0
2.5
2.0
1.5
1.0
0.5

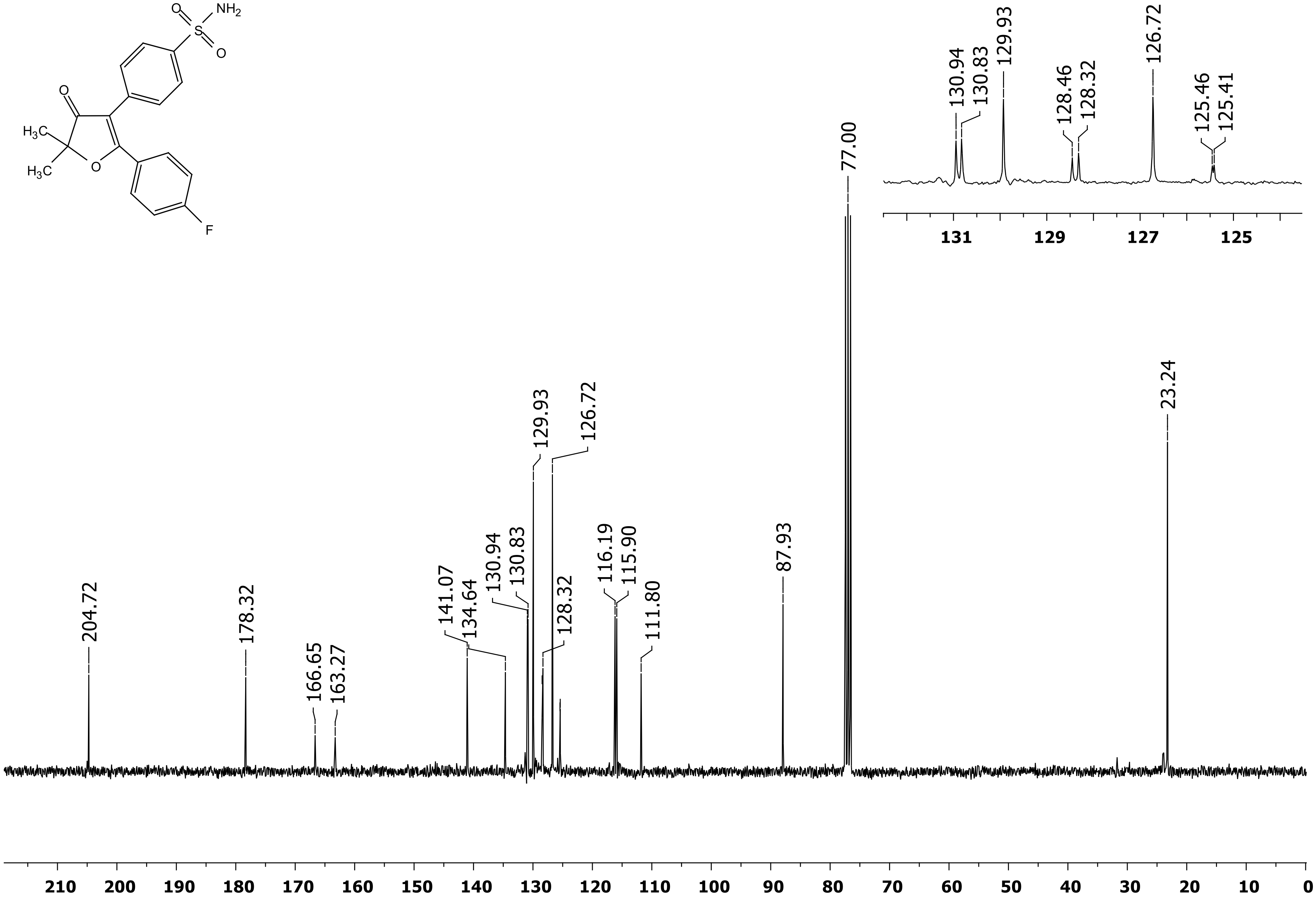
O
NH2
S
O
O
H3C
H3C
O
F
130.94
130.83
129.93
128.46
128.32
126.72
125.46
125.41
131
129
127
125
77.00
23.24
204.72
178.32
166.65
163.27
141.07
134.64
130.94
130.83
129.93
126.72
128.32
116.19
115.90
111.80
87.93
210
200
190
180
170
160
150
140
130
120
110
100
90
80
70
60
50
40
30
20
10
0

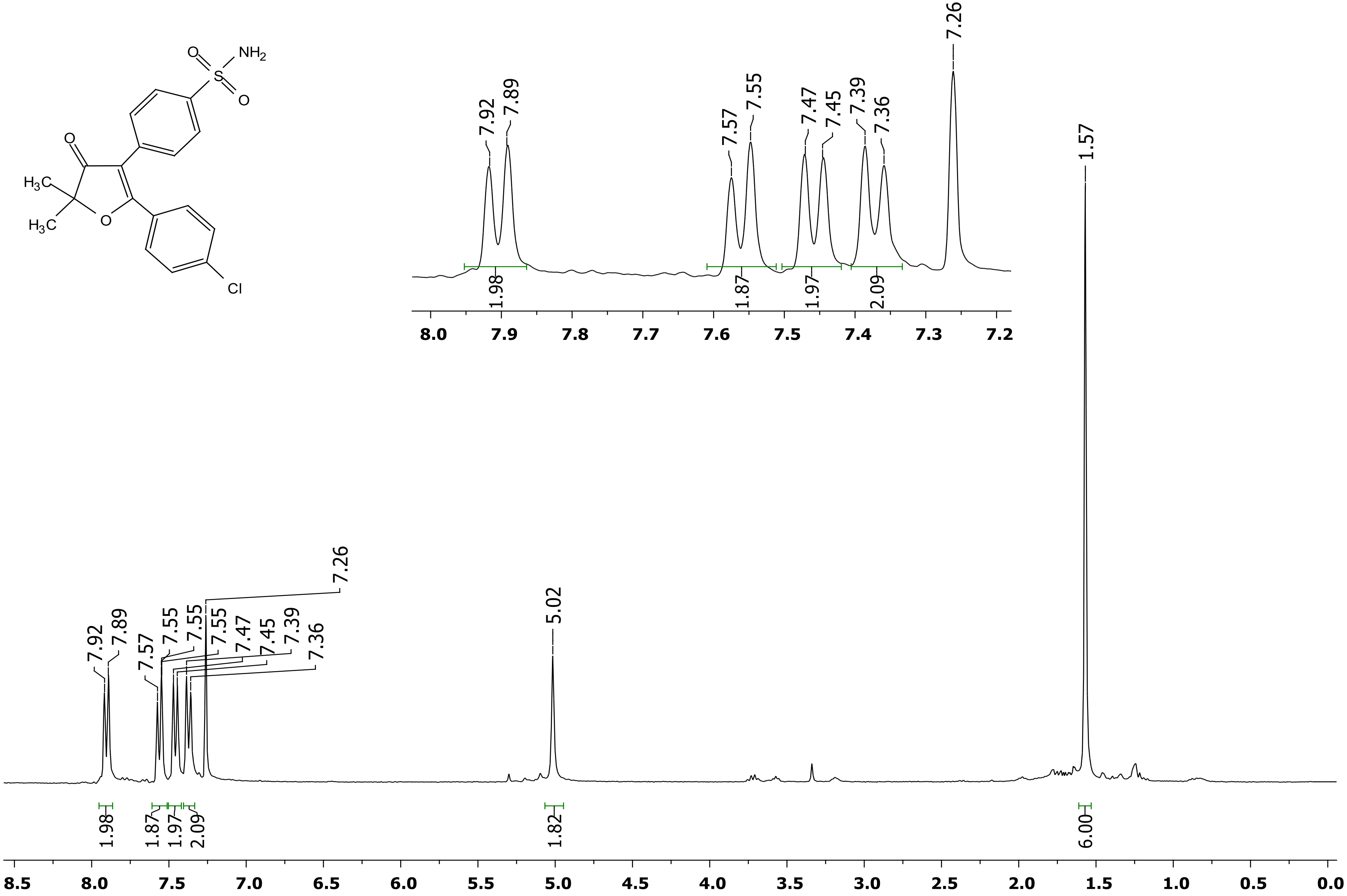

NH2
O
S
O
O
H3C
H3C
O
Cl
7.26
7.92
7.89
7.57
7.55
7.47
7.45
7.39
7.36
1.98
1.87
1.97
2.09
8.0
7.9
7.8
7.7
7.6
7.5
7.4
7.3
7.2
1.57
7.55
7.55
5.02
1.82
6.00
8.5
7.5
7.0
6.5
6.0
5.5
5.0
4.5
4.0
3.5
3.0
2.5
2.0
1.5
1.0
0.5
0.0

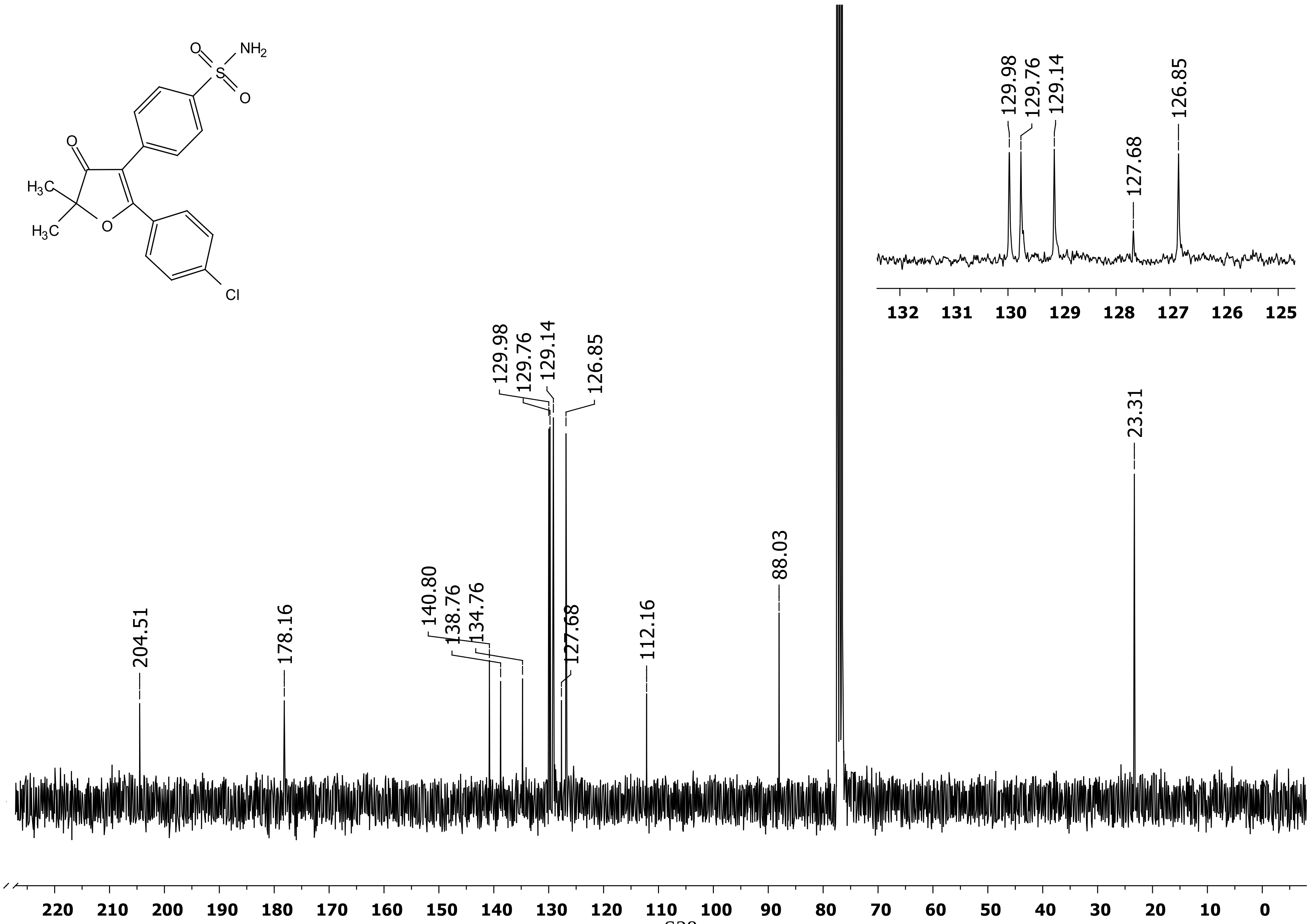
O
S
NH2
O
O
H3C
H3C
O
Cl
129.98
129.76
129.14
126.85
127.68
132 131 130 129 128 127 126 125
204.51
178.16
140.80
138.76
134.76
129.98
129.76
129.14
126.85
127.68
112.16
88.03
23.31
220 210 200 190 180 170 160 150 140 130 120 110 100 90 80 70 60 50 40 30 20 10 0

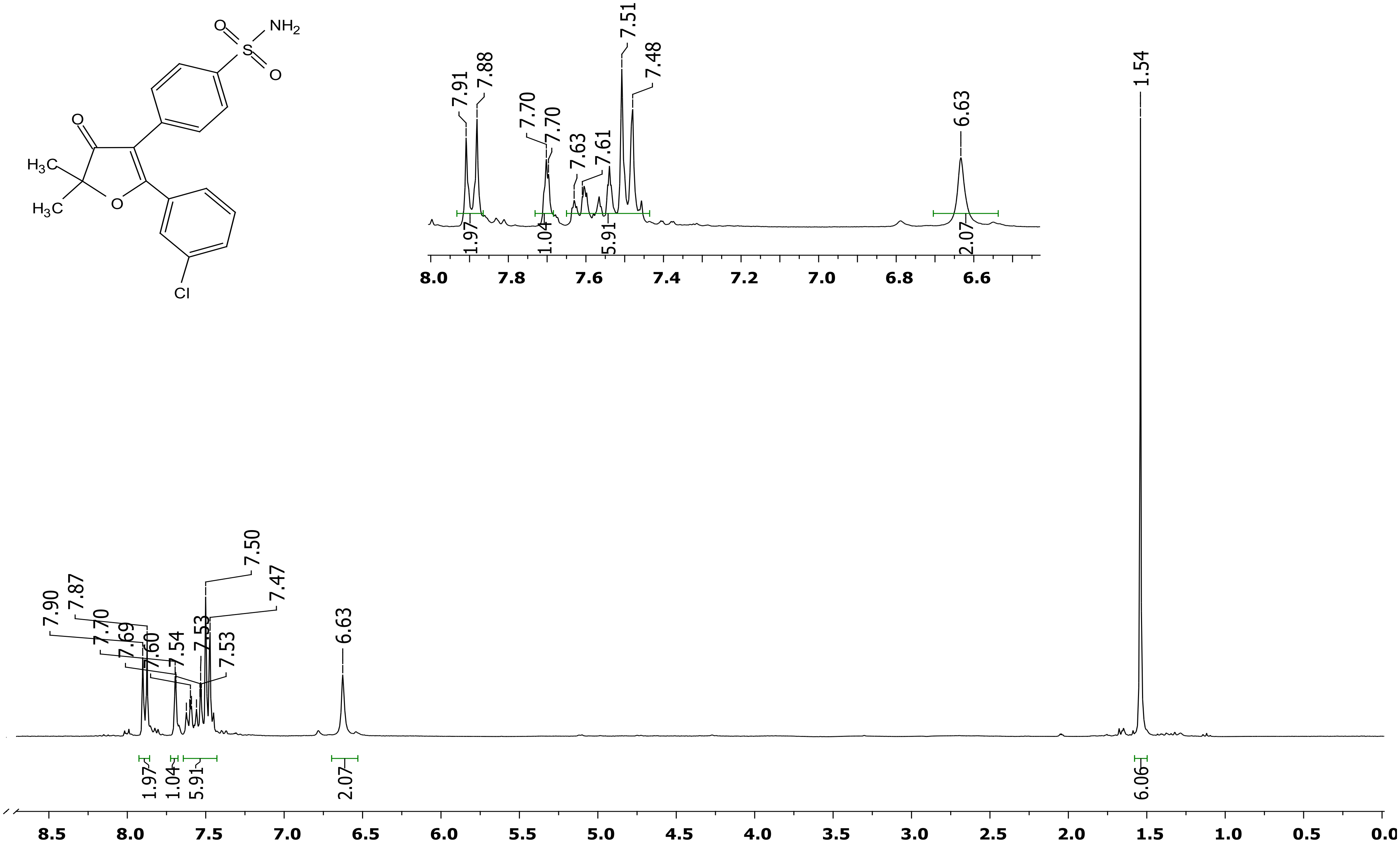
NH2
O
S
O
O
H3C
H3C
O
Cl
7.91
7.88
7.70
7.70
7.63
7.61
7.51
7.48
6.63
1.97
1.04
5.91
2.07
8.0 7.8 7.6 7.4 7.2 7.0 6.8 6.6
1.54
7.90
7.87
7.70
7.69
7.60
7.54
7.53
7.53
7.50
7.47
6.63
1.97
1.04
5.91
2.07
6.06
8.5 8.0 7.5 7.0 6.5 6.0 5.5 5.0 4.5 4.0 3.5 3.0 2.5 2.0 1.5 1.0 0.5 0.0

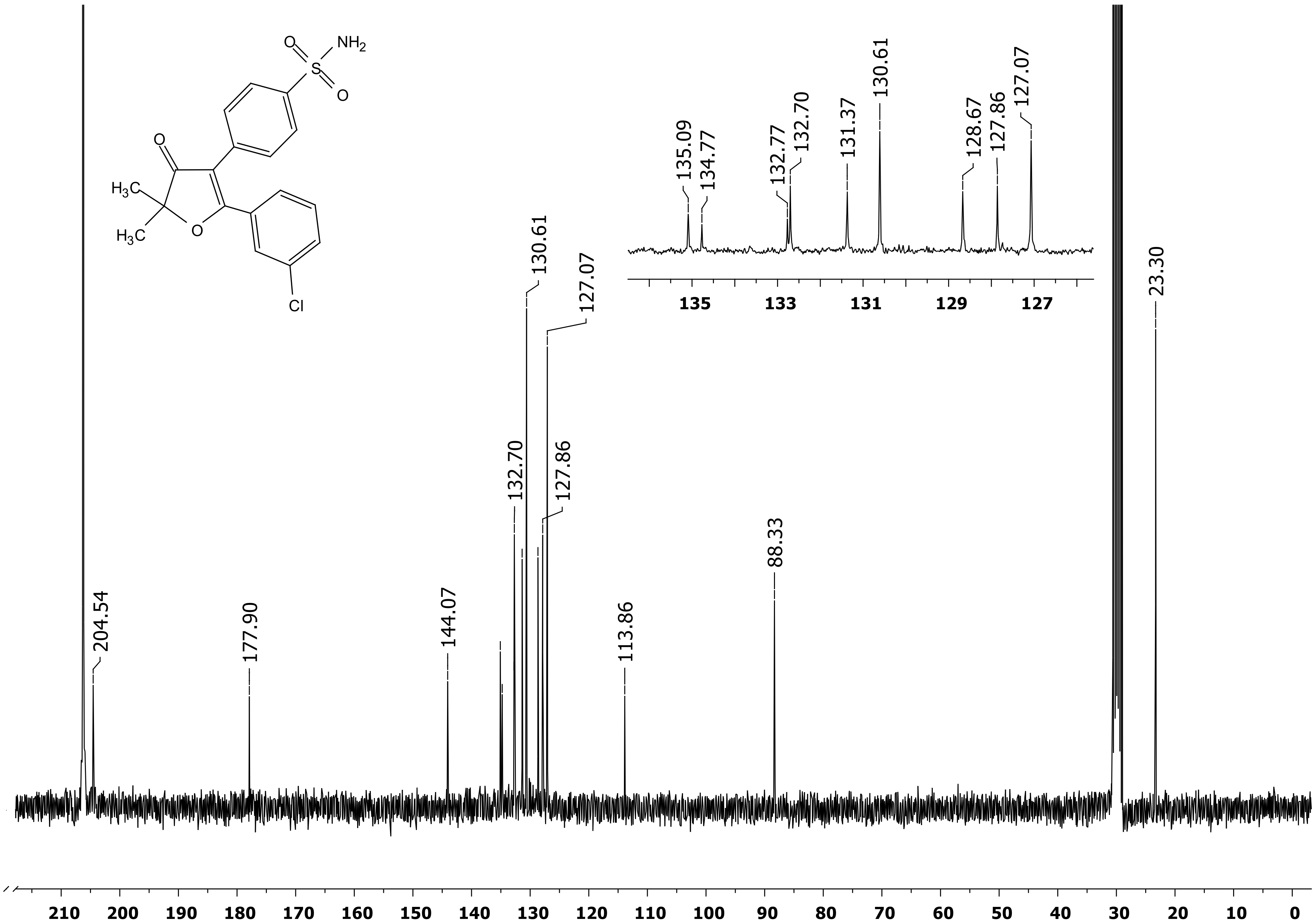
O
NH2
S
O
O
H3C
H3C
O
Cl
135.09
134.77
132.77
132.70
131.37
130.61
128.67
127.86
127.07
135
133
131
129
127
204.54
177.90
144.07
132.70
130.61
127.86
127.07
113.86
88.33
23.30
210
200
190
180
170
160
150
140
130
120
110
100
90
80
70
60
50
40
30
20
10
0

YMEc
YMEc, 357, BF = 100.612769 MHz, Solvent - CDCl3, 22 Jul 2015 T=296 K

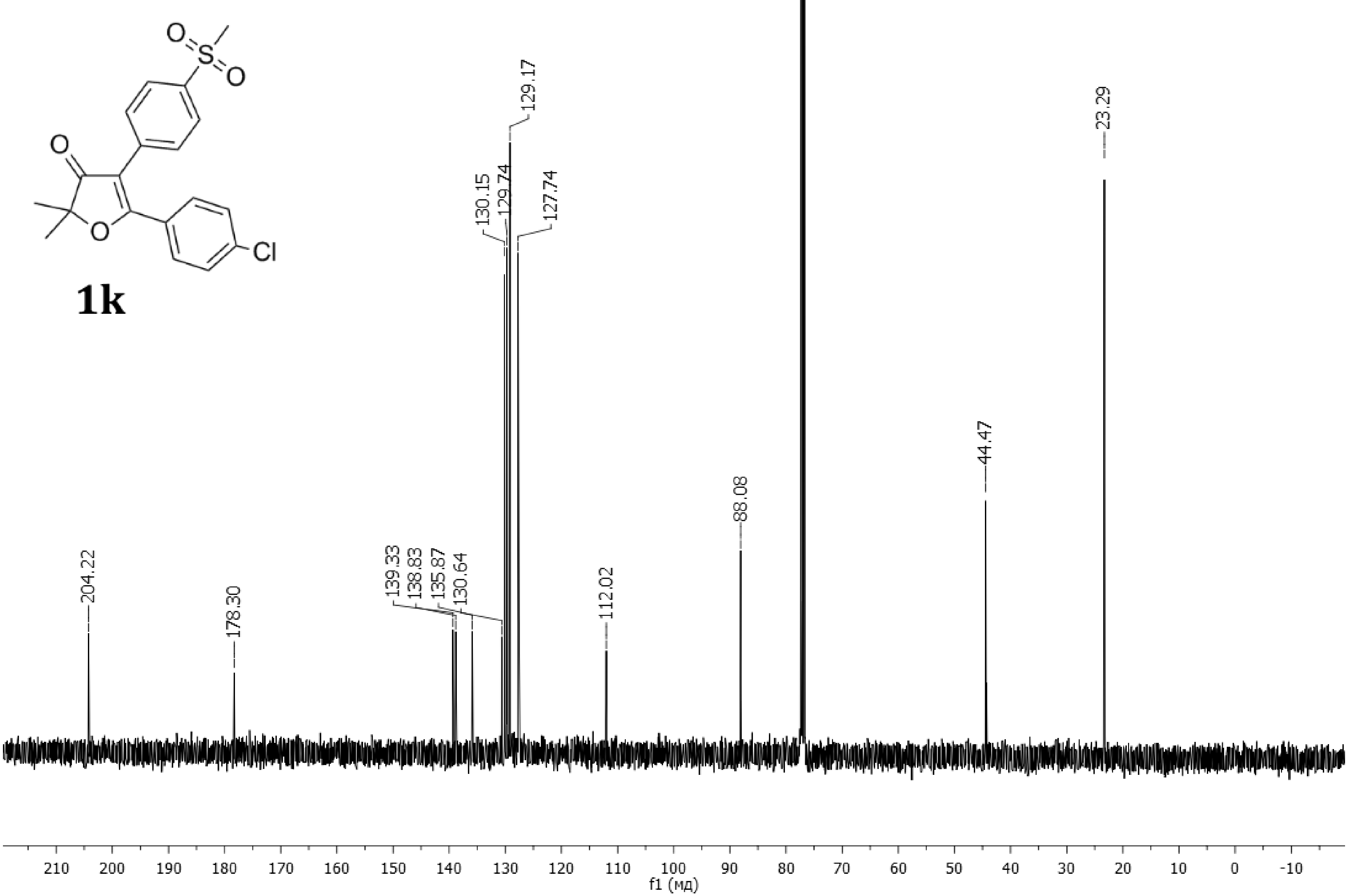

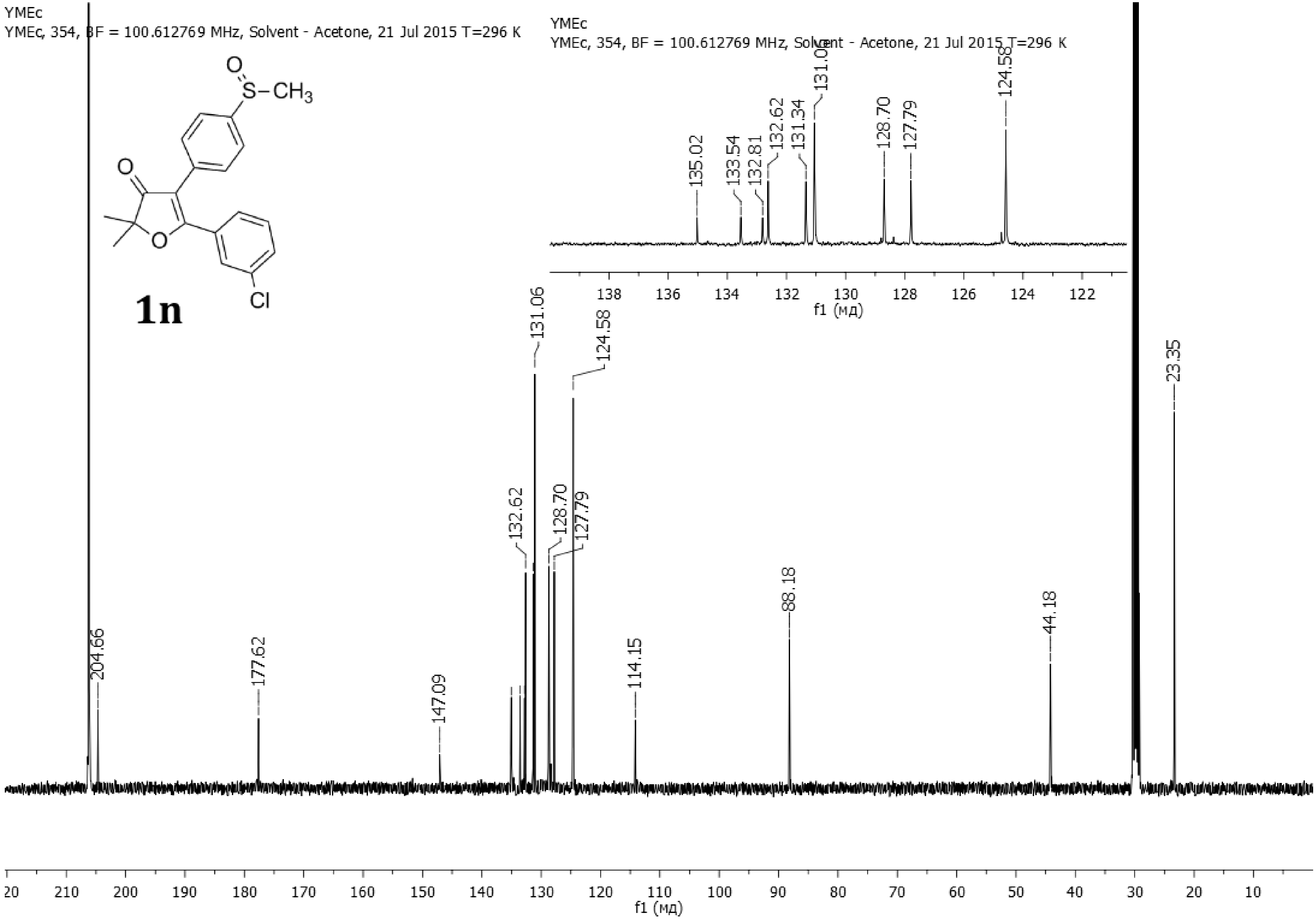

YMEc
YMEc, 354, BF = 100.612769 MHz, Solvent - Acetone, 21 Jul 2015 T=296 K
1n
135.02
133.54
132.81
132.62
131.34
131.06
128.70
127.79
124.58
f1 (мд)
204.66
177.62
147.09
114.15
88.18
44.18
23.35
f1 (мд)

YMEc
YMEc, 358, BF = 100.612769 MHz, Solvent - CDCl3, 22 Jul 2015 T=296 K

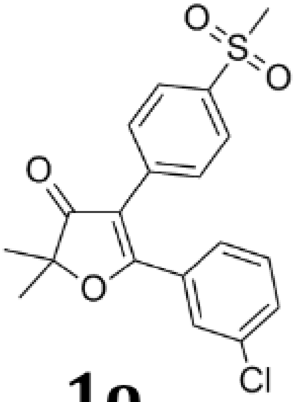

1o

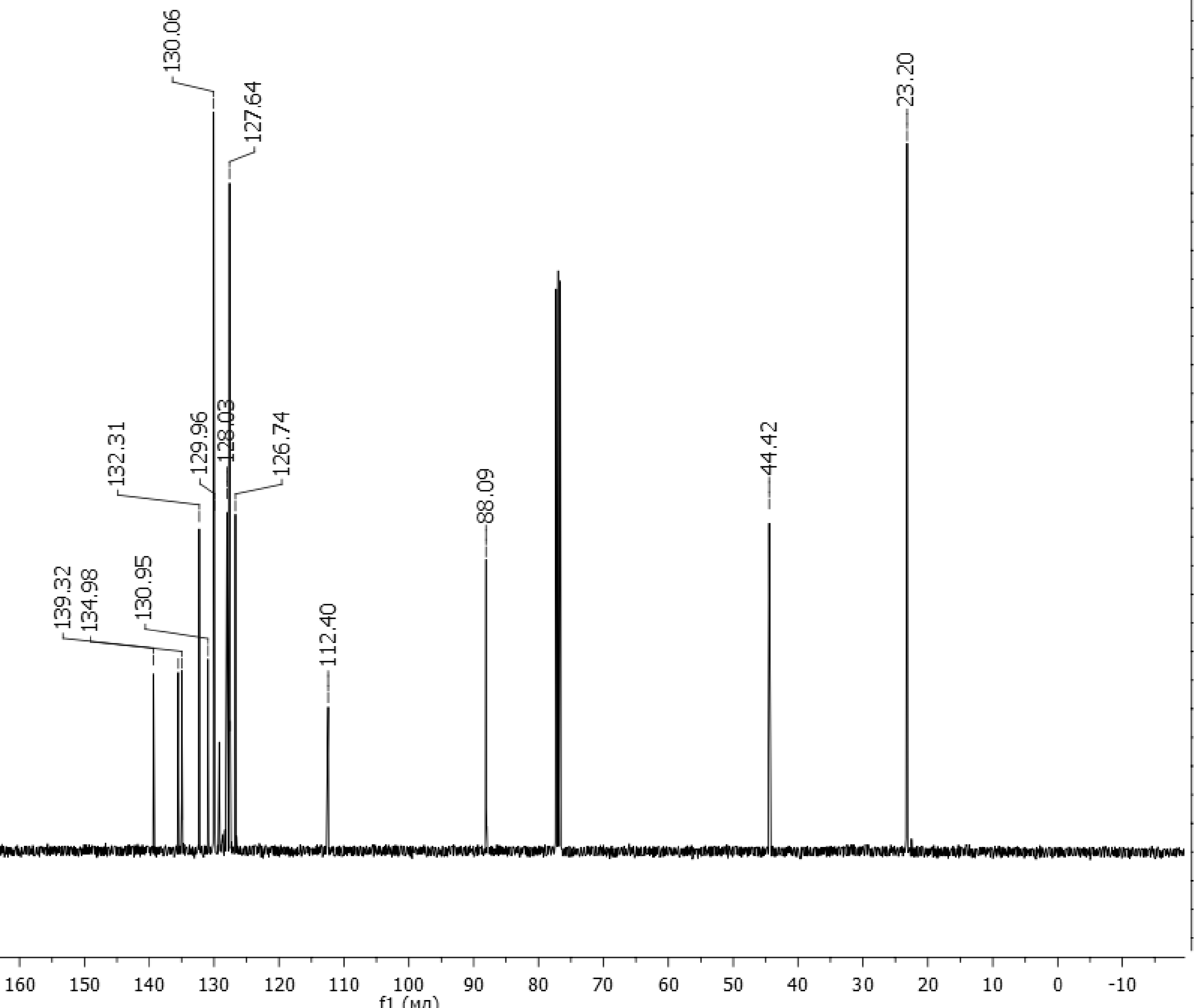

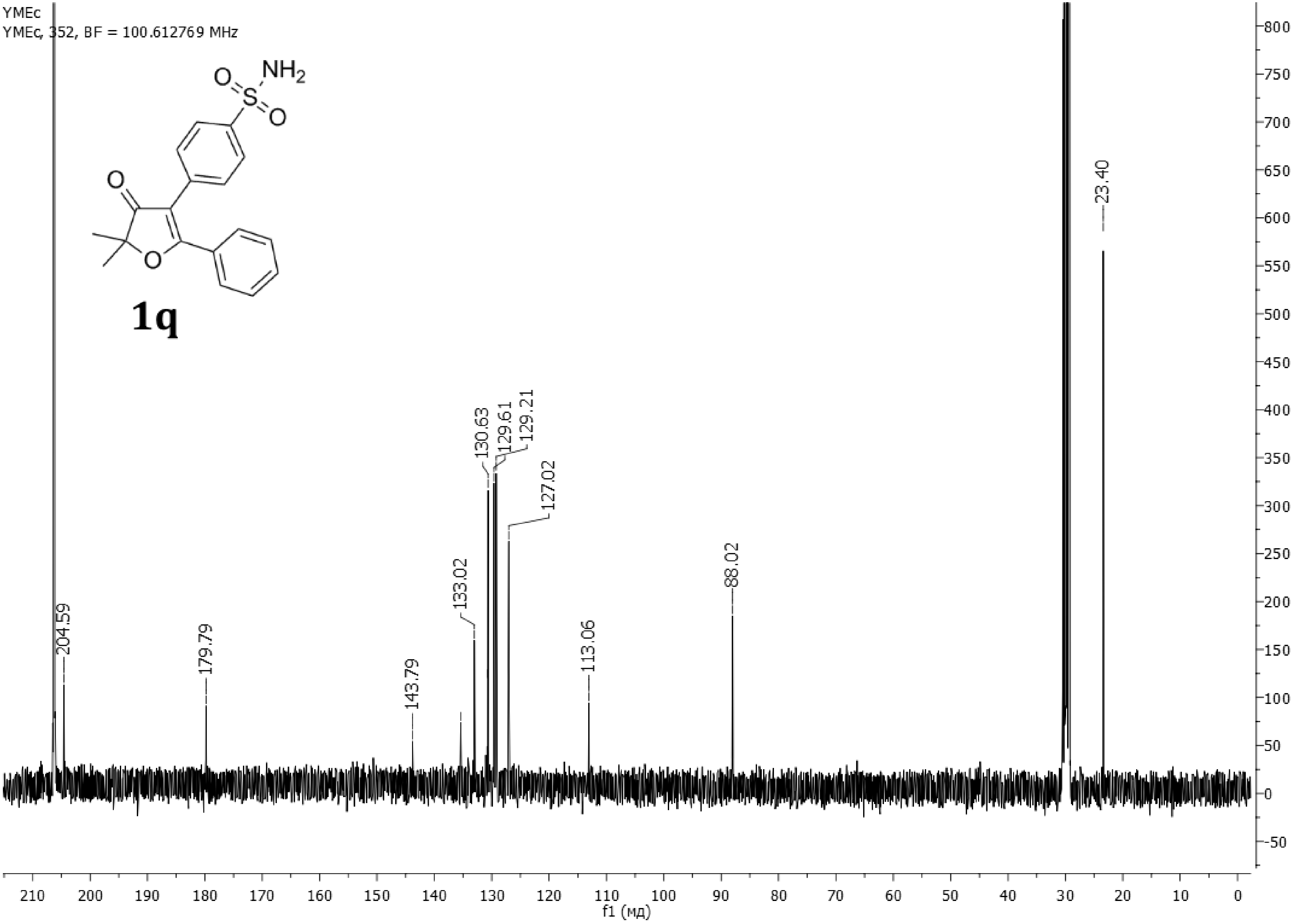
YMEc
YMEc, 352, BF = 100.612769 MHz
NH2
O
S
O
O
O
1q
204.59
179.79
143.79
133.02
130.63
129.61
129.21
127.02
113.06
88.02
23.40
f1 (мд)

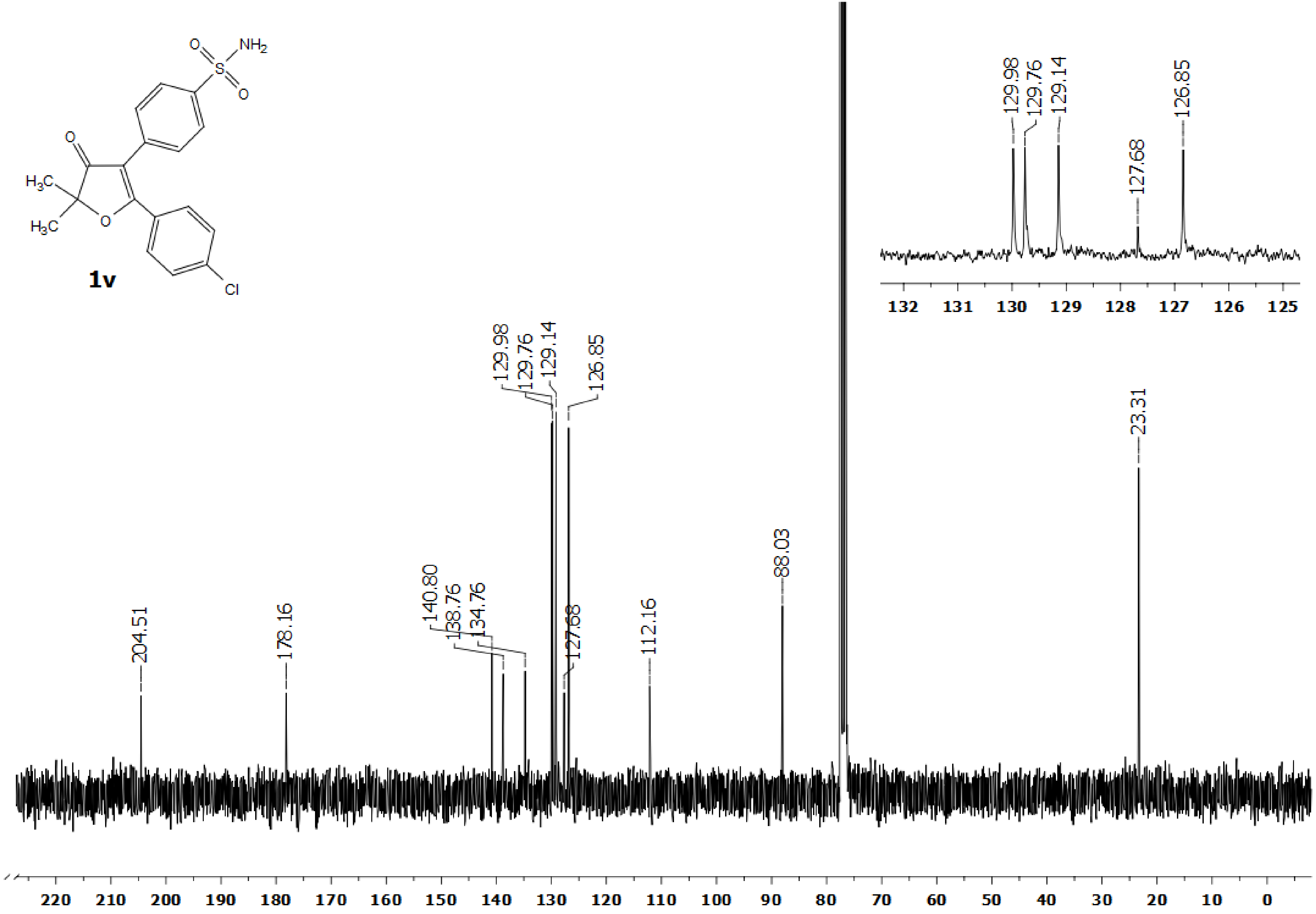
O
NH2
S
O
O
H3C
H3C
O
Cl
204.51
178.16
140.80
138.76
134.76
129.98
129.76
129.14
127.68
126.85
112.16
88.03
23.31
220 210 200 190 180 170 160 150 140 130 120 110 100 90 80 70 60 50 40 30 20 10 0
129.98
129.76
129.14
127.68
126.85
132 131 130 129 128 127 126 125

1v

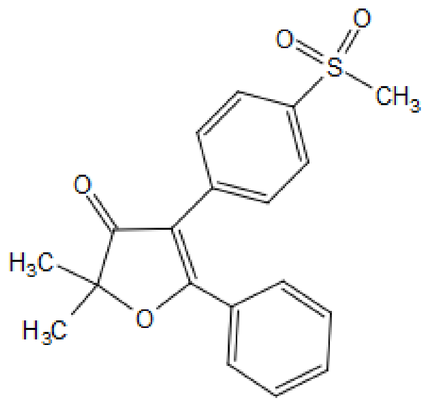

1c

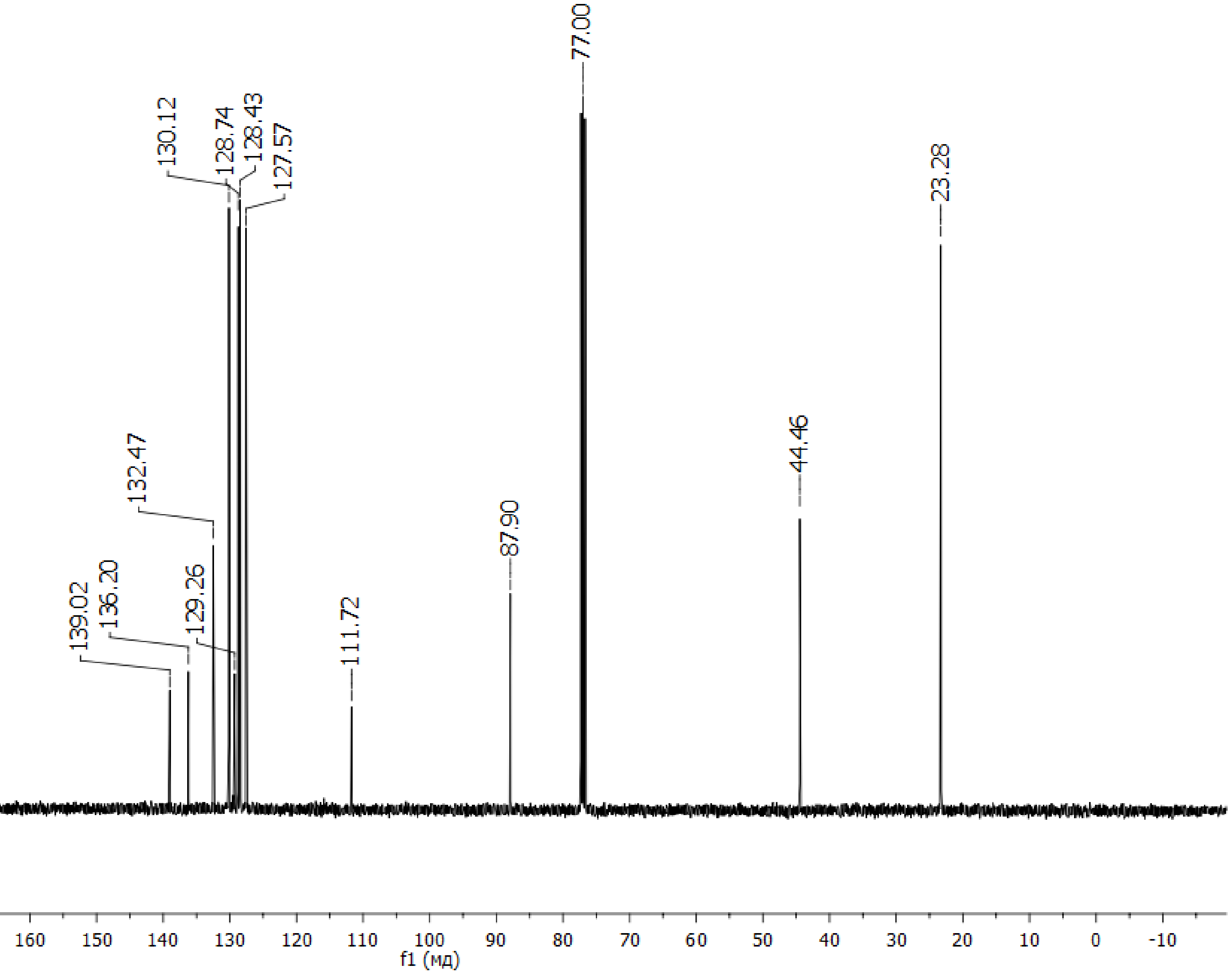
204.37
179.79
139.02
136.20
132.47
130.12
129.26
128.74
128.43
127.57
111.72
87.90
77.00
44.46
23.28
210 200 190 180 170 160 150 140 130 120 110 100 90 80 70 60 50 40 30 20 10 0 -10
f1 (мд)

YMEc
YMEc, 356, BF = 100.612769 MHz, Solvent - CDCl3, 22 Jul 2015 T=296 K

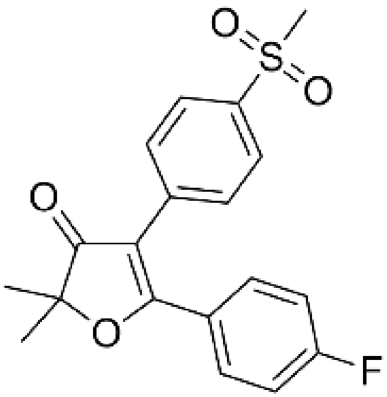

1h

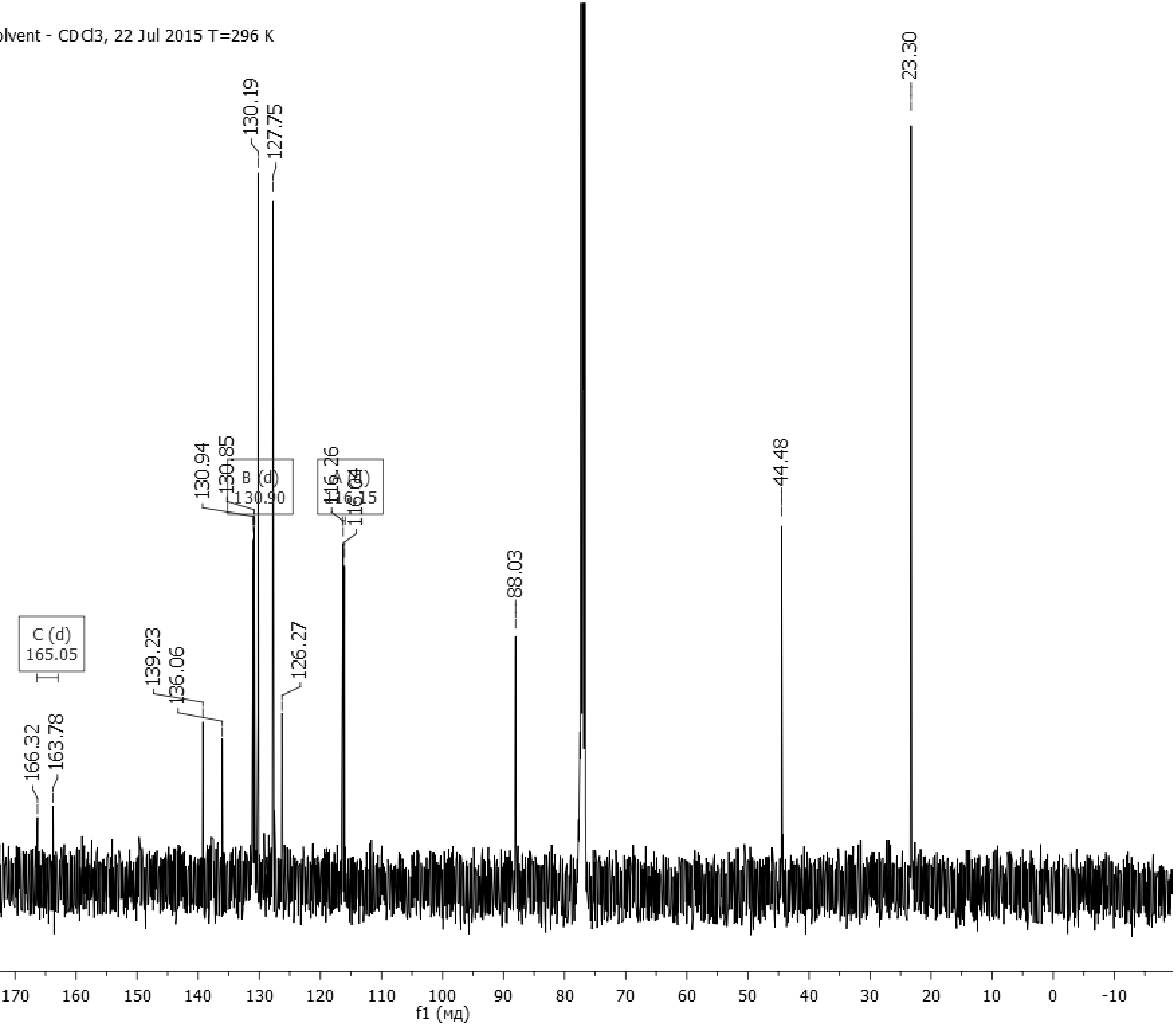

Supplement: Supplementary file 1 [file molecules-24-01751-s001.pdf]
